# Supplementary figures and images for: Diversity of trypanosomes in humans and cattle in the HAT foci Mandoul and Maro, Southern Chad—A matter of concern for zoonotic potential?
Source: PLoS Negl Trop Dis. 2021 Jun 9;15(6):e0009323. doi: 10.1371/journal.pntd.0009323 (PMC8224965; doi:10.1371/journal.pntd.0009323)

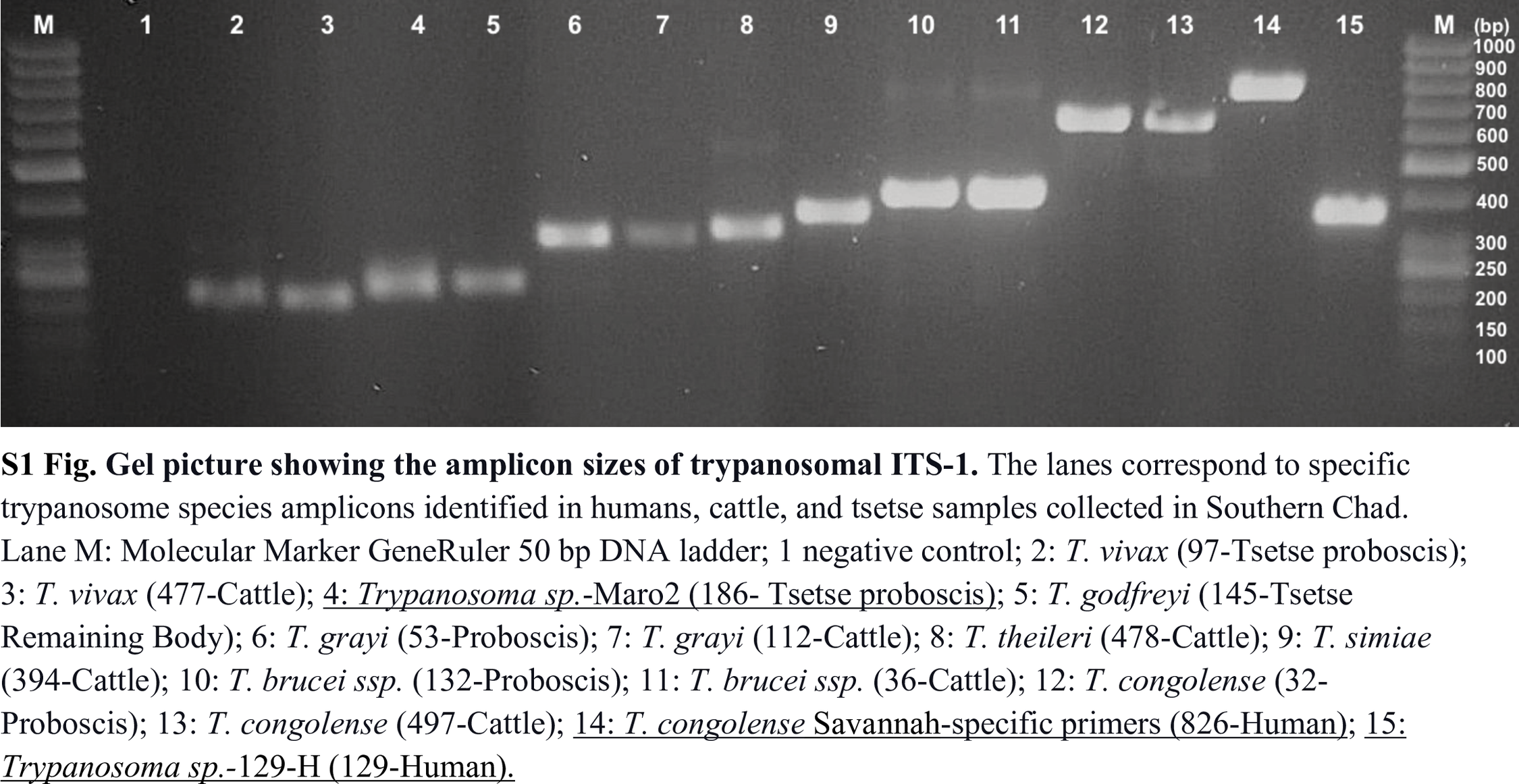

Supplement: S1 Fig — (TIF) [file pntd.0009323.s002.tif]

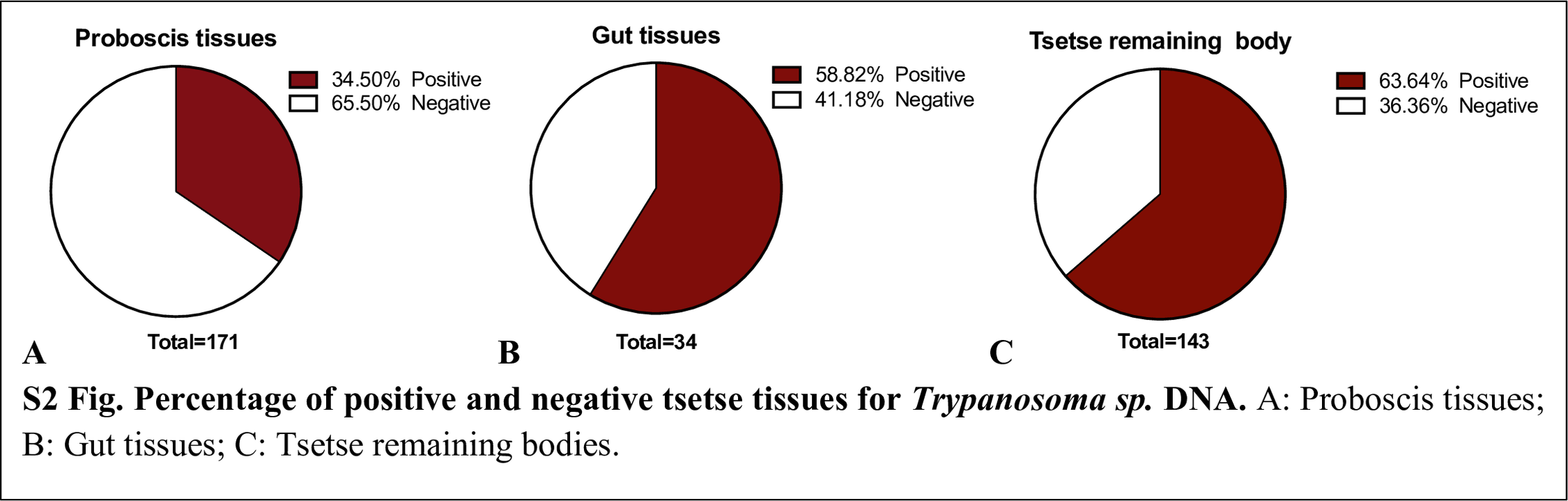

Supplement: S2 Fig — (TIF) [file pntd.0009323.s003.tif]
