# Supplementary material for: Diversity of trypanosomes in humans and cattle in the HAT foci Mandoul and Maro, Southern Chad—A matter of concern for zoonotic potential?
Source: PLoS Negl Trop Dis. 2021 Jun 9;15(6):e0009323. doi: 10.1371/journal.pntd.0009323 (PMC8224965; doi:10.1371/journal.pntd.0009323)
Supplement: S1 Text — (PDF) [file pntd.0009323.s004.pdf]

S1 Text. Study areas description

Mandoul and Maro, located in southern Chad, belong to Sudano-Guinean savannah, geo-ecological zone where the annual rainfall is between 1000 mm and 1300 mm (PANA, 2009). The rainy season extends from May to October and the dry season from November to April in these zones.

The Mandoul, meaning “black river” in Sara, a locally spoken language, gives its name to the focus. It lies at N8° 12.375' E17° 08.063', in the Department of Kouh-Est, Logone-Oriental province. This historical focus delimited in 2003 is a fairly small area, about 15 km wide and 20 km long, but has about 20 000 inhabitants (Louis Fj, 2008). This translates to a density of around 67 inhabitants per km^2 when that of the whole country does not exceed 8 inhabitants per km^2 (Louis Fj, 2008). It is an area in perpetual demographic expansion (Mallaye et al., 2014), and the recent census, in the frame of surveillance strategies against HAT, estimates the population to 38 674 inhabitants (Mahamat et al., 2017). Through its wooden savanna characterised by riparian forests and gallery forests (Mahamat et al., 2017), the Mandoul river banks offer shadows leading to a relatively cold environment, one of the preferred tsetse flies’ locations. They are restricted to the swamps formed in the southern limit of the Mandoul river. As the river flows north, the swamp deteriorates into a marsh habitat, unsuitable for tsetse. Therefore, the population is isolated. Vector control operations with the annual deployment of tiny targets started in 2014.

The Maro focus is located in far southern Chad at the border with the Central African Republic (Fig 1) close to one of its protected areas (Bamingui-Bangouran National Parc). It lies at 8° 24.807' N and 18° 46.139' E, in the Department of “La Grande Sido”, Moyen-Chari province. The focus is crossed by many rivers and their multiple tributaries; the most important is the Chari River and its analogous, the Grand Sido, which mark the border with CAR. Tsetse habitat is configured by the thin riverine vegetation along the banks of the rivers. Vector control operations started in the Chadian part in 2018 (after collecting most of the samples for this study), with annual deployments of tiny targets. No similar operations have been implemented across the border. A geographic survey conducted in 2016 by the PNLTHA and IRED teams shows that the focus hosts 14 532 inhabitants distributed in 45 settlements (ISCTRC and PATTEC, 2019), this, without considering the nomadic pastoralists who cross the border in the dry season for grazing reason.

Reference

ISCTRC, PATTEC, 2019. 35TH GENERAL CONFERENCE OF THE INTERNATIONAL SCIENTIFIC COUNCIL FOR TRYPANOSOMIASIS RESEARCH AND CONTROL (ISCTRC) AND 18TH PATTEC NATIONAL COORDINATORS MEETING.

Louis Fj, L., 2008. Le foyer de trypanosomiase humaine africaine du Mandoul au Tchad : de l’évaluation au contrôle 6.

Mahamat, M.H., Peka, M., Rayaisse, J.-B., Rock, K.S., Toko, M.A., Darnas, J., Brahim, G.M., Alkatib, A.B., Yoni, W., Tirados, I., Courtin, F., Brand, S.P.C., Nersy, C., Alfaroukh, I.O., Torr, S.J., Lehane, M.J., Solano, P., 2017. Adding tsetse control to medical activities contributes to decreasing transmission of sleeping sickness in the Mandoul focus (Chad). PLOS Neglected Tropical Diseases 11, e0005792. https://doi.org/10.1371/journal.pntd.0005792

Mallaye, P., Kohagne Tongué, L., Ndeledje, N., Louis, F.J., Mahamat Hassane, H., 2014. Transmission concomitante de trypanosomose humaine et animale : le foyer de Mandoul au Tchad. Revue d’élevage et de médecine vétérinaire des pays tropicaux 67, 5. https://doi.org/10.19182/remvt.10154

PANA, 2009. Programme d’Action National d’Adaptation (PANA) aux Changements Climatiques.
