# Supplementary material for: Diversity of trypanosomes in humans and cattle in the HAT foci Mandoul and Maro, Southern Chad—A matter of concern for zoonotic potential?
Source: PLoS Negl Trop Dis. 2021 Jun 9;15(6):e0009323. doi: 10.1371/journal.pntd.0009323 (PMC8224965; doi:10.1371/journal.pntd.0009323)
Supplement: S2 Text — (PDF) [file pntd.0009323.s005.pdf]

S2 Text. Samples collections and processing

- ✓ **Human surveys**

The study was conducted to determine the circulating trypanosomes and their genetic diversity in humans in the Mandoul and the Maro sleeping sickness foci. Surveys were conducted in February 2017, March, and June 2018. In each of the two surveyed areas, eight randomly selected villages were visited and a military camp included at the request of its inhabitants. The chosen villages had included nomadic and sedentary communities. Once villages were selected, survey team members visited the village together with a local translator to introduce themselves and acquire the list of households constituting the village. All the heads of households and their members present in the village were invited to an open meeting where the study's aims were carefully explained and translated in the local languages. Before these large meetings, contact visits with the local and administrative authorities were established, and sensitisations were primed.

In order to proceed with the selection, households were numbered, and a total of 6 to 16 households in a selected village were drawn for participation. A chosen household included all its members automatically. The number of households and participants surveyed per village depended on the size of its population and the individuals who consent for participation. However, we collected no blood from children under 5 years old.

The open-source Epidemiologic statistics for public health software Version 3.01 (http://www.openepi.com/SampleSize/SSPropor.htm) and the automated software program (Raosoft sample size calculator for study: http://www.raosoft.com/samplesize.html) were used to estimate the human sample size that should be included in the survey. Based on the estimated population recorded from the institutions in charge of HAT control in Chad which are reported later (ISCTRC and PATTEC, 2019; Mahamat et al., 2017) and from the heads of the communities (chef de canton de Bembaïtada for the Mandoul area, chef de Canton de Maro et de Gourourou for the Maro area), the Mandoul focus have 114 human settlements with 38,674. In comparison, Maro has 45 settlements with 14,532 inhabitants in 2017, as stated above. The present study used these numbers as the total populations from which the sample sizes were calculated. With an accepted margin of error of 5% and a 95% confidence interval, the sample sizes required were 381 in the Mandoul and 375 in the Maro.

- ✓ **Cattle surveys**

We surveyed the Mandoul and Maro foci in January, March, June, and November 2018 to investigate the presence and the distribution of trypanosomes circulating in cattle in the areas. Sedentary villages, semi-nomadic camps, a nomadic settlement, and a refugee camp were included in the surveys. Some of the villages were the same as those visited for human. Representative cattle, from each herd randomly chosen, were included in this study. However, the animals were partly chosen by the herdsmen themselves.

Mandoul has about 14,000 cattle (Rayaisse et al., 2020), while Maro has over 55,600 (IOM•OIM, 2019). The open-source Epidemiologic statistics for public health software Version 3.01 was used to estimate the sample size of the study.

The questionnaires were addressing the number of animals in the herd, the breeding system, breeds, source of water and nutritional support, health status including symptoms, morbidity and mortality, animal vaccinal status, sex, age of the animals and level of education of the herdsman. The age of the animal was given by the herdsmen and to some extent confirmed by the veterinarian present during the fieldwork. They are grouped in three categories young (<2.5 years), mature (between 2.5 and 5 years), and elders (above 5 years). Thus, the relative age given by the herdsmen will be within the age set subsequently. Questionnaires were answered by each herdsman during the same day of blood collection.

✓ **Blood collection and processing**

About 5 to 7 mL of blood was collected from the radial vein (venipuncture) of each human participant using vacutainer butterfly needles. From 7 to 10 mL was taken from the jugular vein of each animal using a syringe. Collected blood was then directly transferred/connected into a labelled blood collection tube (or vacutainer tube) containing EDTA. The tubes containing blood were transported in a cold box to the camp where a mobile laboratory was set up. 200 µL of whole blood were pipetted into 1.5 mL labelled cryotube, and 50 µL were added to 150 µL of Nucleic Acid Preservative Agent, NAPA (25 mM sodium citrate, 10 mM EDTA, 5.3 M ammonium sulfate, pH 7.5), in a separate cryotube.

✓ **Tsetse trapping and processing**

Biconical traps were used for this study. They were set up in the shade at riversides by prioritising adjacent sites to the villages or the animal grazing/watering spots. Location of each trap, time of setting and harvesting were documented using a GPS device (Garmin GPS map 64s) and a camera. Ambient temperature, relative humidity as well as air pressure were recorded using data loggers (date logger LOG32 THP, Dostmann electronic GmbH, Germany). The flies trapped were collected the next day (after approximately 24h) and transported to the camp in cold boxes, where they were dissected. The number of flies collected per trap, their sex and their nutritional status (teneral or non-teneral) were also recorded. Each tube aimed to contain *Glossina* sp. tissue was labelled accordingly, and the fly morphologically identified before dissected.

Wings, legs, proboscises were collected from all flies and the remaining body (TRB) of dead tsetse flies were kept in 500 µL pure ethanol. The guts and salivary glands were dissected and kept separately in a 1.5 mL labelled cryotube from live flies.

Wings were removed first and kept dry for geometric morphology analysis. Legs and proboscis were stored in 200 µL NAPA. Gut tissues were dissected last and homogenised in 200 µL 50 mM Tris-HCl pH 9.0 by vortexing for one minute using four 2.38 mm metal beads (MoBio Laboratories, Carlsbad, CA, USA). 50 µL of the homogenised tissue was then added to 500 µL NAPA, kept for DNA extraction (Ngomtcho et al., 2017; Weber et al., 2019).

To avoid contamination between tissues, the proboscis was removed first before opening the abdomen. Fresh dissection pins and forceps were used for each fly tissue and a new slide for each fly. To decontaminate the material for the next survey, pins and forceps were incubated overnight in 5% sodium hydrochloride solution and subsequently washed thoroughly with double distilled water.

Samples were all kept at -20°C in the field and transported in a cold chain using frozen salt-water containing bottles. The ambient temperature of the boxes (with samples) is recorded with the data logger during the trip. For long term storage, samples were kept at -80°C in the laboratory.

Reference

IOM•OIM, 2019. TRANSHUMANCE TRACKING TOOL (TTT) CHAD – DÉPARTEMENT OF GRANDE SIDO_DASHBOARD #2.

ISCTRC, PATTEC, 2019. 35TH GENERAL CONFERENCE OF THE INTERNATIONAL SCIENTIFIC COUNCIL FOR TRYPANOSOMIASIS RESEARCH AND CONTROL (ISCTRC) AND 18TH PATTEC NATIONAL COORDINATORS MEETING.

Mahamat, M.H., Peka, M., Rayaisse, J.-B., Rock, K.S., Toko, M.A., Darnas, J., Brahim, G.M., Alkatib, A.B., Yoni, W., Tirados, I., Courtin, F., Brand, S.P.C., Nersy, C., Alfaroukh, I.O., Torr, S.J., Lehane, M.J., Solano, P., 2017. Adding tsetse control to medical activities contributes to decreasing transmission of sleeping sickness in the Mandoul focus (Chad). PLOS Neglected Tropical Diseases 11, e0005792. https://doi.org/10.1371/journal.pntd.0005792

Ngomtcho, S.C.H., Weber, J.S., Ngo Bum, E., Gbem, T.T., Kelm, S., Achukwi, M.D., 2017. Molecular screening of tsetse flies and cattle reveal different Trypanosoma species including T. grayi and T. theileri in northern Cameroon. Parasites & Vectors 10. https://doi.org/10.1186/s13071-017-2540-7

Rayaisse, J.-B., Courtin, F., Mahamat, M.H., Chérif, M., Yoni, W., Gadjibet, N.M.O., Peka, M., Solano, P., Torr, S.J., Shaw, A.P.M., 2020. Delivering 'tiny targets' in a remote region of southern Chad: a cost analysis of tsetse control in the Mandoul sleeping sickness focus. Parasites & Vectors 13. https://doi.org/10.1186/s13071-020-04286-w

Weber, J.S., Ngomtcho, S.C.H., Shaida, S.S., Chechet, G.D., Gbem, T.T., Nok, J.A., Mamman, M., Achukwi, D.M., Kelm, S., 2019. Genetic diversity of trypanosome species in tsetse flies (Glossina spp.) in Nigeria. Parasites & Vectors 12. https://doi.org/10.1186/s13071-019-3718-y
