# Supplementary material for: Diversity of trypanosomes in humans and cattle in the HAT foci Mandoul and Maro, Southern Chad—A matter of concern for zoonotic potential?
Source: PLoS Negl Trop Dis. 2021 Jun 9;15(6):e0009323. doi: 10.1371/journal.pntd.0009323 (PMC8224965; doi:10.1371/journal.pntd.0009323)
Supplement: S3 Text — (PDF) [file pntd.0009323.s006.pdf]

S3 Text. Molecular amplification and identification procedure, and subcloning and sequencing of amplicons.

ITS1 nested PCR was performed using 25 µL of a master mix containing 2 µM of each outer primer (Sigma-Aldrich, Darmstadt, Germany), 200 µM dNTPs, 2.5 Units Dream*Taq* polymerase, 1x Dream*Taq* buffer (all from Thermo Scientific) and 1µL of human/cattle template DNA, TBR or proboscis DNA template and 5 µL of tsetse gut DNA template (Ngomtcho et al., 2017; Weber et al., 2019). The product of the first reaction was diluted 1:80 and 1 µL of the dilution used for the second reaction, which was prepared similarly as the first, except inner primers were used in this case. The cycling conditions for both reactions were as follows: initial denaturation at 95°C for 3 min followed by 30 cycles at 94°C for 60 s, 54°C for 30 s, 72°C for 30 s and final elongation at 72°C for 5 min. This is also valid for *T. congolense* specific identification while using specific primers. 12 µL of each PCR product was separated on 1.5% TBE (Tris-Borate-EDTA buffer) or TAE (Tris-acetate-EDTA) agarose gels stained with Stain-G (SERVA, Heidelberg, Germany) for 1 h at 100V. 5 µL molecular marker (GeneRuler DNA ladder, Thermo scientific) was loaded beside the samples in order to estimate the sizes of expected amplicon fragments.

For confirmation of the ITS1 analysis and performing of phylogenetic analyses, a nested PCR targeting the partial *gGAPDH* gene was carried out (Hamilton et al., 2004; Weber et al., 2019). *gGAPDH* is ubiquitous, essential glycolytic enzyme and have a slow rate of molecular evolution, making it suitable for studying evolution over large time-scales (Hamilton et al., 2004). Therefore, it has been a marker of choice for phylogenetic analysis (Hamilton et al., 2004; Hannaert et al., 1998; Paguem et al., 2019).
A master mix was prepared as described for ITS-1 nested PCR except for the respective primers (Hamilton et al., 2004; Weber et al., 2019). Reaction conditions were 95°C for 3 min, followed by 30 cycles at 95°C for 1 min, 55°C for 30 s, and a final extension at 72°C for 10 min. 1 µL of undiluted PCR product when analysing human/cattle samples and 5 µL when testing tsetse samples, was then used in the second reaction with a master mix containing the inner primers (Weber et al., 2019). Similar conditions as in the first reaction were used, except the annealing temperature changed to 52°C. PCR products were separated by gel electrophoresis as described above.

Selected PCR products of amplified gene fragments were excised from the agarose gel and purified using GeneJET Gel Extraction Kit (Thermo Fisher Scientific) following the manufacturer's instructions. DNA concentration was estimated using a Nanodrop 1000 (Thermo Fisher Scientific, Dreieich, Germany). The purified DNA, mixed with one of the inner primers, was sent for Sanger sequencing to a commercial provider (Microsynth SeqLab, Göttingen, Germany).

Concerning the ITS1 PCR product, the purified DNA was subcloned into a linearised pJET 1.2/blunt plasmid using the CloneJET PCR Cloning Kit (Thermo Fisher Scientific) according to the manufacturer's instructions. Singles clones were checked by colony PCR using inner ITS1 primers. Positive clones were then picked up and cultured overnight at 37°C while shaking (220 rpm) in 5 mL LB medium plus 100 µg/mL ampicillin. On the following day, the cultures

were purified using GeneJET Plasmid Miniprep Kit (Thermo Fisher Scientific). Purified DNA was quantified and sent for sequencing according to the manufacturer's instructions.

Reference

Hamilton, P.B., Stevens, J.R., Gaunt, M.W., Gidley, J., Gibson, W.C., 2004. Trypanosomes are monophyletic: evidence from genes for glyceraldehyde phosphate dehydrogenase and small subunit ribosomal RNA. International Journal for Parasitology 34, 1393–1404. https://doi.org/10.1016/j.ijpara.2004.08.011

Hannaert, V., Opperdoes, F.R., Michels, P.A.M., 1998. Comparison and Evolutionary Analysis of the Glycosomal Glyceraldehyde-3-Phosphate Dehydrogenase from Different Kinetoplastida. Journal of Molecular Evolution 47, 728–738. https://doi.org/10.1007/PL00006432

Ngomtcho, S.C.H., Weber, J.S., Ngo Bum, E., Gbem, T.T., Kelm, S., Achukwi, M.D., 2017. Molecular screening of tsetse flies and cattle reveal different Trypanosoma species including T. grayi and T. theileri in northern Cameroon. Parasites & Vectors 10. https://doi.org/10.1186/s13071-017-2540-7

Paguem, A., Abanda, B., Ndjonka, D., Weber, J.S., Ngomtcho, S.C.H., Manchang, K.T., Adoulmoumini, M., Eisenbarth, A., Renz, A., Kelm, S., Achukwi, M.D., 2019. Widespread co-endemicity of Trypanosoma species infecting cattle in the Sudano-Sahelian and Guinea Savannah zones of Cameroon. BMC Veterinary Research 15. https://doi.org/10.1186/s12917-019-2111-6

Weber, J.S., Ngomtcho, S.C.H., Shaida, S.S., Chechet, G.D., Gbem, T.T., Nok, J.A., Mamman, M., Achukwi, D.M., Kelm, S., 2019. Genetic diversity of trypanosome species in tsetse flies (Glossina spp.) in Nigeria. Parasites & Vectors 12. https://doi.org/10.1186/s13071-019-3718-y
