# Supplementary material for: Diversity of trypanosomes in humans and cattle in the HAT foci Mandoul and Maro, Southern Chad—A matter of concern for zoonotic potential?
Source: PLoS Negl Trop Dis. 2021 Jun 9;15(6):e0009323. doi: 10.1371/journal.pntd.0009323 (PMC8224965; doi:10.1371/journal.pntd.0009323)
Supplement: S1 Table — (PDF) [file pntd.0009323.s007.pdf]

S1 Table. Generic and specific primers used in the study.

Primer name	*Trypanosoma sp.*	Nucleotide sequence (5'-3')	Amplicon size (bp)	Source
**ITS1-OutF**	All trypanosomes	TGCAATTATTGGTCGCGC	100-650	(Adams et al., 2006)
**ITS1-OutR**		CTTTGCTGCGTTCTT	100-650	
**ITS1-InF**		TAGAGGAAGCAAAAG	100-650	
**ITS1-InR**		AAGCCAAGTCATCCATCG	100-650	
**gGAPDH-Out**	All trypanosomes	TTY GCC GYA TYG GYC GCA TGG	816	(Hamilton et al., 2004)
**gGAPDH-Out**		ACMAGRTCCACCACRCGGTG		
**gGAPDH-InR**		GTT YTG CAG SGT CGC CTT GG		
**gGAPDH-InF**		GCSTAYCAGATGAAGTACGAC		(Weber et al., 2019)
**Tcon-outF**	*T. congolense*	TGTTGGTCGACACTGAGA	681-781	(Ngomtcho et al., 2017)
**Tcon-outR**		TGCAATTATTGGTCGCGC		
**Tcon-inF**		TCGCGTGTCTCACGT		
**Tcon-inR**		TCAAAGATTGGGCAATGT		

Reference

Adams, E.R., Malele, I.I., Msangi, A.R., Gibson, W.C., 2006. Trypanosome identification in wild tsetse populations in Tanzania using generic primers to amplify the ribosomal RNA ITS-1 region. Acta Tropica 100, 103–109. https://doi.org/10.1016/j.actatropica.2006.10.002

Hamilton, P.B., Stevens, J.R., Gaunt, M.W., Gidley, J., Gibson, W.C., 2004. Trypanosomes are monophyletic: evidence from genes for glyceraldehyde phosphate dehydrogenase and small subunit ribosomal RNA. International Journal for Parasitology 34, 1393–1404. https://doi.org/10.1016/j.ijpara.2004.08.011

Ngomtcho, S.C.H., Weber, J.S., Ngo Bum, E., Gbem, T.T., Kelm, S., Achukwi, M.D., 2017. Molecular screening of tsetse flies and cattle reveal different Trypanosoma species including T. grayi and T. theileri in northern Cameroon. Parasites & Vectors 10. https://doi.org/10.1186/s13071-017-2540-7

Weber, J.S., Ngomtcho, S.C.H., Shaida, S.S., Chechet, G.D., Gbem, T.T., Nok, J.A., Mamman, M., Achukwi, D.M., Kelm, S., 2019. Genetic diversity of trypanosome species in tsetse flies (Glossina spp.) in Nigeria. Parasites & Vectors 12. https://doi.org/10.1186/s13071-019-3718-y
