# Supplementary material for: Diversity of trypanosomes in humans and cattle in the HAT foci Mandoul and Maro, Southern Chad—A matter of concern for zoonotic potential?
Source: PLoS Negl Trop Dis. 2021 Jun 9;15(6):e0009323. doi: 10.1371/journal.pntd.0009323 (PMC8224965; doi:10.1371/journal.pntd.0009323)
Supplement: S2 Table — (PDF) [file pntd.0009323.s008.pdf]

S2 Table. Selected villages in the Mandoul and Maro HAT foci for human blood samples collection.

Focus	Village	Household	Registered	Blood collected
Mandoul	Bembaïtada	6	62	52
Mandoul	Konael	8	68	42
Mandoul	Kobiteye	7	51	38
Mandoul	Dedaye I	8	45	38
Mandoul	Ferrick Sandana Lelou	8	30	24
Mandoul	Kousserie	13	48	32
Mandoul	Palkoyo	14	50	34
Mandoul	Berayan	18	55	46
**Total Mandoul**	**8**	**82**	**409**	**306**
Maro	Ngakorio	17	84	63
Maro	Ferrick Hanno	11	55	51
Maro	Kobdogué	14	57	54
Maro	Ngon-Molo	8	43	39
Maro	Beguiyon	11	35	30
Maro	Baguirgué	15	55	50
Maro	Aldjazira	24	73	70
Maro	Guirkyon (village)	19	59	35
Maro	Guirkyon (Army camp)	-	19	19
**Total Maro**	**9**	**119**	**480**	**411**
**Total**	**17**	**182**	**889**	**717**
