# Supplementary material for: Diversity of trypanosomes in humans and cattle in the HAT foci Mandoul and Maro, Southern Chad—A matter of concern for zoonotic potential?
Source: PLoS Negl Trop Dis. 2021 Jun 9;15(6):e0009323. doi: 10.1371/journal.pntd.0009323 (PMC8224965; doi:10.1371/journal.pntd.0009323)
Supplement: S3 Table — (PDF) [file pntd.0009323.s009.pdf]

S3 Table. Trypanosomes frequency in cattle according to villages and foci, and number of cattle sampled per village. Single, double and triple infections.

	Maro _ Canton Maro							Maro _ Canton Gourourou				Maro Total	Mandoul
***Trypanosoma sp.*/Village**	**1**	**2**	**3**	**4**	**5**	**6**	**7**	**8**	**9**	**10**	**11**		**12**
**Single infection**													
T. congolense	0	0	0	13	1	3	0	17	0	7	0	41 (8.9)	0
T. brucei ssp.	0	0	0	0	0	0	0	0	1	0	0	1 (0.2)	0
T. theileri	5	2	2	1	1	2	1	1	2	3	6	26 (5.6)	61 (78.2)
T. grayi	0	0	1	0	0	0	0	1	1	2	2	7 (1.5)	0
T. godfreyi	0	0	0	2	0	0	0	0	0	1	0	3 (0.6)	0
T. vivax	1	2	0	4	0	15	5	8	3	5	6	49 (10.6)	0
**Total**	**6**	**4**	**3**	**20**	**2**	**20**	**6**	**27**	**7**	**18**	**14**	**127 (27.4)**	**61 (78.2)**
**Double infections**													
T. godfreyi/T. vivax	0	0	0	0	0	0	0	0	2	0	0	2 (0.4)	0
T. grayi/T. vivax	0	0	0	1	0	0	0	0	1	0	0	2 (0.4)	0
T. theileri/T. godfreyi	0	0	0	0	0	0	0	1	0	0	0	1 (0.2)	0
T. theileri/T. vivax	0	0	0	2	1	0	0	0	0	0	2	5 (1.1)	0
T. simiae/T. vivax	0	0	0	0	0	0	0	0	0	0	0	0 (0.0)	3 (3.8)
T. brucei/ T. vivax	0	0	0	0	0	0	0	0	1	0	0	1 (0.2)	0
T. congolense/T. grayi	0	0	0	0	0	0	0	0	0	1	0	1 (0.2)	0
T. congolense/T. theileri	0	0	0	1	0	0	0	0	0	0	2	3 (0.6)	0
T. congolense/T. brucei	0	1	0	2	0	3	5	1	1	2	1	16 (3.5)	0
**Total**	**0**	**1**	**0**	**6**	**1**	**8**	**5**	**2**	**6**	**4**	**5**	**21 (6.6)**	**3 (3.8)**
**Triple infections**													
T. congolense/T. brucei/T. vivax	**0**	**0**	**0**	**0**	**0**	**1**	**0**	**0**	**0**	**0**	**0**	**1 (0.2)**	**0**
**Positive N (%)**	**6 (28.6)**	**5 (25.0)**	**3 (13.6)**	**26 (37.7)**	**3 (5.2)**	**24 (44.4)**	**11 (20.4)**	**29 (49.1)**	**12 (44.4)**	**21 (37.5)**	**19 (86.4)**	**159 (34.4)**	**64 (82.1)**
**Negative N (%)**	**15 (71.4)**	**15 (75.0)**	**19 (86.4)**	**43 (62.3)**	**55 (94.8)**	**30 (55.6)**	**43 (79.6)**	**30 (50.9)**	**15 (55.6)**	**35 (62.5)**	**3 (13.6)**	**303 (65.6)**	**14 (17.9)**
**Number of cattle**	**21**	**20**	**22**	**69**	**58**	**54**	**54**	**59**	**27**	**56**	**22**	**462**	**78**

Villages _ **1** : Ngakorio (Sedentary) ; **2 :** Ridina Al'Az (Semi-nomadic) ; **3 :** Maïngama (Refugee camp) ; **4 :** Aldjazira1 (Semi-nomadic); **5 :** Kobdogué (Sedentary); **6 :** Aldjazira2 (Semi-nomadic); **7 :** Aldjazira3 (Semi-nomadic); **8 :** Gourourou (Sedentary); **9 :** Guirkyon (Sedentary); **10 :** Ngonkra1 (Nomadic); **11 :** Ngonkra2 (Nomadic) ; **12 :** Bembaïtada (Sedentary).
