# Supplementary material for: Diversity of trypanosomes in humans and cattle in the HAT foci Mandoul and Maro, Southern Chad—A matter of concern for zoonotic potential?
Source: PLoS Negl Trop Dis. 2021 Jun 9;15(6):e0009323. doi: 10.1371/journal.pntd.0009323 (PMC8224965; doi:10.1371/journal.pntd.0009323)
Supplement: S4 Table — (PDF) [file pntd.0009323.s010.pdf]

S4 Table. Tsetse collection sites, surveys duration, number of traps used and tsetse caught.

**Mandoul_Feb.-17 (4 days)**			**Maro_Feb.-17 (5 days)**			**Maro_Mar.-18 (7 days)**		**Maro_Jun.-18 (7 days)**		**Maro_Dec.-18 (10 days)**			
**Site**	**Traps**	**TN**	**Site**	**Traps**	**TN**	**Traps**	**TN**	**Traps**	**TN**	**Traps**	**TN**	**Total flies**	**Taps**
Donombo3	2	1	Ngakorio	8	0	0	-	-	-	-	-	0	
Konael	2	0	Birya	0	-	24	19	-	-	-	-	19	
Kobiteye	6	0	Kaba/Aldjazira	0	-	8	1	11	3	-	-	4	
Kousserie	2	0	Maïngama/Ridina	0	-	7	0	-	-	-	-	0	
Betoyo	2	0	Kobdogué	4	0	0	-	-	-	-	-	0	
Betel	2	0	Ferme-Taguinan	4	0	0	-	-	-	-	-	0	
Danko	2	0	Baguirgué	14	20	26	36	11	11	18	86	153	
Djarïbé	2	0	Guirkyon	0	-	12	0	-	-	-	-	0	
**Total**	20	1	Total	30	20	77	56	22	14	18	86	**176**	**167**
Average tsetse catch	**0.012**			**0.13**		**0.10**		**0.10**		**0.47**			

(-) Site not visited; TN: Tsetse number; Feb.-17: February 2017; Mar.-18: March 2018; Jun.-18: June 2018; Dec.-18: December2018
