# Supplementary material for: Diversity of trypanosomes in humans and cattle in the HAT foci Mandoul and Maro, Southern Chad—A matter of concern for zoonotic potential?
Source: PLoS Negl Trop Dis. 2021 Jun 9;15(6):e0009323. doi: 10.1371/journal.pntd.0009323 (PMC8224965; doi:10.1371/journal.pntd.0009323)
Supplement: S5 Table — (PDF) [file pntd.0009323.s011.pdf]

S5 Table. Trypanosomes frequency in tsetse fly tissues. Description of single, double and triple infections.

	**Tsetse proboscis**	**Tsetse gut**	**Tsetse Remaining body**
**Single infections**			
T. vivax	42	14	59
T. grayi	0	0	5
T. simiae	0	0	1
T. congolense	1	1	2
***Trypanosoma sp.*-Maro1 (*T. bennetti*-like)**	0	0	1
***Trypanosoma sp.*-Maro2**	0	1	0
**NI100 (not included in calculation)**	12	5	11
**Total**	**43**	**16**	**68**
**Double infections**			
T. godfreyi/T. vivax	0	0	1
T. grayi/T. vivax	5	2	10
T. simiae/T. vivax	2	0	2
T. brucei/T. vivax	2	0	5
T. brucei/T. grayi	0	1	3
T. congolense/T. vivax	2	0	1
T. congolense/T. godfreyi	0	1	0
**Total**	**11**	**4**	**22**
**Triple infections**			
T. brucei/T. grayi/T. vivax	2	0	1
T. congolense/T. brucei/T. vivax	1	0	0
T. brucei/T. grayi/T. godfreyi	0	0	1
***T. grayi/T. godfreyi/*N100**	1	0	0
Trypanosoma sp.*-Maro1 */T. godfreyi/T. vivax	1	0	0
**Total**	**5**	**0**	**2**
**Total PCR positive**	**59**	**20**	**92**
**Total PCR negative**	**112**	**14**	**51**
**Total collected samples**	**171**	**34**	**143**

NI100: Unidentified amplicon (between 100 and 150 bp)
