# Supplementary material for: Diversity of trypanosomes in humans and cattle in the HAT foci Mandoul and Maro, Southern Chad—A matter of concern for zoonotic potential?
Source: PLoS Negl Trop Dis. 2021 Jun 9;15(6):e0009323. doi: 10.1371/journal.pntd.0009323 (PMC8224965; doi:10.1371/journal.pntd.0009323)
Supplement: Attachment — Submitted filename: PNTD-D-20-01868_R1 highlighted edit suggestions.pdf [file pntd.0009323.s017.pdf]

PLOS Neglected Tropical Diseases

Diversity of trypanosomes in humans and cattle in the HAT foci Mandoul and Maro, Southern Chad - A matter of concern for zoonotic potential?

--Manuscript Draft--

**Manuscript Number:**	PNTD-D-20-01868R1
**Full Title:**	Diversity of trypanosomes in humans and cattle in the HAT foci Mandoul and Maro, Southern Chad - A matter of concern for zoonotic potential?
**Short Title:**	Diversity of trypanosomes in humans and cattle-A matter of concern for zoonotic potential?
**Article Type:**	Research Article
**Keywords:**	Trypanosomes; molecular screening; diversity; distribution; Phylogeny; Chad
**Abstract:**	African trypanosomes are parasites mainly transmitted by tsetse flies. They cause trypanosomiasis in humans (HAT) and animals (AAT). In Chad, HAT/AAT are endemic. This study investigates the diversity and distribution of trypanosomes in Mandoul, an isolated area where a tsetse control campaign is ongoing, and Maro, an area bordering the Central African Republic (CAR) where the control had not started.<br>717 humans and 540 cattle blood samples were collected, and 177 tsetse flies were caught. Trypanosomal DNA was detected using PCR targeting internal transcribed spacer 1 (ITS1) and glycosomal glyceraldehyde-3 phosphate dehydrogenase (gGAPDH), followed by amplicon sequencing.<br>Trypanosomal DNA was identified in 14 human samples, 227 cattle samples, and in tsetse. Besides T. b. gambiense , T. congolense was detected in human in Maro. In Mandoul, DNA from an unknown Trypanosoma sp. -129-H was detected in a human with a history of a cured HAT infection and persisting symptoms. In cattle and tsetse samples from Maro, T. godfreyi and T. grayi were detected besides the known animal pathogens, in addition to T. theileri (in cattle) and T. simiae (in tsetse). Furthermore, in Maro, evidence for additional unknown trypanosomes was obtained in tsetse. In contrast, in the Mandoul area, only T. theileri , T. simiae , and T. vivax DNA was identified in cattle. Genetic diversity was most prominent in T. vivax and T. theileri.<br>Tsetse control activities in Mandoul reduced the tsetse population and thus the pathogenic parasites. Nevertheless, T. theileri, T. vivax , and T. simiae are frequent in cattle suggesting transmission by other insect vectors. In contrast, in Maro, transhumance to/from CAR and no tsetse control may have led to the high diversity and frequency of trypanosomes observed including HAT/AAT pathogenic species. Active HAT infections stress the need to enforce monitoring and control campaigns. Additionally, the diverse trypanosome species in humans and cattle indicate the necessity to investigate the infectivity of the unknown trypanosomes regarding their zoonotic potential. Finally, this study should be widened to other trypanosome hosts to capture the whole diversity of circulating trypanosomes.
**Additional Information:**	
**Question**	**Response**
**Financial Disclosure**<br><br>Enter a financial disclosure statement that describes the sources of funding for the work included in this submission. Review the submission guidelines for detailed requirements. View published research articles from *PLOS NTDs* for specific examples.<br><br>This statement is required for submission	This study's financial support came from the Islamic Development Bank (IsDB: https://www.isdb.org/) providing stipend and bench fees to IMAM_36/11210149; 600029774. Deutscher Akademischer Austauschdienst (DAAD: https://www.daad.de/de/), allowing sandwich program stipend to SCHN, CN_A/12/97080). Deutsche Forschungsgemeinschaft (DFG: https://www.dfg.de/), project grants to SK, Ke428/10-1 and Ke428/13-1). The funders had no role in study design, data collection and analysis, decision to publish, or preparation of the manuscript.

Powered by Editorial Manager® and ProduXion Manager® from Aries Systems Corporation

and **will appear in the published article** if the submission is accepted. Please make sure it is accurate.<br><br>**Unfunded studies**<br><br>Enter: *The author(s) received no specific funding for this work.*<br><br>**Funded studies**<br><br>Enter a statement with the following details:<br>• Initials of the authors who received each award<br>• Grant numbers awarded to each author<br>• The full name of each funder<br>• URL of each funder website<br>• Did the sponsors or funders play any role in the study design, data collection and analysis, decision to publish, or preparation of the manuscript?<br>• **NO** - Include this sentence at the end of your statement: *The funders had no role in study design, data collection and analysis, decision to publish, or preparation of the manuscript.*<br>• **YES** - Specify the role(s) played.<br><br>* typeset	
**Competing Interests**<br><br>Use the instructions below to enter a competing interest statement for this submission. On behalf of all authors, disclose any competing interests that could be perceived to bias this work—acknowledging all financial support and any other relevant financial or non-financial competing interests.<br><br>This statement **will appear in the published article** if the submission is accepted. Please make sure it is	The authors have declared that no competing interests exist.

accurate. View published research articles from *PLOS NTDs* for specific examples.<br><br>**NO authors have competing interests**<br><br>Enter: *The authors have declared that no competing interests exist.*<br><br>**Authors with competing interests**<br><br>Enter competing interest details beginning with this statement:<br><br>*I have read the journal's policy and the authors of this manuscript have the following competing interests: [insert competing interests here]*<br><br>* typeset<br><br>This statement is required for submission and **will appear in the published article** if the submission is accepted. Please make sure it is accurate and that any funding sources listed in your Funding Information later in the submission form are also declared in your Financial Disclosure statement.	
**Data Availability**<br><br>Authors are required to make all data underlying the findings described fully available, without restriction, and from the time of publication. PLOS allows rare	Yes - all data are fully available without restriction

Powered by Editorial Manager® and ProduXion Manager® from Aries Systems Corporation

exceptions to address legal and ethical concerns. See the PLOS Data Policy and FAQ for detailed information.<br><br>A Data Availability Statement describing where the data can be found is required at submission. Your answers to this question constitute the Data Availability Statement and **will be published in the article**, if accepted.<br><br>**Important:** Stating 'data available on request from the author' is not sufficient. If your data are only available upon request, select 'No' for the first question and explain your exceptional situation in the text box.<br><br>Do the authors confirm that all data underlying the findings described in their manuscript are fully available without restriction?	
**Describe where the data may be found in full sentences. If you are copying our sample text, replace any instances of XXX with the appropriate details.**<br><br>• If the data are **held or will be held in a public repository**, include URLs, accession numbers or DOIs. If this information will only be available after acceptance, indicate this by ticking the box below. For example: *All XXX files are available from the XXX database (accession number(s) XXX, XXX.)*.<br>• If the data are all contained **within the manuscript and/or Supporting Information files**, enter the following: *All relevant data are within the manuscript and its Supporting Information files.*<br>• If neither of these applies but you are	All relevant data are within the paper and its Supporting Information files. However, only the sequences' references will be added in the manuscript when the manuscript is accepted because they are not deposited yet in the GenBank.

able to provide **details of access elsewhere**, with or without limitations, please do so. For example:<br><br>*Data cannot be shared publicly because of [XXX]. Data are available from the XXX Institutional Data Access / Ethics Committee (contact via XXX) for researchers who meet the criteria for access to confidential data.*<br><br>*The data underlying the results presented in the study are available from (include the name of the third party and contact information or URL).*<br>• This text is appropriate if the data are owned by a third party and authors do not have permission to share the data.<br><br>* typeset	
Additional data availability information:	Tick here if the URLs/accession numbers/DOIs will be available only after acceptance of the manuscript for publication so that we can ensure their inclusion before publication.

Powered by Editorial Manager® and ProduXion Manager® from Aries Systems Corporation

Manuscript

Click here to access/download;Manuscript;Manuscript_Revised.docx

RESEARCH ARTICLE

Diversity of trypanosomes in humans and cattle in the HAT foci Mandoul and Maro, Southern Chad - A matter of concern for zoonotic potential?

Ibrahim Mahamat Alhadj Moussa[1,2] *, Judith Sophie Weber[1,3], Sen Claudine Henriette Ngomtcho[1,4], Djoukzoumka Signaboubo[1], Petra Berger[1], Hassane Mahamat Hassane[5], Sørge Kelm[1]*

1 Centre for Biomolecular Interactions Bremen, Department of Biology and Chemistry, University of Bremen, Bremen, Germany
2 Department of Biology, Faculty of Exacts and Applied sciences, University of N'Djamena, N'Djamena, Chad
3 PANGAEA Data Publisher for Earth and Environmental Science, MARUM, University of Bremen, Bremen, Germany
4 Department of Microbiology, Haematology and Immunology, Faculty of medicine and pharmaceutical Sciences, University of Dschang, Dschang, Cameroon
5 Institut de Recherche en Elevage pour le Développement, N'Djamena, Chad

* **Corresponding authors:** ibrahimmahamat21@gmail.com (IMAM); skelm@uni-bremen.de (SK)

Abstract

Background

African trypanosomes are parasites mainly transmitted by tsetse flies. They cause trypanosomiasis in humans (HAT) and animals (AAT). In Chad, HAT/AAT are endemic. This study investigates the diversity and distribution of trypanosomes in Mandoul, an isolated area where a tsetse control campaign is ongoing, and Maro, an area bordering the Central African Republic (CAR) where the control had not started.

Methods

717 humans and 540 cattle blood samples were collected, and 177 tsetse flies were caught. Trypanosomal DNA was detected using PCR targeting internal transcribed spacer 1 (ITS1) and glycosomal glyceraldehyde-3 phosphate dehydrogenase (*gGAPDH*), followed by amplicon sequencing.

Results

Trypanosomal DNA was identified in 14 human samples, 227 cattle samples, and in tsetse. Besides *T. b. gambiense*, *T. congolense* was detected in human in Maro. In Mandoul, DNA from an unknown *Trypanosoma sp.*-129-H was detected in a human with a history of a cured HAT infection and persisting symptoms. In cattle and tsetse samples from Maro, *T. godfreyi* and *T. grayi* were detected besides the known animal pathogens, in addition to *T. theileri* (in cattle) and *T. simiae* (in tsetse). Furthermore, in Maro, evidence for additional unknown trypanosomes was obtained in tsetse. In contrast, in the Mandoul area, only *T. theileri*, *T. simiae*, and *T. vivax* DNA was identified in cattle. Genetic diversity was most prominent in *T. vivax* and *T. theileri*.

Conclusion

Tsetse control activities in Mandoul reduced the tsetse population and thus the pathogenic parasites. Nevertheless, *T. theileri*, *T. vivax*, and *T. simiae* are frequent in cattle suggesting

transmission by other insect vectors. In contrast, in Maro, transhumance to/from CAR and no tsetse control may have led to the high diversity and frequency of trypanosomes observed including HAT/AAT pathogenic species. Active HAT infections stress the need to enforce monitoring and control campaigns. Additionally, the diverse trypanosome species in humans and cattle indicate the necessity to investigate the infectivity of the unknown trypanosomes regarding their zoonotic potential. Finally, this study should be widened to other trypanosome hosts to capture the whole diversity of circulating trypanosomes.

Keywords: Trypanosomes; molecular screening; diversity; distribution; zoonotic potential; Chad.

Author summary

Sleeping sickness (HAT) is a public health problem in 36 African countries. In Chad, 5 active foci are present in the Southern part. It is caused by trypanosomes, parasites causing disease in humans and livestock. Tsetse flies, the vectors of trypanosomes, declined in the Mandoul focus due to the impact of vector control coupled with active/passive screening and treatment campaigns. In the Maro focus, where such campaigns were absent during these surveys, HAT cases were reported recently. We carried out a study on circulating trypanosomes in humans, cattle and tsetse in these two foci. The results confirmed a reduction of the tsetse population and pathogenic trypanosomes of human and cattle in Mandoul. However, an unknown trypanosome was identified in a human and high frequency of *T. theileri* (known as non-pathogenic) was found in cattle. In contrast, in Maro, a high diversity of trypanosomes was observed, including *T. b. gambiense* and *T. congolense* in humans and several unknown trypanosomes in tsetse. These observations provide evidence of the circulating trypanosomes in the area that recommend widening the investigation to other mammalian hosts and mechanical vectors and considering and monitoring a possible zoonotic potential with the unknown trypanosome and *T. congolense* in humans.

Introduction

Human African Trypanosomiasis (HAT), known as Sleeping Sickness, and Animal African Trypanosomiasis (AAT), known as Nagana, are vector born parasitic diseases of humans and livestock caused by the transmission of extracellular protozoans of the genus *Trypanosoma*. In Central and West Africa, HAT is caused by *T. brucei gambiense*, leading to the chronic form, whereas in East and South Africa, it is caused by *T. b. rhodesiense*, leading to the acute form [1,2]. Millions of people in 36 sub-Saharan African countries are at different levels of risk of infection [3], and WHO had the final goal of sustainable HAT elimination (zero cases) by 2030. AAT occurs in ruminants, camels, equines, swine, and carnivores. The endemic disease severely reduces livestock productivity, and thus also the wealth of livestock farmers and the nutritional well-being of the entire population [4]. The disease is widely distributed across the tsetse-infested belt of the African continent, covering about 10 million km^2. In this belt, approximately 60 million cattle are at risk of infection [5]. Otherwise, this tsetse-infested belt is known to be fertile land, well suited for agriculture and livestock production in Africa [6], [7].

Based on their transmission paths, trypanosomes are divided into Salivaria and Stercoraria. Salivaria are transmitted in the saliva of the vector as it feeds on host blood, whereas Stercoraria are transmitted through vector feces. Among the Salivaria, *T. vivax*, *T. congolense* and *T. b. brucei* are the three most important species pathogenic for livestock and responsible for considerable production losses and morbidity [8]. Three subgenera have been defined in Salivaria trypanosomes: *T. vivax* belongs to the subgenus *Duttonella*; *T. congolense*, *T. simiae* and *T. godfreyi* [9] to *Nannomonas*; and *T. brucei* (with the sub-species *T. b. brucei*, *T. b. gambiense* and *T. b. rhodesiense*), *T. evansi* and *T. equiperdum* to *Trypanozoon* [10,11]. Members of the Stercoraria are the South American *T. cruzi*, the cosmopolitan *T. melophagium* and *T. theileri* [12] and the African *T. grayi* [13]. Among the Stercoraria, *T. theileri* is a parasite of cattle with global distribution, often occurring with high incidence [14].

The main insect vectors of African trypanosomes are tsetse flies of the genus *Glossina* (Glossinidae: Diptera). However, the parasites can also be transmitted mechanically by other biting flies such as tabanids and *Stomoxys* [15], [16]. Other trypanosomes, as *T. theileri*, are mainly transmitted by tabanids.

Chad is part of the trypanosomes endemic zone, with about 65 000 km^2 in the southern part of the country being infested with tsetse flies [17]. However, the extension of the infested area is uncertain due to the lack of reliable recent survey data. In the endemic zone, agriculture activities are extensively practised, and after the rainy season, pastoralists looking for grass and crops residues for their livestock enter the area, often for more than 6 months. The general livestock census carried out in 2015 showed that Chad had more than 93 million cattle, sheep, goats, camels and equines [18]. Like agriculture, the livestock sector is one of the main contributors to the economy of the country [19]. However, AAT has remained a major obstacle to its development, which employs more than 40% of the population [18]. Chad also faces the public health problem HAT. This is currently present in 5 well-known historical HAT foci Moïssala, Tapol, Goré, Mandoul, and Maro [20]. Mandoul and Maro are the most known active foci, and there are still new cases notified [21]. The «Programme National de Lutte contre la Trypanosomiase Humaine Africaine» (PNLTHA) and «l'Institut de Recherche en Élevage pour le Développement» (IRED) with their partners such as FIND, WHO, IRD, LSTM, and PATTEC, are monitoring the disease in Mandoul and most recently in Maro. They are applying tsetse control, and human screenings for *T. b. gambiense* and treatment campaigns to reduce HAT infection risks. This includes usage of Tiny Targets [22], small blue-coloured panels of cloths attracting tsetse, impregnated with insecticide and deployed along river banks where tsetse flies concentrate [23]. The HAT surveillance and tsetse control had started in the Mandoul focus in 2014 and are ongoing. The strategies effectively reduced the tsetse fly populations and the HAT cases [22]. In contrast, in Maro, the campaigns ~~did~~ not ~~yet~~ start when this study was undertaken, and there was a resurgence of new cases.

Animals may harbour the human pathogenic species, serving as a reservoir [24]. On the other hand, infections in humans with animal-pathogenic trypanosomes can occur in rare cases [25]. About 19 cases of atypical human trypanosomes (a-HT) [26], among them *T. b. brucei*, *T. congolense*, *T. vivax*, and *T. evansi* which are considered non-infective to humans, have been reported.

We aimed to investigate the circulating trypanosomes, including the occurrence of potentially zoonotic species in humans and livestock, and in their biological tsetse vector in two active HAT foci, Mandoul and Maro. Mandoul is an area with ongoing HAT surveillances and tsetse control operation, while no such activities were carried out at the time of the surveys in Maro. Taking advantage of the widely used molecular techniques, PCR-based methods targeting trypanosomal internal transcribed spacer I (ITS1) region [27], [28] and glycosomal glyceraldehyde-3-phosphate dehydrogenase (*gGAPDH*) gene combined with sequencing [29], [30], were used to identify trypanosome species in humans and cattle blood samples and tsetse fly tissues. In a time of ongoing tsetse control in Chad to reduce the risk of HAT and AAT infections, the study will contribute to the monitoring strategies, by providing the genetic diversity of circulating trypanosomes, on the way to achieve the goal of diseases elimination.

Methods

Study areas and ethics statements

This study conducted on the distribution of trypanosomes in human, cattle and tsetse flies in Southern Chad was approved in December 2016 by the national bioethics committee under the number 585/PR/PM/MESRI/SEESRI/SG/2016. Detailed protocol and consent documents were submitted to the committee as well as wide information concerning the purpose of the study, provided to the targeted populations. Written consent was obtained from all participants, including those from parents of children under 18 years old.

The Mandoul and Maro HAT foci are located in Southern Chad (Fig 1, S1 Text for details). As for the tsetse fly habitat, Mandoul represents an area where flies are restricted to the swamps formed at the southern limit of the Mandoul river. As the river flows northwards, the swamp deteriorates into a marshy habitat, unsuitable for tsetse. As a result, the population is isolated. Vector control operations with the annual deployment of Tiny Targets ~~had~~ started in 2014 [22]. However, Maro is located in far Southern Chad (Fig 1). Many rivers and their multiple tributaries cross the focus; the most important is the Chari River and its analogous, the Grand Sido, which mark the border with CAR. Tsetse fly habitat is configured by the thin riverine vegetation along the banks of the rivers. Vector control operations ~~had~~ started in the Chadian part in 2018 [21], with annual deployments of Tiny Targets, after conducted most of these surveys. No similar operations have been implemented across the border.

Fig 1. Map showing humans and cattle sampling sites and tsetse trapping spots in the Mandoul and the Maro foci in Southern Chad. Map adapted from A. M. Nour, 2019 [31].

Human surveys

Surveys were conducted in February 2017, March, and June 2018. In each surveyed area, eight randomly selected villages were visited and a military camp included at the request of its inhabitants. In order to proceed with the selection, households were numbered and drawn for participation. A chosen household included all its members automatically. The number of households and participants surveyed per village depended on its population and the individuals who consent for participation. However, we collected no blood from children under five years old.

The open-source Epidemiologic statistics for public health software Version 3.01 (http://www.openepi.com/SampleSize/SSPropor.htm) was used to estimate the human sample size that should be included in the survey. Based on the estimated population recorded from the institutions in charge of HAT control in Chad which were published later, the Mandoul focus includes 114 human settlements with 38-674 inhabitants [22]. In comparison, Maro had 45 settlements with 14-532 inhabitants in 2017 [21]. The present study used these numbers as the total populations from which the sample sizes were calculated. With an accepted margin of error of 5% and 95% confidence interval, the sample sizes required were 381 in the Mandoul and 375 in the Maro.

Cattle surveys

We surveyed the cattle in January, March, June, and November 2018. Sedentary villages, semi-nomadic camps, a nomadic settlement, and a refugee camp were included. Six out of the nine villages selected were the same as those included in the human survey. Representative cattle, from each herd, randomly chosen, were included in this study. Though we strived from random selection, the animals were partly chosen by the herdsmen themselves, presenting animals exhibiting symptoms rather than healthy animals. Similar to that of humans, the open-source Epidemiologic statistics for public health Version 3.01 was used to estimate the sample size of the study. Mandoul has about 14-000 cattle [32], while Maro has over 55-000 [33]. With an accepted margin of error of 5% and 95% confidence interval, the sample sizes required were 374 for Mandoul and 382 for Maro.

The questionnaires were filled during the survey. They addressed the number of animals in the herd, the breeding system, breeds, source of water and nutritional support, health status including symptoms, morbidity and mortality, animal vaccinal status, sex, age of the animals and the herdsmen's education level. Each herdsman answered questionnaires with the support of a local translator on the same day of blood collection.

Human and cattle blood collection and processing

About 5 to 7 mL of blood was collected from the radial vein (venipuncture) of each human participant using vacutainer butterfly needles. 7 to 10 mL was taken from the jugular vein of each animal using a syringe. Collected blood was then directly transferred/connected into a labelled blood collection tube (or vacutainer tube) containing EDTA. The tubes were processed. 200 µL of whole blood were pipetted into 1.5 mL labelled cryotube, and 50 µL were added to 150 µL of Nucleic Acid Preservative Agent, NAPA (25 mM sodium citrate, 10 mM EDTA, 5.3 M ammonium sulfate, pH 7.5), in a separate cryotube.

Tsetse fly collections and processing

Entomological surveys were conducted in February 2017, March, June, and November 2018. Biconical traps were used for this stud[illegible]rapped tsetse flies were dissected as detailed in S2 Text. Proboscises were collected from all flies and stored in 200 µL NAPA. The guts were dissected and kept separately in a 1.5 mL labelled cryotube from live flies [28], [29]. The remaining body of dead tsetse flies (TRB) were kept in 500 µL ethanol after removing the proboscis. Species were morphologically identified and molecularly confirmed by PCR and sequencing, following the procedures described by Shaida *et al.* [34].

DNA extraction and quantification

DNeasy Blood and Tissue Kit (Qiagen, Hilden, Germany) was used to purify DNA from human and cattle blood and tsetse homogenised gut according to the manufacturer instructions with slight modification using 100 µL of treated blood and 100 µL of elution buffer. Photometric quantification of extracted DNA at 260 nm wavelength was performed on a Nanodrop 1000 (Thermo Fisher Scientific, Dreieich, Germany) [28], [29].

DNA from proboscis was extracted using a crude extraction method. The proboscis was incubated at 55°C for 1 h with 55 µL 0.33 mg/mL Proteinase K (Thermo Fisher Scientific, Dreieich, Germany), diluted in 10 mM phosphate buffer, pH 7.4. Heat inactivation of the enzyme followed at 80°C for 45 min [29].

DNA was extracted from the tsetse remaining body (TRB, without proboscis, legs, and wings) using 5% Chelex-100 Resin (BIO-RAD, Hercules, California, USA) [35]. 100 µL was applied on a slightly squashed TRB with a pestle in a 1.5 mL tube. The mixture was incubated at 56°C for 30 min, vortexed and incubated for an additional 5 min at 95°C. The extracted DNA was mixed thoroughly before brief centrifugation at 7000 rpm for 45 s and kept at -80°C.

Molecular amplification and identification of trypanosome species

Nested PCR targeting the ITS1 region of the ribosomal RNA gene locus was carried out to identify the species of trypanosomes. The gene was chosen because of its high copy number and its interspecific length variation, which had previously been used for species identification [27], [35]. For this purpose, established generic and specific primers [27], [28] were used (see S1 Table for details) following previously published procedures [28], [29] adapted to the sample types (blood or tsetse tissues). 25 µL of a master mix containing 2 µM of each outer primer (Sigma-Aldrich, Darmstadt, Germany), 200 µM dNTPs, 2.5 Units Dream*Taq* polymerase, 1x Dream*Taq* buffer (all from Thermo Scientific) and template DNA was used. The volume was 1 µL for human, cattle, TRB, and proboscis template DNA, and 5 µL for tsetse gut tissue template DNA [28], [29]. The cycling conditions for both reactions were as follows: initial denaturation at 95°C for 3 min followed by 30 cycles at 94°C for 60 s, 54°C for 30 s, 72°C for 30 s and final elongation at 72°C for 5 min. Trypanosome species were initially identified based on the size of their ITS1 PCR products, estimated by agarose electrophoresis (for details see S3 Text). Then, the amplicons were purified and subcloned into a linearised pJET 1.2/blunt plasmid using the CloneJET PCR Cloning Kit (Thermo Fisher Scientific) and sequenced by Sanger sequencing following the protocol detailed in S3 Text.

For confirmation of the ITS1 analysis and performing of phylogenetic analyses, a nested PCR targeting the partial *gGAPDH* gene was carried out. *gGAPDH* is a ubiquitous, essential glycolytic enzyme and has a slow rate of molecular evolution making it suitable for studying evolution over large time-scales [30] and therefore, it has been a marker of choice for phylogenetic analysis. A master mix was prepared as described for ITS-1 nested PCR except for the respective primers (S1 Table) [29], [30]. Reaction conditions, for the first PCR, were 95°C for 3 min, followed by 30 cycles at 95°C for 1 min, 55°C for 30 s, and a final extension at 72°C for 10 min. Similar conditions as in the first reaction were used in the second, except the annealing temperature changed to 52°C. PCR products were separated by gel electrophoresis as described above. Amplicons were purified and sequenced as detailed in S3 Text.

Sequences read and phylogenetic tree construction

Sequences were read, analysed and aligned using Geneious Pro 5.5.9 [36]. Alignments of ITS1 and *gGAPDH* sequences were done with Gap open penalty 15 and Gap extension penalty 5. The sequences were aligned against the GenBank database using nucleotide BLAST from NCBI and TritrypDB.

MEGAX software was used to investigate the phylogenetic relationship of trypanosomes based on their *gGAPDH* sequences [37]. The DNA sequences were imported from Geneious and aligned with the MUSCLE algorithm method together with the reference sequences retrieved from the GenBank database. The evolutionary history was inferred using the Neighbour-Joining method. The evolutionary distances were computed with the Maximum Composite Likelihood method [38].

Statistical analysis

The frequencies (in percentage) of trypanosome species in humans, cattle, and tsetse fly samples, were obtained using the Clopper-Pearson binomial test with the lower and upper limits of the 95% confidence interval. Pearson Chi-Square tests were applied to compare, in these sampled animals, trypanosomes frequency to age groups, collection periods and cattle breed, as well as trypanosomes frequency to nomadic, sedentary and refugees' cattle. Student's t-test (unpaired, two-tailed) was used to compare the mean PCV values of recorded healthy and sick cattle on the one hand, and the mean PCV values of trypanosome PCR positive and negative cattle, on the other. In these collected samples, differences were tested for significance at $p<0.05$ using SPSS v.22.0 (IBM, USA). Prism version 7.0a was used for constructing the graphs and Microsoft Excel for managing raw data.

Results

Human survey data

A total of 889 human participants were recruited during the surveys to cover the estimated sample sizes. 409 were from the Mandoul sleeping sickness focus and 480 from the Maro focus. Among those, a total of 717 agreed to give blood samples. 306 samples were collected from the Mandoul focus and 411 from the Maro focus (see S2 Table for details). Recruitment of participants was carried out in 13 sedentary villages (553; 77.13%), 2 semi-nomadic camps (121; 16.87%), 1 nomadic colony (24; 3.35%), and 1 military camp (19; 2.65%), at the rate of 8 settlements in Mandoul and 9 in Maro. 19 to 70 blood samples were collected from each of those villages depending on the size of the population and agreement of the participants. Both genders were represented, 371 males (52%), 340 females (47%) and 6 (1%) with unrecorded sex. Children under 5 years were excluded from blood collection. The main activities practised by the participants are agriculture, livestock, and fisheries.

Trypanosomes identification in humans

Analysis of *Trypanosoma-gGAPDH* gene performed on DNA extracted from human blood samples revealed *T. b. gambiense*, *T. congolense* and *Trypanosoma sp*.-129-H**.** 14 human samples were positive, giving an overall trypanosome frequency of 2.0% (95% CI: 1.1 - 3.3%) in the two foci. Strikingly, in the Maro focus, *T. b. gambiense* DNA was identified in two samples 0.5% (95% CI: 0.1 - 1.7%) and *T. congolense* in 11 leading to an observed frequency of 2.7% (95% CI: 1.3 - 4.7%) (Fig 2). In Maro, the trypanosome's frequency was

3.2% (95% CI: 1.7 - 5.3%) in all surveyed people. In the Mandoul samples, there was no evidence for trypanosomes DNA in humans apart from an unknown trypanosome termed *Trypanosoma sp.*-129-H found in one individual (0.3% (95% CI: 0.0 - 1.8%)). All obtained sequences (see S1 Appendix for details) were aligned against the GenBank database using nucleotide BLAST in NCBI and TritrypDB (see S3 Table for the similarities). *T. congolense* and *T. b. gambi*ense sequences were between 99.4% to 100% similar to described sequences in GenBank, except one *T. congolense* sample which showed only 87% similarity, which was later on confirmed by *T. congolense* specific PCR**.** The sequence of the *Trypanosoma sp*.-129-H was 84% similar to *Trypanosomatidae sp.* LW-2010b (Accession number HQ263665).

Referring to Adams *et al*. [27] and Ngomtcho *et al.* [28] concerning *Trypanosoma* species-specific amplicon sizes, all human samples were screened for *Trypanosoma* DNA targeting ITS1 region and were later on confirmed by sequencing. The analyses revealed 1 positive sample; 0.3% (95% CI: 0.0 - 1.8%) frequency. The sequenced amplicon resulted in 372 base pairs (see S1 Appendix and S1 Fig; *Trypanosoma sp.*-129-H). The sequence showed 97% similarity to *Trypanosomatida sp*., JN673399 previously detected in a hyaena in Tanzania. Interestingly, this is the only sample identified by ITS1-PCR in humans at the Mandoul focus. Furthermore, the presence of trypanosomal DNA was confirmed when targeting *gGAPDH* gene, which was 84% identical to *Trypanosomatidae sp.* HQ263665, detected in *Drosophila obscura*.

Fig 2. Trypanosomes frequency in humans in the Mandoul and Maro HAT/AAT foci. Error bars represent the upper limit of the 95% CI.

Overall, 69% (9) of the cases positive for trypanosomal DNA in the Maro focus were individuals younger than 20 years. Furthermore, most of them (7) were between 10 and 15 years old. This cannot be explained only by a higher percentage of younger participants, as only 42.6% of the participants were under 20 years old, and 14.11% were between 10 and 15 years.

Cattle survey data

A total of 540 cattle blood samples were collected from 93 herds. 462 cattle were from Maro and 78 from Mandoul. The number of cattle sampled in Mandoul is low due to an unexpected anthrax outbreak during the planned survey in the area. Surveys were carried out in 5 sedentary villages, 2 semi-nomadic camps, 1 nomadic settlement, and 1 refugee camp (S4 Table). 61.2% (95% CI: 56.9 - 65.4%) of blood samples were collected from females, and 38.8% (95% CI: 34.6 - 43.1%) from males. Regarding the breeds, 87.6% (95% CI: 84.5 - 90.3%) were Arab zebu, 6.9% (95% CI: 4.9 - 9.4%) White Fulani, and 5.5% (95% CI: 3.7 - 7.9%) were M'bororo breed. 3% of the cattle have had sex and breed unrecorded. The semi-nomadic and nomadic breeders of these surveys owned exclusively Arab zebu breed from whom 275 cattle were sampled, while the sedentary villagers were raising 1, 2 and/or 3 breeds from whom 265 were sampled. 88% (95% CI: 85.5 – 93.1%) of the sedentary groups were breeding cattle for traction and farming work, while 99% (95% CI: 98.8 -100%) of the nomadic and semi-nomadic groups were practising an extensive livestock system. The reason for the transhumance was lack of grasses in the areas during the dry season.

Trypanosomes distribution in cattle using ITS1 nested PCR

ITS1-nested PCR was performed on 540 cattle blood samples. *T. congolense*, *T. brucei ssp.*, *T. simiae*, *T. theileri*, *T. grayi*, *T. godfreyi*, and *T. vivax* were detected (see S1 Fig for amplicon sizes). Similar to a previous report from Cameroon [28], PCR products of about 150 base pairs were observed in 22 samples, but not taken into account for the frequency of trypanosomes. 223 (41.3%; 95% CI: 37.1 - 45.6%) cattle samples were positive for *Trypanosoma* DNA (S4 Table). In the Maro focus, 159 (34.4%; 95% CI: 30.1 - 38.9%) out of 462 cattle contained trypanosomal DNA, whereas in the Mandoul focus 64 (82.1%; 95% CI: 71.7 - 89.8%) out of 78 cattle sampled were positive.

At the Maro focus, *T. congolense* DNA was found most frequently, followed by *T. vivax*, *T. theileri*, *T. brucei ssp.*, *T. grayi*, and *T. godfreyi* (Fig 3A). In contrast, in the Mandoul area (Fig 3B), *T. theileri* was by far most frequently found (91.0% (95% CI: 81.5 - 96.6%) of all positive samples), and only a few animals were positive for *T. vivax* and *T. simiae*. The latter was not detected in Maro.

Mixed infections with two or three different trypanosome species, commonly observed in cattle in previous studies [39], were also found in this study (S4 Table). The most common trypanosome in co-infections was *T. vivax*. *T. congolense* in association with *T. brucei ssp.* were present in 16 cattle, followed by *T. theileri* in co-occurrence with *T. vivax* (5 cases). In 1 cow, the 3 pathogenic species (*T. congolense*, *T. brucei ssp.*, and *T. vivax*) were observed together. For details of mixed infections of other trypanosomes species, see the additional file in S4 Table.

Fig 3. Overall trypanosomes frequency in cattle. A- Maro HAT/AAT focus; B- Mandoul HAT/AAT focus.

Distribution of trypanosomes depending on temporal parameter, migration, age, and cattle breeds of Maro's cattle

Regarding the community structures of Maro's cattle included in the survey, the pathogenic trypanosomes were more frequent (statistically significant, X^2= 11.87; p<0.05) in nomadic than in sedentary cattle (Table 1). Within the nomadic and sedentary cattle groups, *T. vivax* represented 15.6% (95% CI: 11.6 - 20.5%) and 10.3% (95% CI:0.6 - 16.0%), *T. congolense* 15.3% (95% CI: 11.2 - 20.1) and 12.1% (95% CI: 7.6 - 18.1%), and *T. brucei ssp.* 5.5% (95% CI: 3.1 - 8.8%) and 2.4% (95% CI: 0.7 - 0.61%), respectively (Fig 4A). In animals at the refugee camp, these parasites were not found. However, *T. theileri* the worldwide spread bovid trypanosome was identified almost at the same frequency (Fig 4 A) at nomadic, sedentary, and refugees' sites, while *T. grayi* was identified in only few cattle samples of these groups. What stands out is that the cattle of the refugee group were significantly less infected (X^2=7.52; p<0.05) with any trypanosome species than the other sedentary cattle and nomadic group (Table 1). Also, the pathogenic species are more prominent in the nomadic group.

Table 1. Effect of transhumance activities, collection periods, age, breeds, and sex on trypanosomes frequency in Maro.

	Overall trypanosomes			Pathogenic/Non-pathogenic trypanosomes		
	Positive within group N (%)	Overall positive N (%)	X^2, df, p-value	Pathogenic N (%)	Non-Pathogenic N (%)	X^2, df, p-value
**Community, n=462**						

Nomadic	106 (38.5)	159 (34.4)	X^2 = 7.52, df =2, p=0.023	84 (18.2)	22 (4.8)	X^2 = 11.87, df=4, p=0.018
Sedentary	50 (30.3)			38 (8.2)	12 (2.6)	
Refugee	3 (13.6)			0 (0.0)	3 (0.6)	
**Collection period, n=255**						
January	71 (39.7)	101 (39.6)	X^2= 28.46, df=2, p=0.000	60 (23.5)	11 (4.3)	X^2=42.67, df= 4, p=0.000
March	19 (86.4)			11 (4.3)	8 (3.1)	
November	11 (20.4)			10 (3.9)	1 (0.4)	
**Age groups (yrs), n=452**						
< 2.5	24 (24.5)	156 (34.5)	X^2=7.38, df=3, p=0.061	12 (2.6)	12 (2.6)	X^2=17.19, df=6, p=0.009
2.5 to 5	62 (34.1)			49 (10.6)	13 (2.8)	
>5	70 (40.7)			59 (12.8)	11 (2.4)	
**Cattle Breed, n=452**						
Arab Zebu	135 (34.2)	156 (34.5)	X^2=1.16, df=2, p=0.559	106 (23.5)	29 (6.4)	X^2=3.51, df=4, p=0.476
White Fulani	15 (41.7)			11 (2.4)	4 (0.9)	
M'bororo	6 (28.6)			3 (0.7)	3 (0.7)	
**Sex, n=452**						
Male	55 (35.3)	156 (34.6)	X^2=0.32, df=1, p= 0.571	37 (8.2)	18 (4.0)	X^2=4.68, df=2, p= 0.096
Female	101 (64.7)			83 (18.4)	18 (4.0)	
**Health status, n=452**						
Sick	111 (71.2)	156 (34.5)	X^2 =1.14, df=1, p=0.285	91 (20.1)	20 (4.4)	X^2=6.36, df= 2, p= 0.041
Apparently-healthy	45 (28.8)			29 (6.4)	16 (3.5)	

Looking at the seasonal distribution of 255 samples entirely collected from two nomadic settlements in the Maro focus, all identified pathogenic trypanosome species were present either at the beginning of the dry season (November), in the middle (January) or near the end of the dry season (March) (Fig 4B). In November, the frequency of *T. congolense, T. brucei ssp.*, and *T. vivax* were similar. In January, however, there was an increase in the frequency of *T. congolense* (18.4% (95% CI: 13.0 - 24.9%)) and *T. vivax* (15.6% (95% CI: 10.7 - 21.8%)) and a decrease of *T. brucei ssp.* (4.5% (95% CI (1.9 - 8.6%)). In March, while *T. brucei ssp*. frequency from these data was the same as in January, the highest rate of *T. vivax* (36.4% (95% CI:17.2 - 59.3%)) and *T. theileri* (45.5% (95% CI: 24.4 - 67.8%)) were observed. What stands out in the overall samples was in November (Table 1), the cattle were significantly less infected (X^2=28.4; p<0.000) with any trypanosome species than in January, and in March; with the presence of pathogenic species over all the studied period and significant increase of *T. theileri* in March.

Trypanosome-positive cattle were disseminated according to their age groups (Fig 4C). In young cattle (<2.5 years), *T. congolense*, *T. vivax*, and *T. brucei ssp*., the pathogenic species, were at the lowest frequency compared to that in mature group (2.5 to 5 years) and elder cattle (>5 years). The few cases of *T. grayi* and *T. godfreyi* observed were distributed in all age groups. In summary (Table 1), young cattle were significantly less trypanosomal DNA positive (X^2= 7.38; p<0.05) than the mature, and the elder cattle, and this is due to body mass related to age which directly correlated to tsetse attraction [40].

Regarding cattle breed and the presence of *Trypanosoma* DNA (Fig 4D), all the above identified trypanosomes in cattle were found in the Arab zebu breed, as it was the largest group. The same observation was also in the White Fulani breed. Cattle of the M'bororo breed were only positive for *T. congolense* and *T. theileri*. Overall, *Trypanosoma* species DNA (Table 1)

was in 34.2% (95% CI: 29.5 - 39.1%) of Arab zebu, 41.7% (95% CI: 25.5 - 59.6%) of White Fulani, and 28.6% (95% CI: 11.3% - 52.2%) of M'bororo group. And this difference was not statistically significant (X^2= 0.32; p = 0.56).

Fig 4. Distribution of trypanosomes depending on seasonal aspect, migration, age and cattle breeds. n= number of animals included. **A-** Migration-Infection; **B-** Seasonal distribution in 2018; **C-** Age-Infection; **D-** Breed-Infection. Error bars represent the upper limit of the 95% CI.

Packed Cell Volume (PCV) in relation to cattle breed, age and trypanosomes occurrence

The PCV value (%) of 370 animals was recorded in the field. Based on the recorded PCV and the questionnaire answered, 236 potentially sick animals had a mean PCV of 38.2±6.3 while 134 healthy animals averaged at 41.0±6.6, a statistically significant difference (p<0.0001). Overall, correlating with poorer health status, the cattle from the refugee camp had the lowest mean PCV (36.4±7.9) compared with the nomadic cattle (38.1±5.8) and the sedentary animals (41.1±7.1). Regarding the age, young cattle had the lowest PCV mean (37.1±6.8), while the mature cattle with a PCV mean of 40.4±6.5 and the elder with 38.8±6.1. There is no difference between the PCV mean of the M'bororo (40.8±7.0) compared to the White Fulani breeds (41.4±6.3), but the Arab zebu showed a slightly lower PCV mean (38.8±6.5). Within the Arab zebu breed, the distribution of the PCV average correlates to recorded health status (p<0.001). *T. congolense*-infected cattle showed the lowest PCV mean (35.4±5.8) compared with *T. grayi*-infected (39.8±6.7), *T. vivax* (40.2±4.9) and *T. theileri*-infected cattle (clades taken together 40.4±6.4). Mixed infected cattle presented a PCV mean of 36.76±6.0, while all positive cattle taken together had 38.5±6.5 and the negative animals 40.0±6.7. Of 207 physically healthy cattle recorded in total, 112 were found with trypanosomal DNA including typical pathogenic species (mean PCV 39.0±5.6, n=26), while the PCR-negative healthy cattle (41.3±0.7, n=79) had the highest mean PCV. However, there is no statistically significant difference when comparing the PCV of PCR-positive healthy cattle (40.5±0.8, n=55) with PCR-negative healthy cattle (41.3±0.7, n=79).

Tsetse flies survey data

During the first survey in February 2017, 50 traps were set in 8 spots in Mandoul and Maro, respectively. During this survey, only 20 tsetse flies were caught in Maro, and a single tsetse fly in the Mandoul focus (see S5 Table for details). Thereafter, the following surveys were focussed on the Maro area, where 156 additional tsetse flies were caught in 48 traps out of 117 additional traps set, with a highest mean catch of 0.47 tsetse/trap/day in December of 2018 (see S5 Table for details). Of the total of 177 tsetse flies, 98 (54.8%; 95% CI: 47.2 - 62.3%) were females and 79 (45.2%; 95% CI: 37.7 - 52.8%) males. *Glossina fuscipes* group was collected in Mandoul, while *Glossina fuscipes* and *Glossina tachinoides* were trapped in Maro.

During the surveys, the temperature was high (often above 40°C) and the relative humidity low, the tsetse flies died quickly in the cages due to dehydration and other factors such as stress leading to unusually high mortality. Since these dead tsetse flies could not be dissected as planned, after removing proboscis, legs and wings DNA was extracted from the remaining bodies (Tsetse Remaining Bodies, TRB).

Trypanosome identification in tsetse flies

DNA extract from 171 proboscises, 34 guts and 143 TRB were screened for trypanosomal ITS1. 34.5% (95% CI: 27.4 - 42.1%), 58.8% (95% CI: 40.7 - 75.8%), and 63.6% (95% CI: 55.2 - 71.5%) of proboscis, gut and TRB, respectively, contained trypanosomal DNA (S2 Fig A, B and C for details). This frequency combined single and multiple occurrences (see S6 Table for details). *T. vivax*, *T. congolense*, *T. brucei ssp.*, *T. simiae*, *T. godfreyi*, and *T. grayi* a trypanosome identified in reptiles, were identified. One fly (TRB) showed DNA similar to *T. bennetti*, an avian trypanosome [41], termed *Trypanosoma sp.*-Maro1. The sequencing of its *gGAPDH* gene confirmed it. Trypanosomal DNA with some similarity to *Trypanosoma sp.* SDNK92 (ref. LC492122.1) was also identified in one gut and one proboscis from two different tsetse flies; termed *Trypanosoma sp.*-Maro2. The sequences similarities of both these unknown trypanosomes were less than 92% to the referred trypanosomes. Amplicons of about 150 bp and sequences similar to *Bodo caudatus* (203 bp) were also present but were not included in the trypanosomes' frequency. The single tsetse fly caught in the Mandoul focus was positive for *T. vivax*.

The overall trypanosome species distributions found within the positive proboscis, gut, and TRB are shown in Fig 5A, B, and C, with *T. vivax* largely represented in all tissues. Remarkably, all typical livestock pathogenic species were detected in the tsetse fly vector. There was also evidence for DNA of *T. grayi* and *T. godfreyi* in the gut, the proboscis, and in the TRB (Fig 5A, B and C). Additionally, *T. simiae* was also detected, nevertheless only in the proboscises and the TRB.

Fig 5. The overall distribution of trypanosomes in positive tsetse fly samples. A- Proboscis tissues; **B-** Gut tissues; **C-** Tsetse remaining bodies (TRB).

At the Maro focus, 13% (95% CI: 8.3 - 18.7%) of tsetse flies were caught in the Canton Maro, while 86% (95% CI: 79.4 - 90.3%) were from Baguirgué site (Canton Gourourou) (see S5 Table for details). However, looking at the distribution of trypanosomes in these 2 locations at the same collection period (March) in the proboscis tissue, the species distribution was similar in most cases: *T. vivax* (50% of tsetse (n=36) from Baguirgué and 52.6% of flies (n=19) from Birya), *T. congolense* (2.8% in Baguirgué and 5.3% in Birya), similarly for *T. grayi* and *T. simiae*.

Mixed occurrences of trypanosome species DNA were observed in several samples (9.35% in proboscis, 11.76% in the gut and 16.78% in TRB). The most regular was the occurrence of *T. vivax* with one or two other parasites (S6 Table). In the gut tissue, *T. grayi* was identified with *T. vivax* and *T. brucei ssp*. In one proboscis, a presence of all cattle pathogenic species, *i.e. T. congolense*, *T. brucei ssp*., and *T. vivax* was observed.

Summary of the most prominent findings

Overall, *T. b. gambiense*, the pathogenic species of humans and *T. congolense*, the pathogenic species of livestock were found in humans in the Maro sleeping sickness focus (Table 2). In contrast, in the Mandoul focus, only one person showed evidence for trypanosomal DNA. However, it belonged to an unknown *Trypanosoma sp.*-129-H. Regarding the sampled cattle, *T. vivax* was the most frequent trypanosome in Maro while *T. theileri* was found with very

high frequency in Mandoul. *T. vivax* was the most frequent in tsetse flies in both foci including the single tsetse fly trapped in the Mandoul focus. Taken together, the results of this study showed evidence of a higher diversity of *Trypanosoma* species in the Maro area than in the Mandoul focus, including both human and livestock pathogenic species.

Table 2. Overview of trypanosomes frequency (in %) in humans, cattle and tsetse. The lower and upper limits of the 95% confidence interval are indicated in parentheses.

	**Maro**			**Mandoul**		
	**Nr. Collected**	**Trypanosome positive samples**	**Most frequent species**	**Nr. collected**	**Trypanosome positive samples**	**Most frequent species**
**Human**	411	3.2% (1.7 – 5.3)	*T. b. gambiense*<br>*T. congolense*	306	0.3% (0.0 – 1.8)	*Trypanosoma sp.*-129-H
**Cattle**	462	34.4% (30.1 – 38.9)	*T. vivax*<br>*T. congolense*	78	82.1% (71.7 – 89.8)	*T. theileri*
**Tsetse**	176			1		
**Proboscis**	171	34.5% (27.4 – 42.1)	*T. vivax*	1	100%	*T. vivax*
**Gut**	34	58.8% (40.7 – 75.8)	*T. vivax*	1	0%	
**TRB**	143	63.6% (55.2– 71.5)	*T. vivax*	0	0%	

Phylogenetic analysis of trypanosome species

gGAPDH sequences of trypanosomes circulating in the Mandoul and the Maro foci from representative human, cattle and tsetse samples were analysed for phylogenetic relationships and genetic diversity.

Three main clusters were observed when analysing 21 field samples and 8 reference sequences retrieved from GenBank database (Fig 6a). As expected, Salivaria trypanosomes formed one cluster. *T. congolense* and *T. b. gambiense* were closely related and formed a branch while *T. vivax* formed the second branch of this cluster. *T. vivax* showed 2 clades, the East African (*T. vivax* EA) and the African/American (*T. vivax* A/A, also called West African/South American type WA/SA). Similarly, known Stercoraria trypanosomes formed the second cluster including two branches: *T. theileri* and *T. bennetti*. The sequence of *Trypanosoma sp.*-Maro1 was closely related to *T. bennetti* (FJ649486, 92,6% similarity).

Interestingly, a third cluster was formed with the outgroup reference, and this concerned *Trypanosoma sp.*-129-H having 84.1% similarity to *Trypanosomatidae sp.* LW-2010b Dobs; HQ263665. This *Trypanosomatidae sp.* was previously found in a fly *Drosophila obscura*, (using trypanosome-*gGAPDH*) and *Hyena* (targeting trypanosomal ITS1).

T. theileri was widely distributed and very diverse (Fig 6b). Two main clades, clade IA and IB on the one side, and clade IIA and IIB on the other side were observed as previously described [28], [39], [42]. Interestingly, besides the sub-clades IIA and IIB, one other sub-clade was observed and thus the sequences were 99.8% similar to *T. theileri* sequences from the GenBank (references MK674002 and HF545654). Additionally, some sequences were closely related to clade IA; however, they formed a different sub-clade.

Fig 6. Neighbour-Joining trees based on alignments of *gGAPDH* sequences from trypanosome species detected in human, cattle, and tsetse in Southern Chad. They were calculated using complete gap deletion and tested with 700 bootstrap replications using MEGA X software (Kumar et al., 2018). **a-** *gGAPDH* nucleotide sequences of 21 representatives of different trypanosome species and 8 reference sequences retrieved from GenBank were aligned. **b-** *gGAPDH* nucleotide sequences of 15 representatives of *T. theileri* clades detected only in cattle samples and 8 reference sequences belonging to IA, IB, IIA, IIB and U29 *T. theileri* clades retrieved from GenBank were aligned. Evolutionary analyses involved 613 bp (**a**) and 563 bp (**b**) stretches. Abbreviations: EA, East Africa; A/A: Africa/America.

Discussion

Different trypanosome species (See S1 Fig for details), including typical pathogenic and non-pathogenic species belonging to the Stercoraria and Salivaria trypanosomes, were identified during this study. The evidence of high trypanosome frequency in tsetse flies (see S6 Table for details) vigorously supports our observation of high frequency in cattle and humans in Maro (Table 2). This suggests that tsetse flies are transmitting cattle and human infective trypanosomes in this focus, whereas in Mandoul, the low occurrence of tsetse flies correspond to a lower diversity and a different species pattern. It has to be kept in mind that our molecular approach does not confirm active parasite infections, because traces of DNA ~~as~~ remains from previously present parasites are detected by this sensitive method. It should also be noted that the selection of animals presented for sampling by the herdsmen may have been biased due to their interest in presenting sick animals for possible treatment. And this was especially perceived when surveying the nomadic groups.

The noticeable relevant findings were the presence of *T. b. gambiense* DNA in humans in Maro, stressing the ongoing HAT infection risk of the area, besides unexpected *T. congolense* in human samples. In the Mandoul focus, there was no evidence for such species neither in cattle nor in humans. However, there was evidence of an unknown trypanosome, *Trypanosoma sp.*-129-H in one man cured of HAT, but with prevailing symptoms.

With regards to the overall parasites diversity, only very few *T. vivax* and *T. simiae*, and a high rate of *T. theileri* were detected in cattle in Mandoul. These species are known to be transmitted independently from the tsetse fly, which corresponds to the very low number of fly catches during our entomological survey. In Maro, tsetse flies were abundant, and HAT/AAT pathogenic species were present in a relatively high number of tsetse flies, in addition to previously undescribed trypanosomes, *Trypanosoma sp.*-Maro1 (*T. bennetti*-like) and *Trypanosoma sp.*-Maro2. The findings indicate differences between the two foci in terms of trypanosomes diversity potentially in relation to tsetse fly's abundance.

Situation in humans

Regular screening campaigns of humans for HAT cases have been undertaken by the Ministry of public health and its partners within the historical HAT-foci in Southern Chad. The case definition is based on CATTs test > 1/8 and to some extent on LAMP assays and microscopy in the Mandoul focus. Whereas HAT cases have been decreasing over the years in the Mandoul focus [22~~]~~, ~~[~~43], recently, the resurgence of 23 new cases was reported at the old Maro focus [44]. In this study, we identified *T. b. gambiense* in two human samples from the Maro area. It was detected in one child and one older man confirming the presence of the parasite in

the area and the ongoing risk of HAT infections, as it was also identified in animal reservoirs reported by Vourchakbé *et al.*, 2020 [45]. These two participants have had no previous infection with this parasite. However, none of the samples collected in Mandoul was interestingly, *T. b. gambiense* positive, which could be due to the reduction of its incidence reported by Mallaye *et al.*, [21] and the low tsetse fly number. Thus, a wider monitoring is needed to state more precisely the overall parasite prevalence in the two foci.

An interesting observation was the presence of an older participant in the survey with a cured HAT infection. He still had the repercussions of HAT-like symptoms, when the survey was undertaken but had tested negative for *T. b. gambiense* in all the last active screening campaigns according to the health service of the locality. Unexpectedly, both PCR targeting *Kinetoplastida* ITS1 region and *gGAPDH*, evidence was obtained for the presence of an undescribed trypanosome. The sequences of *Trypanosoma sp.*-129-H DNA were related to unidentified *Trypanosomatida sp.* found in *Hyena* (using ITS-1, JN673399) and in *Drosophila obscura* (using *gGAPDH*, HQ263665). As the techniques used to diagnose HAT recommended by WHO are very specific for *T. b. gambiense* [2] and sensitivity of microscopy analysis is limited, *e.g.* in case of low parasitemia, this parasite may have remained undetected for a long time. Thus, immediate further investigations would need to be undertaken to isolate and characterise the parasite and furthermore look closely on the pathogenicity of this unknown trypanosome, in case of non-transient infection.

A second alarming observation was the presence of *T. congolense* DNA in human blood samples in Mar[illegible]The abundant presence of *T. congolense* DNA we observed in cattle (Fig 3) indicated that also humans in this area are highly exposed to bites by tsetse flies transmitting *T. congolense* (which flies as well have been identified with its DNA traces) (Fig 5). This becomes evident, when looking at single settlements, for example, one of the nomadic communities, where one human, 28 cattle (16%) and 3 tsetse flies (13%) were *T. congolense* positive. Usually, humans are resistant to *T. congolense* due to innate protection, including most trypanosome species [46]. Among the innate protection, the trypanolytic factors (TLF1 and TLF2) [47] found in human serum are able to lyse trypanosomes upon entry [48], therefore serving as a natural host innate parasite defence mechanism. However, several cases of atypical HAT have been reported [26],[49], suggesting that *T. congolense* might be able to produce infections in man. Therefore, other investigations need to be undertaken on the infected humans to determine and discuss its pathogenicity and zoonotic potential. It is quite interesting that also other *Trypanosoma* species were circulating in the Maro area, however they were not detected in humans, as *e.g. T. vivax*.

Situation in cattle

Apart from the direct risk of HAT for human health, AAT denotes a heavy burden on the livestock and agriculture-based livelihoods of the people in rural areas. This implies, *e.g.*, the constant use of drugs to treat the animals; which is not without cost and contributes negatively to the economic development of the affected rural areas [2],[50]. The animal-pathogenic species affecting cattle mainly include *T. congolense*, *T. vivax*, and, *T. b. brucei*.

As indicated above, the abundant presence of *T. congolense* in cattle in Maro (Fig 3) poses a high risk of AAT in this area. Continuous surveying is therefore needed to treat the respective

animals. A promising finding was the absence of *T. congolense* in the Mandoul area, indicating that the tsetse control campaign effectively reduced *T. congolense*-induced AAT. However, due to the low number of cattle sampled in Mandoul, the obtained results cannot be extrapolated to the whole area.
The picture looks different for the presence of *T. vivax*. Though found in fewer of the tested cattle, *T. vivax* was detected in Mandoul as well as the Maro focus. This can be connected to the ability of *T. vivax* to be transmitted mechanically and stresses the need for additional control measures, apart from tsetse fly control, when targeting this parasite.

Looking in more detail on the intraspecies diversity, *T. vivax* clustered in two clades. Both were present in samples collected from the Maro focus. One of these clades groups with the East African *T. vivax* (EA) with a strong homology and high similarity. This clade EA was described for the first time in Tanzania [51], then in Nigeria [29] and in Cameroon [39] in 2019, and now its presence in Chad was confirmed in this study. This leads to the suggestion that either this strain is spreading across the African continent (Central and Western Africa) or increased sequencing data are revealing a more detailed picture on the parasite diversity, which could not have been observed before, as also reported by Adams *et al.*, [52] on performance of molecular identification techniques. The second widespread clade, *T. vivax* A/A (African/American or WA/SA) [51], was present in samples from both foci.

T. vivax pathogenicity in cattle appears to be isolate-dependent. *T. vivax* A/A were described to be more pathogenic than EA isolates [53],[10]. Furthermore, a strain-/subgroup-dependent pathogenicity level has been observed in the same geographical region depending on the infective species of the tsetse fly [52],[53]. In agreement with the pathogenicity related to *T. vivax* subgroup, 3 cattle in the Mandoul focus died a few weeks after our survey, and the obtained sequences clustered with the *T. vivax* A/A clade. Already during sampling, these animals showed AAT-symptoms such as inappetence, asthenia, tearing, weight loss and oedema. This suggests that this strain might be responsible for AAT outbreaks in the area and possibly throughout the country, which might put the effort of fighting animal diseases under duress.

Several other trypanosomes were present in cattle samples, including *T. theileri*, a worldwide distributed parasite generally considered non-pathogenic. Sequence analysis of *T. theileri gGAPDH* sequences revealed several sub-groups, among them the four known clades IA, IB, IIA, and IIB [39]. In this study, another clade (235-260-B-BC and 235-253-B-BC) closely related to clade IA was observed as well as a clade formed by the new strain found in Uganda (*T. theileri* Uganda29; HF545654) and in Cameroon (*T. theileri* clone 81; MK674002) [39]. This confirms that *T. theileri* is genetically diverse. Despite their presence in cattle, *T. theileri* was not observed in the tsetse samples which indicate its transmission to be independent from tsetse flies. It should be noted that this parasite was detected abundantly in Mandoul' cattle, where tsetse flies are close to ~~annihilation.~~ Here, its frequency was much higher (82.1%; 95% CI: 71.7 - 89.8%), also higher than that obtained in previous studies; *e.g.* in Uganda (47%) [54] and in Northern Cameroon (30.5%) [39].

The genetic variation within *T. theileri* might lead to strain-dependent implications on the health status of the animals. Along this line, reports from Cameroon observed that cattle infected with clade IIB have lower PCVs than those infected with clade IA or IB [28], [39]. In

this study from Chad, cattle presenting *T. theileri* clade IA, have slightly low PCVs (34.33 ± 6.4) similar to those infected with *T. congolense* (35.48 ± 5.8), lower than those having *T. theileri* clade IIB (37.67 ± 8.5), *T. grayi* (39.8 ± 6.7) or cattle negative for trypanosomal DNA (40.03 ± 6.7). However, this outcome is not sufficient to speculate on the pathogenicity of these parasites (*T. theileri* and *T. grayi*) as many other parameters can change the PCV value. Nevertheless, already previous studies pointed out isolated cases of *T. theileri* pathogenicity [55], [56], including a most recent cases in Italy [57]. These observations and the PCVs value linked to clades could give a path for research to determine whether *T. theileri* can be a pathogenic trypanosome under certain circumstances or when present in a certain genetic variant.

Further parasites were detected in cattle in Maro. Interestingly, evidence for *T. grayi* was obtained in 10 cattle blood samples. At first sight, this was unpredicted, since when describing this parasite, Hoare *et al.,* [58] could not infect mammals with this reptilian parasite. However, more recently, also in Cameroon *T. grayi* was detected in cattle [28], [39]. Together, these observations support the note that there might be a burning issue of host adaptation of this trypanosome and change in its lifecycle. Thus, it is important to look more closely for *T. grayi* infections in cattle and its possible pathogenicity in this host.

Tsetse fly data

Overall, a high diversity of trypanosomes was identified in tsetse, mirroring the diversity observed in humans and cattle in Maro.

The frequency of *T. vivax* found in tsetse flies was higher compared to other studies across the West and Central African tsetse fly area, where the highest was 34% overall positive [28], and 11.7% in all screened proboscises [29]. The presences in the gut tissues is likely to be remains of a recent blood meal since *T. vivax* is not expected to colonise the tsetse gut [59].

The sites of the development of trypanosomes during their life cycles in tsetse is species-specific [10], [60]. However, already in previous studies, molecular identification of trypanosomes revealed unexpected sites of their DNA, for example, similar to our findings *T. grayi* in the proboscis [28], [29] or *T. vivax* in the midgut [61]. This could be explained on the one hand by the sensitivity of the method, as it detects down to 10 pg DNA when field conditions were mimicked using tsetse fly midgut [27], and thus, during the transit of the parasites. On the other hand, it could be explained by residual DNA from a recent blood meal.

Described *T. bennetti* was initially found in the American Kestrel (*Flaco saoverius*) [62] and in European passerine birds and raptors confirmed by its isolation from nestlings and yearlings, suggesting its local transmission [63]. Similar sequences but not identical to this species was identified in one tsetse; *Trypanosoma sp.*-Maro 1. Interestingly, when performing blood meal analysis on this tsetse fly sample, a bird (*Ardea purpurea*) was revealed as its blood meal source. This is a piece of evidence that it is most likely to be a bird parasite.

As stated above, tsetse control activities were ongoing in Mandoul for the last 3 years and during our surveys. It is important to note that only one tsetse fly was caught in the Mandoul focus (which is a more confine area) during the surveys, although 20 traps were set at 8 different locations within 3 days. The rarity of the tsetse vector is most likely due to the success of

tsetse control organised by IRED and its Partners by setting impregnated Tiny Targets that attract and kill tsetse. Also, Mahamat *et al.*, [22] observed a similar frequency with only 5 tsetse flies being caught during nine surveys at this area in 2017. Similar observations of the Tiny Targets effect on tsetse population reduction were reported in West and East Africa [64], [65]. In contrast, in the Maro focus, the tsetse control has just started in 2018, after we collected most of the samples of this study. This probably played a role in the high trypanosome diversity observed in the area (see S3, S4, and S6 Tables) as also more tsetse flies were found.

Differences between the two areas and concluding remarks

Looking at the global picture of trypanosome distribution, a different pattern emerged in the two foci. Overall, Maro showed high diversity in tsetse-transmitted parasites. In Mandoul, diversity was much lower. On the one hand, this might be due to the reduced number of cattle samples tested in Mandoul. On the other hand, the parasites present in the blood of the surveyed animals shifted from animal pathogenic and tsetse transmitted parasites in Maro to generally considered non-pathogenic parasites (*T. theileri*) and parasites that do not only rely on tsetse flies for transmission in Mandoul (*T. vivax, T. simiae*). A higher rate of positive cattle accompanies this observation (Table 2). A similar pattern was observed by Paguem *et al.*, [39]. This is quite interesting, because it evokes some kind of competition: (1) Either the blood-sucking insects are competing and the reduction of one species, in this case, tsetse fly, benefits the growth of other blood-sucking flies such as Tabanids or *Stomoxys*. The presence of the *Tabanidae* and *Stomoxys* in the Mandoul area was previously reported [50]; however, their implication on the transmission of the parasites in the area was not studied yet, which urge to be undertaken. (2) Or the trypanosomes are competing for the same hosts, and *T. theileri* might only be able to establish infections when the immune system is not activated against trypanosomes by pathogenic trypanosome species.

The identification of *Trypanosoma sp*.-129-H and *T. theileri* in Mandoul suggests that other trypanosome parasites are taking place and might be transmitted by other biting arthropods known as mechanical vectors.

It will be interesting to follow up, whether also in the Maro focus, the tsetse populations will also be reduced successfully, and see which impact this will have on the diversity of trypanosomes in this area. However, Maro is bordering the CAR and transhumance activities to and from areas close to its National Park (Bamingui-Bangouran) in search of animal food supply, are frequent. This could impact in this region, as observed in the trypanosome's diversity that emerged from this study. The CAR savannah areas, known for their densely populated parts, are heavily infested with tsetse flies and potentially under the continuing threat of an AAT epidemic [21]. Unfortunately, due to the volatile and complex situation, no tsetse fly control is underway in the CAR, which area could be associated in a joined effort. Thus, the Maro focus should get great more attention, and more host species and individuals should be monitored.

Several other patterns could be observed in this study. The proportion of *T. vivax* at the beginning of the dry season (November) shifting to an increase towards the end of the dry season (March) (Fig 4B) is in agreement with a report from Nigeria [66], which stated its predominance in the dry season (and that of *T. congolense* in the wet season). This could be explained by the presence of other biting insects, throughout the season acting as mechanical vectors

and maintaining the bovine trypanosomosis in the herd while tsetse populations are suppressed. Regarding trypanosomes distribution between cattle breeds, White Fulani group presented the highest frequency of *T. congolense*, *T. brucei ssp.*, and *T. godfreyi*, while *T. theileri* was widely predominant in M'bororo group and *T. vivax* in Arab zebu (Fig 4D). Our observation corroborates with that of Odeniran *et al.*, [66] who reported a high frequency of trypanosomes in White Fulani farms in Nigeria. This is due to the transhumance activity. Looking at this, results of nomadic animals included in this study (Fig 4A) reflect the observation with a high frequency of *T. vivax* followed by *T. congolense* and *T. brucei ssp.* This explains that transhumance activity would impact the transmission cycle and persistence of the parasites in the area [66], besides the susceptibility of the breeds [67] dependent on *Trypanosoma* species. However, for a conclusive picture, the seasonal impact of transhumance activities, and cattle breeds susceptibility need more longitudinal data addressing this issue.

Conclusion

WHO had the goal to eliminate ~~the~~ HAT as a public health problem by 2020, and the final goal of the sustainable disease elimination by 2030. In order to achieve this goal in Chad, the National Program with its partners have been organising campaigns; active screening and treatment of humans, as well as tsetse fly control by setting impregnated Tiny Targets. This strategy contributed to the reduction of tsetse populations and known pathogenic trypanosomes in the Mandoul area, as observed in the data emerging from this study. However, there is evidence for *T. theileri*, *T. vivax*, *T. simiae* in cattle and an unknown trypanosome in human which could lead to a resurgence and probable pathogenicity of the disease complex in the Mandoul area, and other vectors could play a role. Thus, the situation needs to be monitored. In contrast, the Maro area bordering the CAR could be an unknown reservoir of parasites. Based on its proximity with the CAR, which is having a complex and volatile social situation as well as the uncontrolled crossing of the borders of pastoralists and their livestock, it is highly expected that tsetse flies, others biting insects and various trypanosome species can move from one country to the other. As an outcome of this study, high diversity and frequency of trypanosomes have been observed in human, cattle, and tsetse fly vector including typical and atypical pathogenic species, suggesting a zoonotic potential leading to get close attention in this area. Therefore, to achieve the goal of eliminating HAT as a public health problem, all the players such as natural host and vector, and reservoirs and mechanical vectors have to be considered to exclude a resurgence of typical HAT from these sources and neighbouring areas, and atypical trypanosomiasis in case of non-transient infection.

Supporting information Captions

S1 Appendix. Sequences of trypanosome species identified in this study.
(PDF)

S1 Fig. Gel picture showing the amplicon sizes of trypanosomal ITS-1.
(PDF)

S1 Table. Generic and specific primers used in the study.
(PDF)

S1 Text. Study areas description.
(PDF)

S2 Fig. Percentage of positive and negative tsetse tissues for *Trypanosoma* sp. DNA.

(PDF)

S2 Table. Target villages for human blood samples collection.
(PDF)

S2 Text. Samples collections and processing, and DNA extraction and quantification.
(PDF)

S3 Table. Sequence similarities of trypanosomes detected in humans with those retrieved from the GenBank.
(PDF)

S3 Text. Molecular amplification and identification procedure, and subcloning and sequencing of amplicons.
(PDF)

S4 Table. Trypanosomes frequency in cattle and cattle sampled per village.
(PDF)

S5 Table. Tsetse collection sites, surveys duration, number of traps used and tsetse caught.
(PDF)

S6 Table. Trypanosomes frequency in tsetse fly tissues.
(PDF)

Acknowledgements

We would like to thank the "Institut de Recherche en Élevage pour le Développement", and the "Programme National de Lutte contre la Trypanosomiase Humaine Africaine" (PNLTHA) in Chad for their collaboration and field survey supports at the beginning of this study, in particular Severin Mbainda, Brahim Guihini Molo, Richard Ouang and Nadjitessem Tanassingar as respectively nurse, entomologist, laboratory technician and veterinarian doctor and Peka Mallaye, the coordinator of PNLTHA. We are grateful to the administrative authorities and traditional leaders, especially the "Chef de Canton" of Bembaïtada, Maro, and Gourourou, without their support, samples could not have been collected. Special thanks go to Sister Cecilia, Sister Titi, and their team for their services during our stay in the monastery. Special thanks are extended to AG Kelm members at the University of Bremen particularly to Frank Dietz, Mario Wespey, Archille Paguem, Jana Rosenau, Petra Seekamp and Nazila Isakovic for fruitful discussions and technical assistance and Sabine Limberg for the administrative support.

Author contributions

Conceived and designed the experiments: IMAM, SK, HMH, SCHN, PB. Performed the experiments: IMAM, SK, PB, DS, HMH. Analysed the data: IMAM, JW. Contributed reagents/material/analysis tools: SK, IMAM, SCHN, PB, JW, HMH. Wrote the paper: IMAM, SK, JW, HMH, PB, DS.

References

[1] D. Malvy and F. Chappuis, "Sleeping sickness," *Clinical Microbiology and Infection*, vol. 17, no. 7, pp. 986–995, 2011, doi: 10.1111/j.1469-0691.2011.03536.x.

[2] WHO, "WHO_Trypanosomiasis, human African (sleeping sickness), Feb. 2020_," *https://www.who.int/news-room/fact-sheets/detail/trypanosomiasis-human-african-(sleeping-sickness),* 2020.

[3] "WHO | Progress on eliminating sleeping sickness as a public health problem," *WHO*. http://www.who.int/trypanosomiasis_african/news/progress-on-eliminating-hat-as-public-health-problem/en/ (accessed Dec. 26, 2019).

[4] H. R. Holt, R. Selby, C. Mumba, G. B. Napier, and J. Guitian, “Assessment of animal African trypanosomiasis (AAT) vulnerability in cattle-owning communities of sub-Saharan Africa,” *Parasites & Vectors*, vol. 9, no. 1, Dec. 2016, doi: 10.1186/s13071-016-1336-5.

[5] G. Cecchi, M. Paone, U. Feldmann, M. J. Vreysen, O. Diall, and R. C. Mattioli, “Assembling a geospatial database of tsetse-transmitted animal trypanosomosis for Africa,” *Parasites & Vectors*, vol. 7, no. 1, p. 39, 2014, doi: 10.1186/1756-3305-7-39.

[6] International Livestock Centre for Africa, International Laboratory for Research on Animal Diseases, and African Trypanotolerant Livestock Network, Eds., *Livestock production in tsetse affected areas of Africa: proceedings of a meeting held in Nairobi, Kenya from the 23rd to 27th November 1987*. Nairobi, Kenya: ILCA/ILRAD, 1988.

[7] M. J. B. Vreysen, “PRINCIPLES OF AREA-WIDE INTEGRATED TSETSE FLY CONTROL USING THE STERILE INSECT TECHNIQUE,” p. 16, 2001.

[8] D. Steverding, “The history of African trypanosomiasis,” *Parasites & Vectors*, vol. 1, no. 1, p. 3, 2008, doi: 10.1186/1756-3305-1-3.

[9] D. K. Masiga, J. J. McNamara, C. Laveissière, P. Truc, and W. C. Gibson, “A high prevalence of mixed trypanosome infections in tsetse flies in Sinfra, Côte d’Ivoire, detected by DNA amplification,” *Parasitology*, vol. 112, no. 1, pp. 75–80, Jan. 1996, doi: 10.1017/S0031182000065094.

[10] C. A. Hoare, “The trypanosomes of mammals. A zoological monograph.,” *The trypanosomes of mammals. A zoological monograph.*, 1972, Accessed: Dec. 26, 2019. [Online]. Available: https://www.cabdirect.org/cabdirect/abstract/19722902365.

[11] N. D. Levine *et al.*, “A Newly Revised Classification of the Protozoa*,” *The Journal of Protozoology*, vol. 27, no. 1, pp. 37–58, 1980, doi: 10.1111/j.1550-7408.1980.tb04228.x.

[12] N. Yokoyama *et al.*, “Genetic diversity in Trypanosoma theileri from Sri Lankan cattle and water buffaloes,” *Veterinary Parasitology*, vol. 207, no. 3–4, pp. 335–341, Jan. 2015, doi: 10.1016/j.vetpar.2014.12.006.

[13] C. A. Hoare, “II] Systematic description of the mammalian trypanosomes of Africa.,” *II] Systematic description of the mammalian trypanosomes of Africa.*, pp. 34–59, 1970, Accessed: May 28, 2020. [Online]. Available: https://www.cabdirect.org/cabdirect/abstract/19712206879.

[14] Y.-F. Lee *et al.*, “Evidence of intracellular stages in Trypanosoma (Megatrypanum) theileri in non-phagocytic mammalian cells,” *Veterinary Parasitology*, vol. 191, no. 3–4, pp. 228–239, Jan. 2013, doi: 10.1016/j.vetpar.2012.08.027.

[15] F. Baldacchino, V. Muenworn, M. Desquesnes, F. Desoli, T. Charoenviriyaphap, and G. Duvallet, “Transmission of pathogens by Stomoxys flies (Diptera, Muscidae): a review,” *Parasite*, vol. 20, 2013, doi: 10.1051/parasite/2013026.

[16] A. G. D. Ndalembouly *et al.*, “Contribution à l’étude des Stomoxes et Tabanides, vecteurs mécaniques des trypanosomoses, dans la région de Ndendé au sud du Gabon,” p. 13, 2016.

[17] N. Ndeledje *et al.*, “Treating Cattle to Protect People? Impact of Footbath Insecticide Treatment on Tsetse Density in Chad,” *PLoS ONE*, vol. 8, no. 6, p. e67580, Jun. 2013, doi: 10.1371/journal.pone.0067580.

[18] R E C E N S E M E N T G E N E R A L D E L ’ E L E V A G E and (R G E), “PRINCIPAUX RESULTATS DEFINITIFS.” 2015, [Online]. Available: http://praps-tchad.net/im/Recensement.pdf.

[19] PANA, “Programme d’Action National d’Adaptation (PANA) aux Changements Climatiques,” 2009, [Online]. Available: http://extwprlegs1.fao.org/docs/pdf/cha146837.pdf.

[20] WHO, “Report of a WHO meeting on elimination of African trypanosomiasis (Trypanosoma brucei gambiense).” 2012, [Online]. Available: https://apps.who.int/iris/bitstream/handle/10665/79689/WHQ?sequence=1.

[21] ISCTRC and PATTEC, “35TH GENERAL CONFERENCE OF THE INTERNATIONAL SCIENTIFIC COUNCIL FOR TRYPANOSOMIASIS RESEARCH AND CONTROL (ISCTRC) AND 18TH PATTEC NATIONAL COORDINATORS MEETING.” 2019, [Online]. Available: https://labex-parafrap.fr/images/documents/isctrc_35th_programme_book_abstracts_final_en.pdf.

[22] M. H. Mahamat *et al.*, “Adding tsetse control to medical activities contributes to decreasing transmission of sleeping sickness in the Mandoul focus (Chad),” *PLOS Neglected Tropical Diseases*, vol. 11, no. 7, p. e0005792, Jul. 2017, doi: 10.1371/journal.pntd.0005792.

[23] “Targeting Tsetse,” *LSTM*. https://www.lstmed.ac.uk/research/departments/vector-biology/vector-control/targeting-tsetse (accessed May 20, 2020).

[24] S. Funk, H. Nishiura, H. Heesterbeek, W. J. Edmunds, and F. Checchi, “Identifying Transmission Cycles at the Human-Animal Interface: The Role of Animal Reservoirs in Maintaining Gambiense Human African Trypanosomiasis,” *PLoS Computational Biology*, vol. 9, no. 1, p. e1002855, Jan. 2013, doi: 10.1371/journal.pcbi.1002855.

[25] WHO, “Animal Trypanosome in Human(Atypic infection?).” 2020, [Online]. Available: https://www.who.int/trypanosomiasis_african/disease/parasite/en/.

[26] P. Truc *et al.*, “Atypical Human Infections by Animal Trypanosomes,” *PLoS Neglected Tropical Diseases*, vol. 7, no. 9, p. e2256, Sep. 2013, doi: 10.1371/journal.pntd.0002256.

[27] E. R. Adams, I. I. Malele, A. R. Msangi, and W. C. Gibson, “Trypanosome identification in wild tsetse populations in Tanzania using generic primers to amplify the ribosomal RNA ITS-1 region,” *Acta Tropica*, vol. 100, no. 1–2, pp. 103–109, Nov. 2006, doi: 10.1016/j.actatropica.2006.10.002.

[28] S. C. H. Ngomtcho, J. S. Weber, E. Ngo Bum, T. T. Gbem, S. Kelm, and M. D. Achukwi, “Molecular screening of tsetse flies and cattle reveal different Trypanosoma species including T. grayi and T. theileri in northern Cameroon,” *Parasites & Vectors*, vol. 10, no. 1, Dec. 2017, doi: 10.1186/s13071-017-2540-7.

[29] J. S. Weber *et al.*, “Genetic diversity of trypanosome species in tsetse flies (Glossina spp.) in Nigeria,” *Parasites & Vectors*, vol. 12, no. 1, Dec. 2019, doi: 10.1186/s13071-019-3718-y.

[30] P. B. Hamilton, J. R. Stevens, M. W. Gaunt, J. Gidley, and W. C. Gibson, “Trypanosomes are monophyletic: evidence from genes for glyceraldehyde phosphate dehydrogenase and small subunit ribosomal RNA,” *International Journal for Parasitology*, vol. 34, no. 12, pp. 1393–1404, Nov. 2004, doi: 10.1016/j.ijpara.2004.08.011.

[31] A. M. Nour, “Fonctionnement hydrologique, chimique et isotopique du principal affluent du lac Tchad : le système Chari-Logone,” p. 183, 2019.

[32] J.-B. Rayaisse *et al.*, “Delivering ‘tiny targets’ in a remote region of southern Chad: a cost analysis of tsetse control in the Mandoul sleeping sickness focus,” *Parasites & Vectors*, vol. 13, no. 1, Dec. 2020, doi: 10.1186/s13071-020-04286-w.

[33] DASHBOARD #2, “TRANSHUMANCE TRACKING TOOL (TTT) CHAD – DÉPARTEMENT OF GRANDE SIDO.” 2019, [Online]. Available: https://reliefweb.int/sites/reliefweb.int/files/resources/TTT_%20Dashboard%202_Moyen-Chari_EN.pdf.

[34] S. S. Shaida *et al.*, “Diversity and phylogenetic relationships of Glossina populations in Nigeria and the Cameroonian border region,” *BMC Microbiology*, vol. 18, no. S1, Nov. 2018, doi: 10.1186/s12866-018-1293-6.

[35] M. Desquesnes, G. McLaughlin, A. Zoungrana, and A. M. R. DaÂvila, “Detection and identification of Trypanosoma of African livestock through a single PCR based on internal transcribed spacer 1 of rDNA,” *International Journal for Parasitology*, p. 5, 2001.

[36] M. Kearse *et al.*, “Geneious Basic: An integrated and extendable desktop software platform for the organization and analysis of sequence data,” *Bioinformatics*, vol. 28, no. 12, pp. 1647–1649, Jun. 2012, doi: 10.1093/bioinformatics/bts199.

[37] S. Kumar, G. Stecher, M. Li, C. Knyaz, and K. Tamura, “MEGA X: Molecular Evolutionary Genetics Analysis across Computing Platforms,” *Molecular Biology and Evolution*, vol. 35, no. 6, pp. 1547–1549, Jun. 2018, doi: 10.1093/molbev/msy096.

[38] K. Tamura, M. Nei, and S. Kumar, “Prospects for inferring very large phylogenies by using the neighbor-joining method,” *Proceedings of the National Academy of Sciences*, vol. 101, no. 30, pp. 11030–11035, Jul. 2004, doi: 10.1073/pnas.0404206101.

[39] A. Paguem *et al.*, “Widespread co-endemicity of Trypanosoma species infecting cattle in the Sudano-Sahelian and Guinea Savannah zones of Cameroon,” *BMC Veterinary Research*, vol. 15, no. 1, Dec. 2019, doi: 10.1186/s12917-019-2111-6.

[40] S. J. Torr, T. N. C. Mangwiro, and D. R. Hall, “The effects of host physiology on the attraction of tsetse (Diptera: Glossinidae) and *Stomoxys* (Diptera: Muscidae) to cattle,” *Bulletin of Entomological Research*, vol. 96, no. 1, pp. 71–84, Feb. 2006, doi: 10.1079/BER2005404.

[41] C. E. Kirkpatrick, C. A. Terway-Thompson, and M. R. Iyengar, “Biochemical characterization of some raptor trypanosomes. II. Enzyme studies, with a description of *Trypanosoma bennetti* n. sp.,” *Can. J. Zool.*, vol. 64, no. 1, pp. 195–203, Jan. 1986, doi: 10.1139/z86-031.

[42] H. A. Garcia *et al.*, “Multilocus phylogeographical analysis of Trypanosoma (Megatrypanum) genotypes from sympatric cattle and water buffalo populations supports evolutionary host constraint and close phylogenetic relationships with genotypes found in other ruminants,” *International Journal for Parasitology*, vol. 41, no. 13–14, pp. 1385–1396, Nov. 2011, doi: 10.1016/j.ijpara.2011.09.001.

[43] P. Mallaye, L. Kohagne Tongué, N. Ndeledje, F. J. Louis, and H. Mahamat Hassane, “Transmission concomitante de trypanosomose humaine et animale : le foyer de Mandoul au Tchad,” *Revue d’élevage et de médecine vétérinaire des pays tropicaux*, vol. 67, no. 1, p. 5, Jul. 2014, doi: 10.19182/remvt.10154.

[44] J. R. Franco, S. Bernard, and L. Flévaud, “Olaf Valverde, Charles Wamboga, Pierre Marie Douzima, Josenando Théophile, Richard Laku & Victor Kande,” p. 28, 2016.

[45] J. Vourchakbé, Z. A. A. Tiofack, T. S. Kante, M. Mpoame, and G. Simo, “Molecular identification of *Trypanosoma brucei gambiense* in naturally infected pigs, dogs and small ruminants confirms domestic animals as potential reservoirs for sleeping sickness in Chad,” *Parasite*, vol. 27, p. 63, 2020, doi: 10.1051/parasite/2020061.

[46] L. Vanhamme *et al.*, “Apolipoprotein L-I is the trypanosome lytic factor of human serum,” vol. 422, p. 5, 2003.
[47] M. R. Rifkin, “Identification of the trypanocidal factor in normal human serum: high density lipoprotein.,” *Proceedings of the National Academy of Sciences*, vol. 75, no. 7, pp. 3450–3454, Jul. 1978, doi: 10.1073/pnas.75.7.3450.
[48] S. Alsford, R. B. Currier, J. A. Guerra-Assunção, T. G. Clark, and D. Horn, “Cathepsin-L Can Resist Lysis by Human Serum in Trypanosoma brucei brucei,” *PLoS Pathogens*, vol. 10, no. 5, p. e1004130, May 2014, doi: 10.1371/journal.ppat.1004130.
[49] F. Hawking, “The resistance of Trypanosoma congolense, T. vivax and T. evansi to human plasma,” *Transactions of the Royal Society of Tropical Medicine and Hygiene*, vol. 72, no. 4, pp. 405–407, Jan. 1978, doi: 10.1016/0035-9203(78)90136-0.
[50] D. Muhanguzi, W. O. Okello, J. D. Kabasa, C. Waiswa, S. C. Welburn, and A. P. M. Shaw, “Cost analysis of options for management of African Animal Trypanosomiasis using interventions targeted at cattle in Tororo District; south-eastern Uganda,” *Parasites & Vectors*, vol. 8, no. 1, Dec. 2015, doi: 10.1186/s13071-015-0998-8.
[51] E. R. Adams *et al.*, “New *Trypanosoma (Duttonella) vivax* genotypes from tsetse flies in East Africa,” *Parasitology*, vol. 137, no. 4, pp. 641–650, Apr. 2010, doi: 10.1017/S0031182009991508.
[52] E. R. Adams, P. B. Hamilton, and W. C. Gibson, “African trypanosomes: celebrating diversity,” *Trends in Parasitology*, vol. 26, no. 7, pp. 324–328, Jul. 2010, doi: 10.1016/j.pt.2010.03.003.
[53] A. L. A. R. Osório, C. R. Madruga, M. Desquesnes, C. O. Soares, L. R. R. Ribeiro, and S. C. G. da Costa, “Trypanosoma (Duttonella) vivax: its biology, epidemiology, pathogenesis, and introduction in the New World - a review,” *Memórias do Instituto Oswaldo Cruz*, vol. 103, no. 1, pp. 1–13, Feb. 2008, doi: 10.1590/S0074-02762008000100001.
[54] A. Cox *et al.*, “A PCR based assay for detection and differentiation of African trypanosome species in blood,” *Experimental Parasitology*, vol. 111, no. 1, pp. 24–29, Sep. 2005, doi: 10.1016/j.exppara.2005.03.014.
[55] A. Villa *et al.*, “Presence of *Trypanosoma theileri* in Spanish Cattle,” *Annals of the New York Academy of Sciences*, vol. 1149, no. 1, pp. 352–354, Dec. 2008, doi: 10.1196/annals.1428.016.
[56] A. Lanevschi-Pietersma, O. Ogunremi, and A. Desrochers, “Parasitemia in a neonatal bison calf,” *Veterinary Clinical Pathology*, vol. 33, no. 3, pp. 173–176, Sep. 2004, doi: 10.1111/j.1939-165X.2004.tb00370.x.
[57] B. Amato *et al.*, “A case of bovine trypanosomiasis caused by Trypanosoma theileri in Sicily, Italy,” *Parasitology Research*, vol. 118, no. 9, pp. 2723–2727, Sep. 2019, doi: 10.1007/s00436-019-06390-y.
[58] C. A. Hoare, “Studies on Trypanosoma grayi,” *Transactions of the Royal Society of Tropical Medicine and Hygiene*, vol. 23, no. 1, pp. 39–56, Jun. 1929, doi: 10.1016/S0035-9203(29)90831-2.
[59] “Utz et al. - 2006 - Trypanosoma congolense Procyclins Unmasking Crypt.pdf.” .
[60] N. A. Dyer, C. Rose, N. O. Ejeh, and A. Acosta-Serrano, “Flying tryps: survival and maturation of trypanosomes in tsetse flies,” *Trends in Parasitology*, vol. 29, no. 4, pp. 188–196, Apr. 2013, doi: 10.1016/j.pt.2013.02.003.
[61] G. Simo *et al.*, “Identification of different trypanosome species in the mid-guts of tsetse flies of the Malanga (Kimpese) sleeping sickness focus of the Democratic Republic of Congo,” *Parasites & Vectors*, vol. 5, no. 1, Dec. 2012, doi: 10.1186/1756-3305-5-201.
[62] R. N. M. Sehgal, H. I. Jones, and T. B. Smith, “Host specificity and incidence of Trypanosoma in some African rainforest birds: a molecular approach,” *Molecular Ecology*, vol. 10, no. 9, pp. 2319–2327, Sep. 2001, doi: 10.1046/j.1365-294X.2001.01339.x.
[63] L. Zídková, I. Cepicka, J. Szabová, and M. Svobodová, “Biodiversity of avian trypanosomes,” *Infection, Genetics and Evolution*, vol. 12, no. 1, pp. 102–112, Jan. 2012, doi: 10.1016/j.meegid.2011.10.022.
[64] J. B. Rayaisse *et al.*, “Towards an Optimal Design of Target for Tsetse Control: Comparisons of Novel Targets for the Control of Palpalis Group Tsetse in West Africa,” *PLoS Neglected Tropical Diseases*, vol. 5, no. 9, p. e1332, Sep. 2011, doi: 10.1371/journal.pntd.0001332.
[65] I. Tirados *et al.*, “Tsetse Control and Gambian Sleeping Sickness; Implications for Control Strategy,” *PLOS Neglected Tropical Diseases*, vol. 9, no. 8, p. e0003822, Aug. 2015, doi: 10.1371/journal.pntd.0003822.
[66] P. O. Odeniran, E. T. Macleod, I. O. Ademola, and S. C. Welburn, “Molecular identification of bovine trypanosomes in relation to cattle sources in southwest Nigeria,” *Parasitology International*, vol. 68, no. 1, pp. 1–8, Feb. 2019, doi: 10.1016/j.parint.2018.09.005.
[67] FAO, “Trypanotolerant livestock in the context of trypanosomiasis intervention strategies.” http://www.fao.org/3/y5832e/y5832e05.htm (accessed Sep. 07, 2020).

 Click here to access/download;Figure;Fig 1.tif

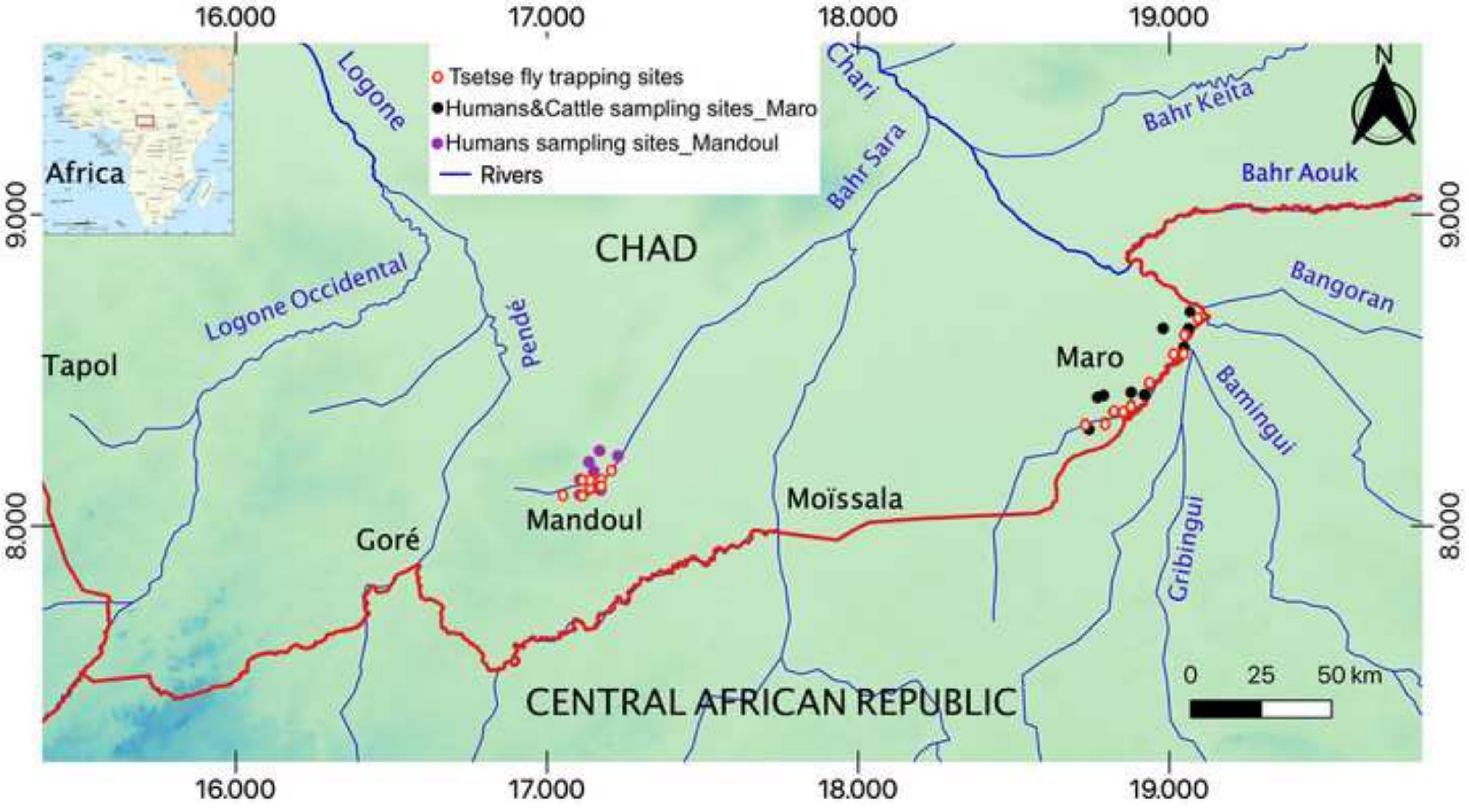

 Click here to access/download;Figure;Fig 2.tif

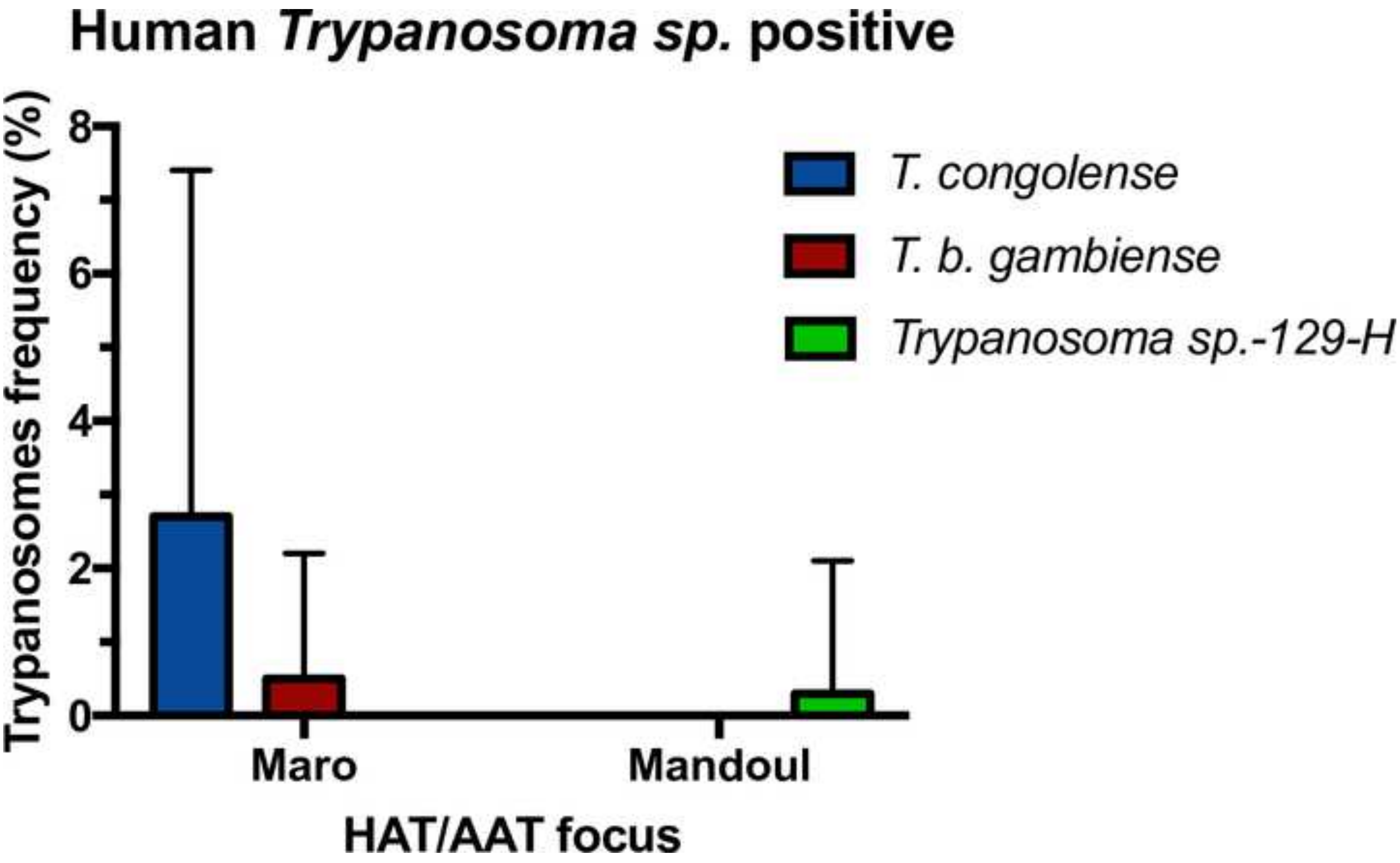

 Click here to access/download;Figure;Fig 3_.tif

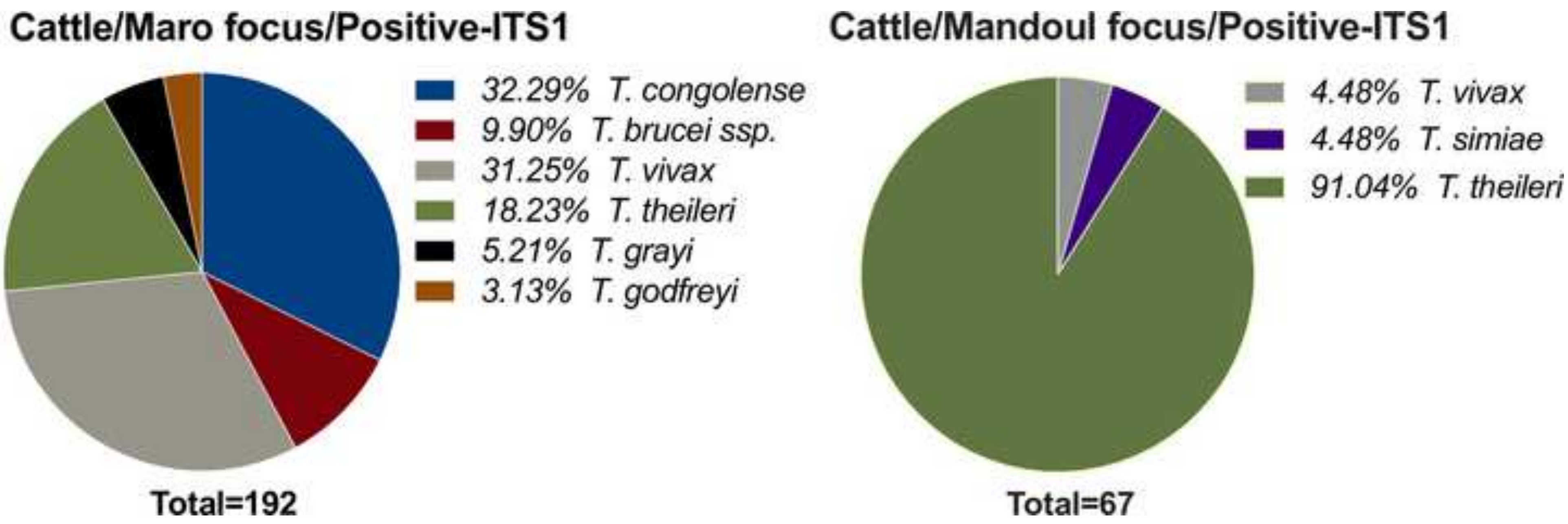

Figure

Click here to access/download;Figure;Fig 4_.tif

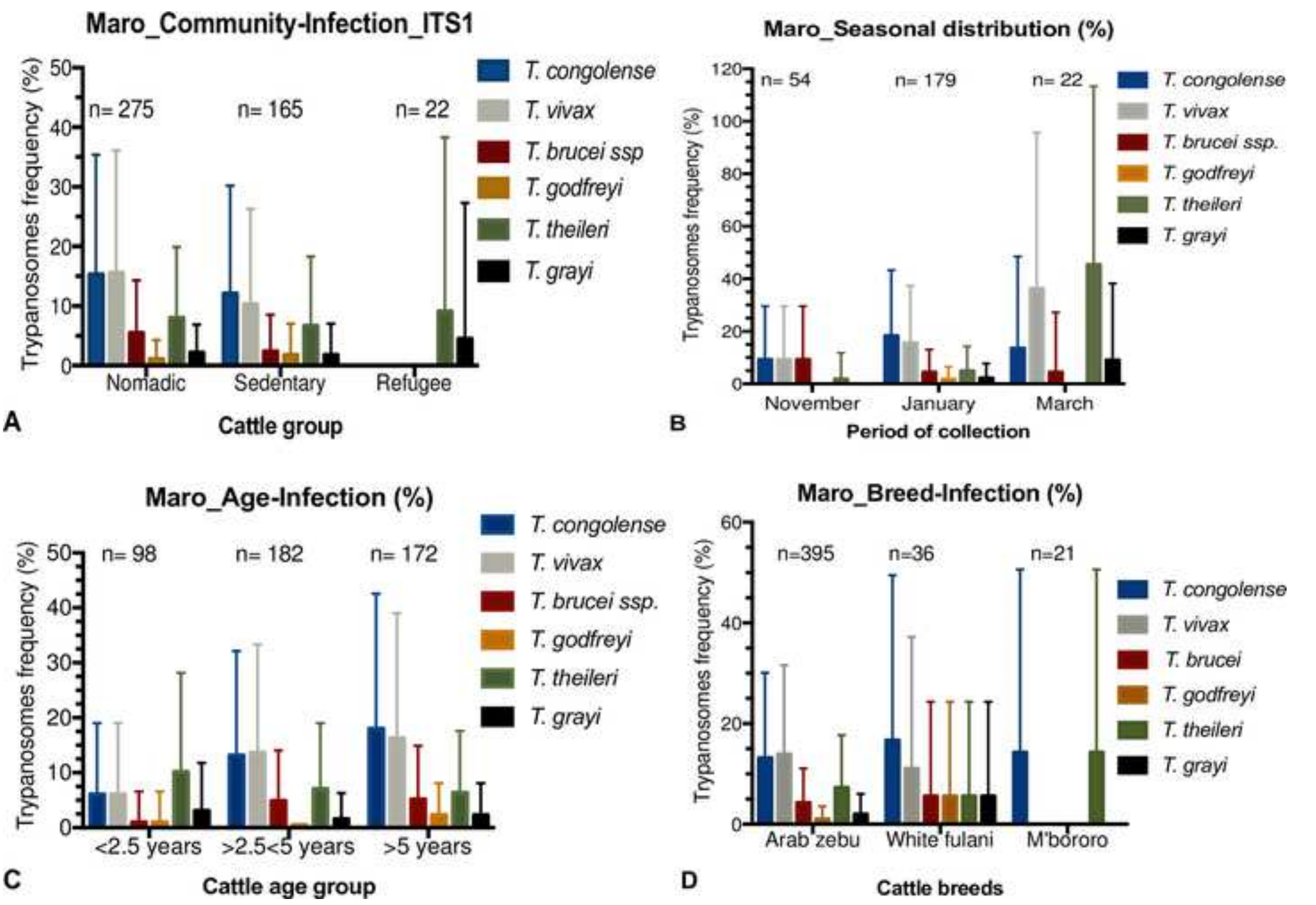

Figure

Click here to access/download;Figure;Fig 5 .tif

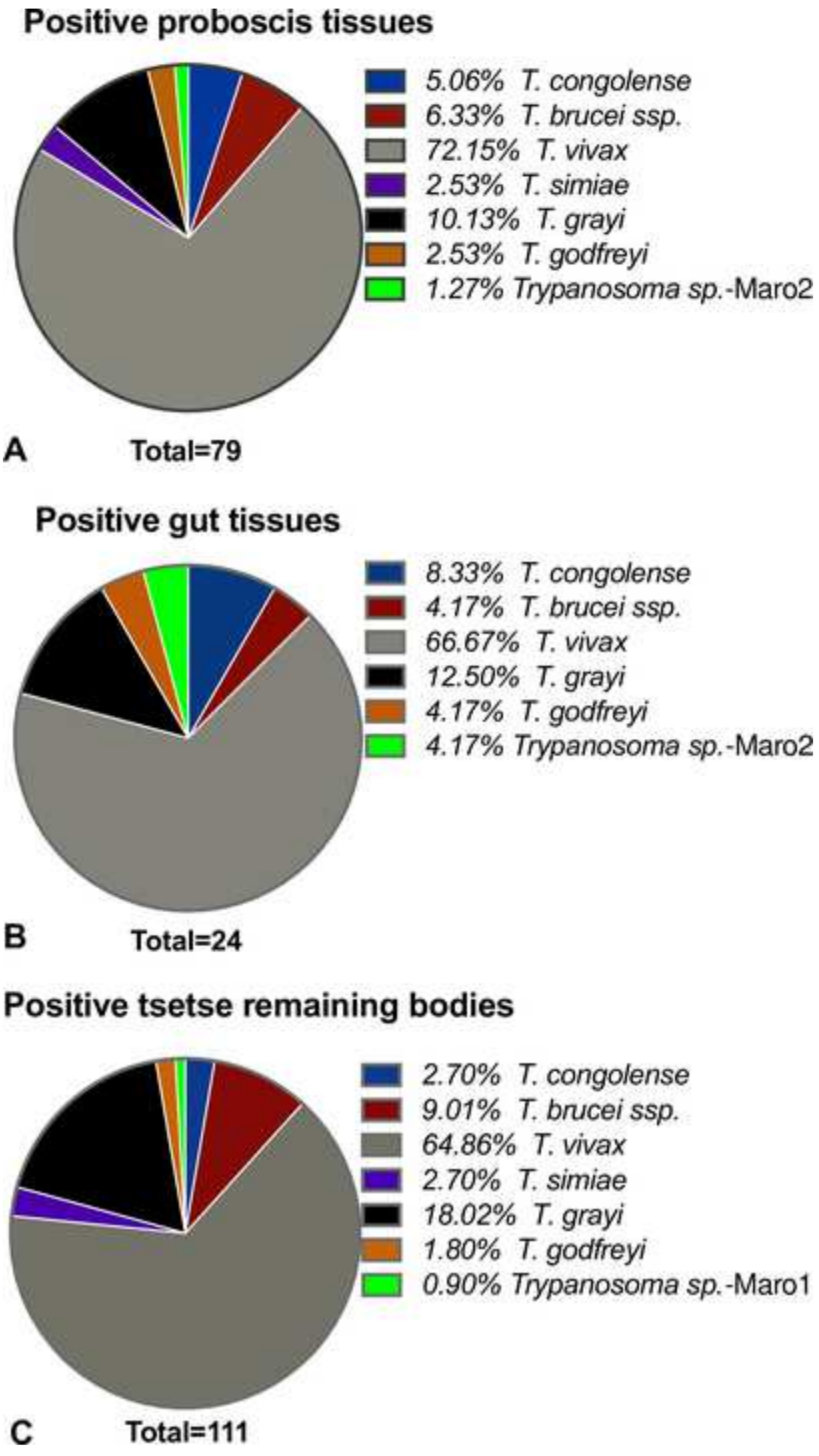

Figure

Click here to access/download;Figure;Fig 6.tif

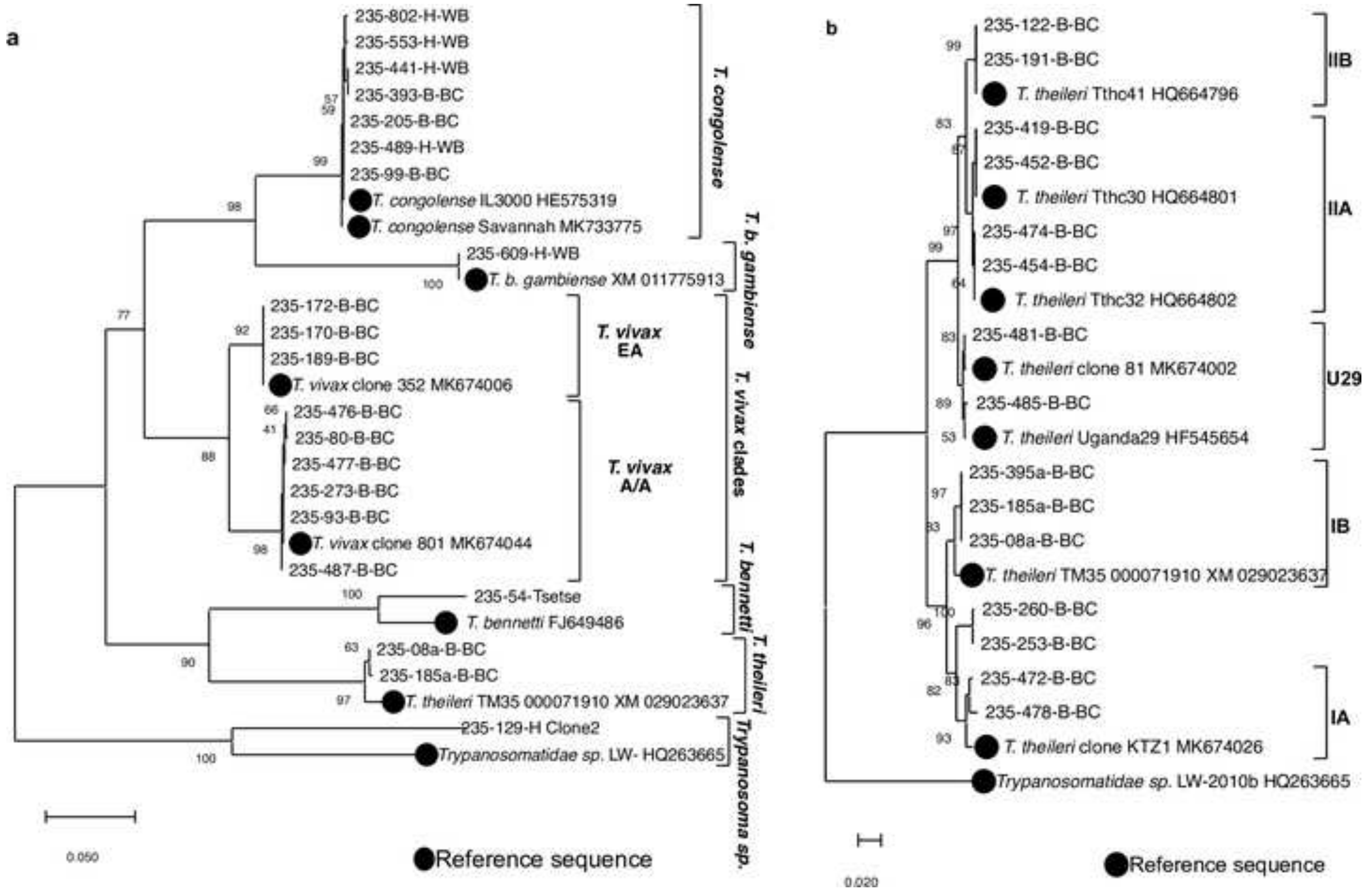

Click here to access/download
Supporting Information
S1 Text_Revised.pdf

Click here to access/download
Supporting Information
S2 Text_Revised.pdf

Click here to access/download
Supporting Information
S3 Text_Revised.pdf

Click here to access/download
Supporting Information
S1 Fig.pdf

Click here to access/download
Supporting Information
S2 Fig.pdf

Click here to access/download

Supporting Information

S1 Table.pdf

Click here to access/download
Supporting Information
S2 Table.pdf

Click here to access/download
Supporting Information
S3 Table.pdf

Click here to access/download
Supporting Information
S4 Table.pdf

Click here to access/download
Supporting Information
S5 Table.pdf

Click here to access/download
Supporting Information
S6 Table.pdf

Click here to access/download
Supporting Information
RawData_Human_PCR.xlsx

Click here to access/download
Supporting Information
Raw data_Cattle.xlsx

Click here to access/download
Supporting Information
RawData_Tsetse.xlsx

Responses to reviewers' comments on "Unexpected Trypanosoma species in humans, tsetse, and cattle, identified in Southern Chad: Mandoul and Maro sleeping sickness foci" (manuscript ID PNTD-D-20-01868)

The manuscript title is changed to: "Diversity of trypanosomes in humans and cattle in the HAT foci Mandoul and Maro, Southern Chad - A matter of concern for zoonotic potential?"

Please find below the detailed answers to the comments and questions of the reviewers. This also includes the 5 particular notes highlighted by the editor, and in the last pages, the answers to "reviewComments" received as a separate file.

First of all, we thank all the reviewers for their positive remarks, their agreement on the importance of the study, and their detailed review of the manuscript, including suggestions, comments, and questions.

Methods

Reviewer #1:

- A clear set of aims that link to each section of the Methods is not provided

We included more information concerning the study aims that link to the methods of this study. This study was undertaken to investigate the circulating trypanosomes, including the occurrence of potentially zoonotic species in humans and livestock, and in their biological tsetse vector in two active HAT foci, Mandoul and Maro. See Introduction from line 123 to line 133.

The detailed protocols in supporting material S1, S2 and S3 Texts are now shifted in shorter forms in the revised manuscript (Method sections from line 134).

- There is no clear study design or description of statistical analysis used to inform the results.

We provided additional information for the study design. See line 156 - 162 (for the humans) and line 173 - 178 (for cattle). More details are provided in S2 Text from line 5 to line 18, and line 34 to 37.

We want to thank the reviewer for the remark to include a description of statistical analysis used to inform the results. The statistical analysis applied is provided in line 257 to 267.

- No power analysis provided

Specification of the power analysis is included in the manuscript as recommended by the reviewer. See line 163 to 171 for the humans and line 178 to line182 for the cattle. We based on this power calculation, with an accepted margin of error of 5% and 95% confidence

interval, to target the sample size per HAT focus (Mandoul and Maro) and per host (human and cattle). Unfortunately, in Mandoul only 78 cattle have been collected, by far less than the expected sample size due to an anthrax outbreak during a scheduled survey, as stated in the result part (line 315-316).

Reviewer #2:

- The methods need to include details on the sample frame for both human and animal, random sample method is stated but lacks details on either the number of samples that the survey set out to collect (cattle or human) or clear details on how the individuals were included for the sample at each field site.

We thank the reviewer for these suggestions. We included the information on the method of a random selection of households/herds for participation. See line 156 to 162 (for the humans) and line 173 to 178 (for cattle). More details are provided in see S2 Text from line 5 to line 18, and line 34 to 37.

The power calculation for the necessary sample size for the human survey is described from line 163 to 171, and for the cattle survey is from line 178 to line182. We faced an issue during the survey to collect cattle samples in Mandoul, because of an anthrax outbreak (stated in line 315 - 316). Therefore, we couldn't reach the target sample size, as only a few samples (78) have been collected. However, the sample size was reached in Maro for both humans and cattle.

- In lines 432-435 of the discussion states that the owners presenting cattle for sampling [which is not random sampling. Or are you suggesting that herders could have separated healthy animals from the herd to only take unwell individuals to the sample site?

We thank the reviewer for the valid discussion point. Deviations of random sampling for cattle are now stated in the method (line 175 to 178) and then discussed in line 564 to 567. We further want to highlight that there are two aspects along that line for the animal selections. First, we aimed for a random study where we were able to select the herdsmen by following this strategy. However, when selecting the cattle, especially in the nomadic settlements, the selection has been influenced by the shepherds that select the animal based on their own interest (taking perhaps more sick than healthy animals). For the sedentary group, shepherds did not have many animals (4 heads approximately). Therefore, a selected herdsman will provide all its animals unless some are absent during the survey. As such, the random selection of the animals was biased. Nevertheless, the statistical analysis applied in the study was to compare variables in this cohort without making an extrapolation for the entire area.

- The statistical evaluation that is referred to in the results section has not been identified.

A description of statistical analysis applied to inform the results is provided in line 257 to 267.

- Results section refers to a questionnaire (line 284), which is absent from the methods.

The description of the questionnaire is now shifted from the S2 Text to the main manuscript (line 183 to 187). Responses to the questions are provided in excel file "Raw data_Cattle".

- Cattle age forms part of the data analysis and discussion, how was the age of each animal established?

We thank the reviewer for the question. As stated in S2 Text (line 44), the age was given by the herdsmen and to some extent confirmed by the veterinarian present during the fieldwork. They are grouped in three categories young (<2.5 years), mature (between 2.5 and 5 years), and elders (above 5 years). Thus, the relative age given by the herdsmen will be within the age set subsequently.

Reviewer #3:

- Sample size and selection
  I tend to think that the methodology applied was appropriate for the study, although probably the objectives of the study should be stated more clearly. Particularly, I appreciate the sequencing, missing in other similar articles. On the other hand, sample sizes seem a bit low. Although clusters of villages, semi-nomadic, nomadic and military camps are mentioned, I'm not clear how participants (households and cattle) were selected/recruited: was there any randomisation that may allow to assume that the samples were representative? How comparable are the samples and the populations? Was there any sensitization campaign?

We thank the reviewer for the comments and questions.

- Sample size; selection; How comparable are the samples and the populations?

We based on the sample size proportion calculated using the open-source Epidemiologic statistics for public health software Version 3.01, knowing the human and cattle populations in these areas, as stated in the Manuscript (Line 163-171 for the humans and line 178 - 182 for the cattle). Sample sizes per focus (Mandoul and Maro) and per host (human and cattle) were obtained using this software with an accepted margin of error of 5% and 95% confidence interval. However, in Mandoul, the number of collected cattle samples was by far less than the expected sample size due to an anthrax outbreak, as stated in the result section (line 315-316).

We provided information on human and cattle selection. Line 156 - 162 (for the humans) and line 173 - 178 (for cattle). More details are provided in S2 Text (line 5 - 18, and line 34 – 37). For human surveys, households were drawn by chance. However, members of each selected household were automatically included, unless for individuals who did not consent to participate or children under 5 years old. The villages were selected randomly at each site, except the military camp that was at the request of its inhabitants.

For the cattle, the herdsmen (representing their herd) were selected randomly. However, the cattle were chosen partly by the herdsmen themselves for the nomadic settlements (precision added, line 176). For the sedentary group, breeders did not have many animals (approximately 4 heads or so). Therefore, a selected breeder will provide all its animals unless some of them were absent during the survey.

The comparability of the sample sizes and populations is crucial to our study. The human survey reached the required sample sizes (line 271) as calculated in advance (line 170), as well as the cattle in the Maro focus (Line 314 as calculated in line 181). However, for cattle from the Mandoul focus, the size is small (Line 314 as calculated in line 181).

- I tend to think that the methodology applied was appropriate for the study, although probably the objectives of the study should be stated more clearly.

We agree with the reviewer, and we have stated the objectives more concisely in the Introduction (line 123 to line 133).

- Was there any sensitization campaign?

Yes, the sensitizations have been done prior to the surveys where the team visited the locations and made contact with the local communities. In these visits, the objectives of the study were explained. This was added in S2 Text Line 7. Furthermore, we repeated the meetings during the recruitment day, a day prior to the blood collections.

- Ethical considerations
  How were cases managed (i.e. human or cattle +ve samples? For example, were they and PNLTHA notified?

This work was supported in the beginning by both PNLTHA and IRED, as mentioned in the Acknowledgments section. The personnel of these institutions are well known in these foci and are working in close contact with the human and animal health services, as well as the local communities. Therefore, suspected human cases were automatically taken care ofby the local health services which follow the protocol of the diagnosis and treatment depending on the phases of the disease if the cases are confirmed.

- Were +ve cattle treated, not able to recover…

For the sick cattle, especially those suspected for AAT, a veterinarian who was supporting the team and the local veterinarian technician manage to treat the animals. Also, this depended on the protocol of treatment depending on the health status and symptoms of the animal, recommended by this animal health personnel.

All these parallel supports were discussed with the authorities in charge of Human and animal health sector, including PNLTHA, the Regional delegations as well as the local Human and animal health services, and the administrative authorities.

- Study areas:
  It would be relevant to describe the main differences between the Mandoul and Maro foci/habitats. For example:
  - Mandoul: Tsetse are restricted to the swaps formed in the southern limit of the Mandoul river. The tsetse distribution is limited in the South by the springs. As the river flows north, the swamp deteriorates into a marsh habitat, unsuitable for tsetse. Therefore, the population is isolated. Vector control operations with annual deployment of Tiny Targets started in 2014.
  - Maro: In the southmost sections of Chari/Sido rivers, where rivers mark the border with CAR. Tsetse habitat configured by is the thin riverine vegetation along the banks

of the rivers. Vector control operations started in the Chadian part in 2018, with annual deployments of Tiny Targets. No similar operations have been implemented across the border.

We thank the reviewer for the suggestion to include this important information. It is included (Line 114 -117 and line 142 - 152) and more details are in S1 Text.

- Trypanosomes in tsetse:
  Any reason not to analyse salivary glands for mature infections of *T. brucei*?

It was one of our interests. Unfortunately, most of the tsetse flies trapped were found dead due to the high temperature and the low relative humidity during the surveys in these regions (Line 464). Therefore, we faced a technical issue for dissecting the salivary glands and screen for mature infection of *T. brucei*.

Results

Reviewer #1:

- Due to the absence of a discrete set of aims and the absence of details of study design (e.g. justification for selection of villages/ individuals), power analysis, or statistical analysis the results do not match the analysis plan. There are no estimates of uncertainty provided for trypanosome prevalence.

Overall, the study aimed to look for the circulating trypanosomes in the three host from the two HAT foci and their genetic diversity. Since the selection of cattle especially was biased, a stating prevalence cannot be adapted for the study to extrapolate for that of the whole population. However, it might be possible for the human section. Therefore, the term “prevalence” was not used but instead “frequency” from our point of view is suitable. Estimates of uncertainty (using the Clopper-Pearson binomial test with the lower and upper limits of the 95% confidence interval) was now added to all frequencies of trypanosomes. Power and statistical analysis are now included.

Reviewer #2:

- Due to the two study areas being detailed I suggest the author presents each set of findings first as overall findings (2 areas combined) then Maro and Mandoul individually. Thereby standardising the order. To highlight the issue for a reader unfamiliar with the study; between line 174 and 177 it was not until I had already been confused by the reported percentages 0.5 and 2.7 (which I thought should have been 0.28 and 1.53) that it becomes clear the results are only Maro and not the whole study.

We want to thank the reviewer for the suggestion. It is more clarified now (e.g., line 283).

- Attention needs paying to typographical errors, to give some examples: Line 258 'at the tree time points' and line 341 where 'of' is missing after 13%.

Thank you to remained us for typographical errors. We corrected several spelling errors starting from the abstract through the conclusion.

- Further, throughout the results section the decimal places used in numbers is

inconsistent.

The suggestion is taken into account. We thank the reviewer for this precision.

- There are sections in the results that belong in the discussion. examples; line 262-263 and 276-278.

We moved them.

- Figure 1, the colour used for tsetse trap location is very hard to identify against the green base map, also the text in the image seems to be quite blurred.

We thank the reviewer for this remark. We improved all figures.

- Table 1 should be in the results section and referred to in the discussion.

We moved it and added a paragraph for its description (Line 510 to 523).

Reviewer #3: Human cases

- Two human cases of *T. b. gambiense* in Maro seems to suggest a relatively high prevalence. Do the authors consider that this may be a good representation of the prevalence in the foci? Or, may this relate to the low sample size and/or bias in the selection of participants? Or, 'oversensitivity' of the technique?

The households were randomly selected, and the sample size sufficient to represent the population in the areas. We used the term frequency for the whole study just for the consistency of the manuscript, since the cattle selection was biased. However, we could extrapolate that the *Trypanosoma* prevalence in the entire population of Maro is high. This could also be influenced by the techniques used since nested PCR can amplify traces of DNA (down to 10 pg).

- I assume the main point of this finding is proving the presence of *T. b. gambiense*?

While *T.b.gambiense* is the main health concern, we also want to raise awareness of the potentially zoonotic species.

- Do the forms include a question about previous travels?

We thank the reviewer for the interesting discussion point. Yes, and also other information such as previous HAT infection and current health status or age and gender (see comment in the discussion part line 590 and line 595).

- Tsetse:
  Species not mentioned.

We agree that this might be useful information, but as tsetse were not the focus of the manuscript, we found this too much to be described. Nevertheless, it is now incorporated (Line 202 - 203 in the method section and Line 462 - 463 in the result part).

- Figures
  Figures are small and low definition. They are difficult to read.

They are now adjusted for better visibility.

Conclusion

Reviewer #1:

We want to inform you that the discussion section is revised as it was recommended, and major modifications have been incorporated.

- It is not made clear why this study helps to better understand the situation in Chad. Without detailed methods and robust comparison between the two foci in the results, no conclusions can be drawn with respect to the differences between Mandoul and Maro.

We want to investigate the diversity of trypanosomes and see if Maro have the same pattern of the species diversity since the tsetse control has not been primed at the time of the survey like it was for Mandoul focus. In conclusion, as the aim is stated and methods described, the results showed a difference between the two areas in terms of species diversity and also in terms of tsetse fly presence. This could be due to the vector control initiated in the Mandoul which decreases the tsetse population (as only a single fly was caught see line 518) and thus the pathogenic trypanosomes (line 522, Table). It could also be due to the geographic location of the Maro bordering the Central African country, involving transhumance activities.

Reviewer #2:

We want to inform you that the discussion section is revised as it was recommended, and major modifications have been incorporated.

- line 679-680: Tiny targets are only used as tsetse control not elimination, also note tinny is a spelling error.

Thank you for pointing this out, it is corrected.

- Line 684-686: Maro is now receiving parasitological evaluation and regular control of tsetse (as you state in line 638).

The following note was deleted as the discussion is recast. Nevertheless, the message appears in line 717.

- The close of the conclusion is stretching. The assertion that to achieve the HAT elimination goal atypical trypanosomes need considering is at odds with the earlier statement in lines 399 - 403 where you highlight that the occurrence of non-HAT species of trypanosome detected in a human is likely the detection of an unviable infection.

The conclusion is now recast, see Conclusion line 767. The discussion of atypical HAT versus detection artefacts is crucial. Of course, we do not know if the trypanosomes (including atypical species) observed in humans using sensitive molecular techniques are transient or established infections. But the presence of much diversity in human blood samples is worrying, especially in the case of the individual from the Mandoul focus with an unknown *Trypanosoma sp*. However, since this man previously had a HAT-gambiense infection and

was cured of it but has still sequels of the disease, and showed evidence of an unknown trypanosome, this situation needs to be closely monitored. It must not be neglected, as it might be an opportunistic infection. Note: we asked those in charge of supervising the HAT-disease especially the personnel of Bodo's hospital (local health service personnel who are working in close contact with the National Program, PNLTHA), they confirmed that he is HAT-negative for all the last screening campaigns.

Reviewer #3:

We want to inform you that the discussion section is revised as it was recommended, and major modification have been incorporated.

- 385-388 "To evaluate risk assessment of HAT, regular screening campaigns of humans for *T. brucei gambiense* using microscopy, Loop-mediated isothermal amplification (LAMP) and RDT-kit have been undertaken by the Ministry of public health and its partners within the historical HAT-foci in Southern Chad". At the moment, case definition in Chad for HAT is based on CATTs test at certain titer dilution (I think 1/16). Other direct (e.g. LAMB, mAECT) or indirect (e.g. trypanolisis) tests are not as common as in other countries.

The description is modified see 583 to 587. We thank the reviewer for the suggestion.

- 397-399 "It is the first study conducted in two Chadian HAT foci using a molecular identification tools, i.e. ITS1 amplification supported by gGAPDH analyses and sequencing to screen at the same time the tsetse fly vector, as well as human and cattle as definitive mammalian hosts". I know there are differences, but please see https://www.parasite-journal.org/articles/parasite/full_html/2020/01/parasite200101/parasite200101.html

Thank you for bringing the study to our attention which we included in the discussion section (line 589). There are still differences in the approach of the two studies as well as the target hosts. Nevertheless, it gave some other values concerning the presence of *T. b. gambiense* in the foci.

- "Diversity and distribution of trypanosomes in the area":
  431-432 "The evidence of high trypanosome frequency in cattle (see S5 Table for details) vigorously supports our observation of high frequency in tsetse flies" In my view, the data shared by the authors show a clear distinction between the findings between Mandoul and Maro. Thus:
  Mandoul:
  - Hard to find flies (because probably there are not many)
  - T. theileri is predominant in cattle (transmitted mostly by tabanids), with some T. vivax (which can be transmitted by other biting insects)
  - Neither T. congoloense nor T. brucei found (transmitted by tsetse)
  - Do the above imply that the incidence of tsetse-transmitted tryps might be approaching zero?

From this investigation, we can assume that in the Mandoul area, Tiny Target's implementation reduced the tsetse population and thus trypanosomes which this vector can transmit. This has also been stated in an already published paper (Line 146 and 714, Mahamat et al., 2017). Possibly tsetse-transmitting tryps might be approaching zero. However, more data and coverage from other sites in the focus than those visited (by including tsetse, humans, and reservoirs) could give more light to this assumption.

- Maro:
  - Tsetse were collected in relatively good numbers
  - T. congolense predominant
  - More variability in Tryps spp
  - Do the above suggest that in Maro tsetse are vectoring human and cattle Tryps?

We draw the conclusion that yes indeed this is suggested, see Discussion line 690, 717 and 724.

- Could the vectors explain the differences in the seasonality for each Tryp spp?

Not for all trypanosome species, but certainly for *T. congolense* and *T. brucei* as it is already known that in the dry season when the tsetse populations are reduced, their incidence in the herds is also reduced. Nevertheless, the frequency of *T. vivax* could be high in the dry season as other biting insects could also transmit it (Line 750 - 756). Therefore, this could also be the case for the other trypanosome species that other vectors can transmit.

- The authors mention in the discussion that cattle participating in the study might have been biased, as perhaps owners presented sick animals hopping for a treatment. This might explain a relatively high infection rate in cattle. Was there any bias in the selection of human participants? (see above)

See line 156 to 162 (for more details see S2 Text line 14-18: "In order to proceed with the selection, households were numbered and a total of 6 to 16 households (see the paragraph below) in a selected village were drawn for participation. A chosen household included all its members automatically".

The number of the households per village depends on the number of members of selected households. *i.e.* if one household has for instance 16 members, the chance to include many following households in the same village is reduced, since we targeted to include maximum of 70 participants per village.

- 440-442 "The relevant result emerging from the data is the high frequency (Table 1) and diversity (see S5 and S7 Tables for details) of trypanosomes in cattle in both foci". As mentioned above, I don't see it that way. I think the most relevant result is precisely the differences between both sites.

Thank you for dragging our attention on that aspect. For this reason and those mentioned above, we reconstructed the discussion, which now includes sections that follow the suggestions, *e.g.,* line 568 and 573.

- 442-444 "Another finding and not the least was that, in the Mandoul focus *T. theileri* was abundant, whereas only very few *T. vivax* and *T. simiae* were found and there was no evidence for *T. congolense*, neither in cattle nor in humans". This seems a repetition of the results without the discussion. How do the author explain this result? See above

This is taken into account in the new discussion, and the tendency of trypanosomes presence or absence in a focus is explained (line 722-736).

- "Diversity of known trypanosomes"
  446-450 "The *T. b. gambiense* DNA that was detected in one child and one old man in the Maro focus, confirmed the presence of the parasite in the area…". The authors state that the two cases proved the presence of *T. b. gambiense* in Maro. Instead of "Interestingly, none of the samples collected in Mandoul was *T. b. gambiense* positive, and this is in agreement with the reduction of its incidence reported by Mallaye" the author should be consistent with the way reporting for Maro, i.e. confirmed presence vs. non-confirmed presence. Is this "in agreement with the reduction in the incidence"? Probably yes, but this is not proof of it. With this small sample size, rather than using the data to infer an epidemiological situation, I think it would be more honest to use the results in terms of proven or non-proven presence of the parasite.

Thank you for the suggestion which leads us to modify the structure of the discussion (line 587 - 594).

- 456-459 "Interestingly, *T. vivax* clustered in two clades, with both present in samples collected from the Maro focus. One of these clades groups with the East African *T. vivax* (EA) with a strong homology and high similarity. This clade EA was described for the first time in Tanzania [49], then in Nigeria [28] and in Cameroon [31] in 2019, and now in Chad in this study". If the clade has been identified in Nigeria and Cameroon, why is it so surprising finding it in Chad?

In the literature, it was identified as the East African clade, and there is evidence of its presence in West and Central Africa reported recently. Therefore, we wanted to point out that this is a clade not restricted only to Eastern Africa, and its identification could be due to the sequencing data that are now more involved (Line 643).

- Any speculation about how the dispersion and diversity of *T. vivax* across Africa?

T. vivax is widely spread, including tsetse-free areas as well as many animal species such as camel, cattle, etc. are infected. The available sequences in the database and previous reports pointed out so far from our knowledge, the two clades (EA and AA) with a mention of large distribution of the A/A clade as mentioned in line 647. So, thorough sequencing of samples from different locations and origin in Africa might lead to identifying other clades (subspecies) as already reported in the literature (Adam et al., 2010).

- 476-477 "*T. theileri* was not observed in the tsetse samples which confirms its transmission independently from tsetse." The sentence if quite vague. We know that

T. theileri is mostly transmitted by tabanids. In my opinion, the main point to be made about *T. theileri* in Maro and Mandoul is that the predominant Tryps spp in Mandoul is not transmitted by tsetse (because tsetse are close to annihilation); on the other hand, Tryps spp in Maro are more diverse.

Thank you for suggesting this. It is taken into account (line 579).

- 487-489 "The identification of *T. godfreyi* and *T. simiae* in different tsetse fly tissues and cattle, common across the African continent [56], [57] suggests that the vector would have fed on wild animals such as warthogs". Why?? The population of domestic pigs are far larger than that of warthogs. Presumably, T. simiae are a problem for pigs.

This part was found less necessary and was deleted from the revised discussion.

- 513-514 "The more the animals get old, the more their susceptibility to pathogenic species is high, as age-related resistance to trypanosomes is recognised" As explained by Vale, Torr and others, the body mass of the host is directly correlated to the attraction of tsetse; thus, old (and heavy) cattle attract more tsetse than calves.

Thank you for the suggestion. See line 410.

- Relevance for the area(s)
  624-629: If by the "elimination campaign" the authors refer to the use of SIT, as far as I know, this has not started yet. No release of sterile males was done during the time reported in this manuscript. The elimination project (using SIT) was attached to an existing project aiming to eliminate HAT transmission (not necessarily tsetse). And yes, that is being done using Tiny Targets, although PATTEC has nothing to do with it.

This is essential information. The "elimination campaign" term is changed with tsetse control when it addresses the issue of tsetse.

- 638-639: There isn't any tsetse elimination campaign in Maro. There is a tsetse control operation, aiming (in addition to case detection and control) to eliminate the transmission of HAT. It started in 2018. Note than the river is the natural border between Chad and CAR (sometimes the river is just 5-10 m wide, some time a few hundreds) and all the efforts were done on the Chadian side, only.

The correction has been made concerning the tsetse control, e.g. line 717.

- Conclusions:
  677 It might be worth mentioning somewhere that the current WHO goal is the "elimination of HAT transmission" by 2030.

The correction has been made see, line 768-769.

- 679 As above, the aim of the Tiny Targets is not the elimination of tsetse. In Mandoul, and Mandoul only, there is a new project to implement SIT, but (if I'm not wrong) the release of sterile males has not started yet.

The correction has been made see above.

Editorial and Data Presentation Modifications?

Reviewer #1:

- (No Response)

Reviewer #2:

- Attention needs paying as references are out of order, eg: line 629-630 references Mahamat et al [23] but in the references on line 777 [23] is Targeting Tsetse on LSTM's website.

Thank you for the kind remark. We check all the references, including the stated ones. With the restructuration of the discussion, some of the references are deleted.

Reviewer #3:

- 68-69 "Despite the WHO goal to eliminate it by 2020, HAT is still a public health problem, because 70 million people in 36 sub-Saharan African countries are at risk of infection". Actually, the 2020 goals established by WHO were globally achieved by 2018; that was according to their own definition of "public health problem" (i.e., <1/10,000 cases, >90% of endemic foci; and <2,000 cases worldwide). The current goal is the elimination of transmission.

Description modified see line 73. Thank you

- 87-89 "The main insect vector in Africa are flies of the genus Glossina (Glossinidae: Diptera). However, the parasites can also be transmitted mechanically by other biting flies such as tabanids and Stomoxys". Although, this is true for some Trypanosoma spp (e.g. *evansi*, *theileri*, *vivax*…), I find the statement a bit too general.

We prefer to leave it like this from our point of view as it gave a global idea to the reader (line 92). However, we add a sentence concerning *T. theileri.*

Summary and General Comments

- Use this section to provide overall comments, discuss strengths/weaknesses of the study, novelty, significance, general execution and scholarship. You may also include additional comments for the author, including concerns about dual publication, research ethics, or publication ethics. If requesting major revision, please articulate the new experiments that are needed.

Reviewer #1:

- Please see attached document.

The comments into document are all taken in consideration in this letter (see in the last sections below).

Reviewer #2:

- This represents interesting and insightful work, which I want to see published. There are aspects of the paper that need refining which will greatly benefit the reader. Details of some methods that have been used are absent from the appropriate section. Results section will benefit from being revised.
  The assertion that atypical trypanosomes could threaten HAT elimination should be accompanied by a reminder of your sentiment in lines 399-403 thereby ensuring the reader takes away an unbiased conclusion.

We thank the reviewer for the positive remarks. The suggestions were considered.

- From my knowledge the fieldwork was undertaken for this project predates work on eradicating tsetse in Mandoul, there was ongoing tsetse control by the consortium of PNLTHA, FIND, IRD and LSTM as part of the drive against gHAT. As such reference to eradication in Mandoul is misleading and should be reworded as ongoing tsetse control. The article repeatedly refers to the FIND, IRD, LSTM operation as tsetse elimination, which is inaccurate, the project is objective to control tsetse to thereby reduce the risk of infection in gHAT foci and not to eliminate tsetse, this should be addressed throughout.

We thank the reviewer for remaining us the important terms. “tsetse elimination” term is replaced in the revised version by “tsetse control”.

- Abstract refers to trypanosomosis in humans, this should be trypanosomiasis for disease in humans.

Corrected was done, see Abstract line 19. Thank you

Reviewer #3:

- The authors present in the manuscript a comprehensive study of the two main HAT foci in Chad, including parasitology, epidemiology, entomology, population genetics… The amplitude of the study has advantages and disadvantages. On the one hand, it is very informative (the authors report the Tryps spp circulating in both foci), but on the other hand, it lacks an obvious ‘selling point’ that may appeal potential readers. After reading the manuscript with interest, in my opinion, the data contains some implications that are not properly highlighted and discussed, and other perhaps less important with too much detail. The weakest part is probably the discussion, when the authors have the chance to convince readers why their work is relevant. It is indeed useful to know the Tryps spp circulating in humans, cattle and tsetse vectors, but in practical terms, some spp are more important than others.
  For example, the authors dedicate extensive paragraphs to discuss things like ‘atypical HAT’, or Tryps spp than hardly cause any symptoms in livestock; spp of less medical or veterinary importance can be mentioned briefly, and expand on, for example, T. b. gambiense, T. b. brucei, T. congolense, T. vivax and perhaps, T. simiae. Conversely, what it is probably the most interesting points of the research are not discussed in the

length they deserved.
My first advise for the authors would be to recognise the weakest points in the data, and highlight the strongest ones. For example, the sample size seems low, and probably the recruitment of cattle and people were not randomised. That means, the %s reported may not represent the whole population (not valid for epidemiological extrapolations). That is not a big problem, as they can still report relative frequency of the different parasites (in flies, cattle and human), and for those less common, presence confirmed vs. presence non-confirmed.
As a reader, I would like to see the differences between both foci: what those differences are and why. To show this, the reader needs to understand from the beginning what makes Mandoul and Maro so different:
- As a tsetse habitat, Mandoul represents an ecological island: it is isolated, and the risk of reinvasion is very low.
- Before 2014, when the only approach to control HAT relied solely on case detection and treatment, Mandoul was by far the most active HAT focus in Chad (some numbers would be good). The addition of vector control reduced dramatically the number of cases to a handful.
- As the number of cases in Mandoul declined, PNLTHA increased the efforts to identify new cases in areas that up to that moment were considered of less importance (including Maro). I'm quite convinced that the increase in the number of cases in Maro was the result of the additional efforts (at least in part).
- The tsetse habitat in Maro, on the other hand, is open for reinvasions. The deployment of targets started in 2018 and probably did not have any impact in this study. In the long term and due to the differences, we don't expect to have the same results as in Maro.
With this background, the data suggest that the presence of Tryps in Mandoul can be explained without tsetse, but the opposite is true in Maro, whereas in Maro tsetse-transmitted Tryps were found in larger proportions, and the spp variability was much greater. The difficulties to collect tsetse in Mandoul seems to support this idea. Tsetse-transmited Tryps are of medical or veterinary importance (e.g. T. b. gambiense, T. congolense…), so that looks like a good thing. This could lead into a subsection in the discussion: practical implications; or what we need to do in Maro, so it can look more like Mandoul.
This contrasts with the recently published paper by Vourchakbe et al https://www.parasite-journal.org/articles/parasite/full_html/2020/01/parasite200101/parasite200101.html
Vourchakbe and colleagues reported a higher proportion of T. b. g. in Mandoul, compared to that of Maro (Tryps found in goats, sheep, dogs and pigs, and they did not sequence the amplicons).
Probably there isn't enough data to support big statements about potential trypanotolerant cattle breeds. We also need to consider other variables associated with breeds: for example, Arab zebu might look like trypanosensitive according to infection rates, but as the breed of nomadic heads, they are also exposed to different risks. Cattle are specially exposed when they cross rivers, and nomadic cattle does

that more often than stable cattle. Another factor to consider (commented briefly by the authors) is the potential correlation between seasonality of certain Tryps spp and their vectors (e.g. tsetse, horseflies…). And, talking about vectors, the authors should mention the name of the tsetse, at least once: Glossina fuscipes fuscipes.
If the authors agree with me, they should probably indicate more clearly in the introduction the objectives of the study (e.g. to compare presence/absence of different Tryps spp -- in cattle, people and flies -- in Mandoul and Maro, or something along those lines). Then, the methods will fit with the objectives easily.
In agreement with that, I would also recommend a change in the title. First, I'm not sure why the current title is "Unexpected Trypanosoma species…". I think the species found fit reasonably well with what it was expected. Furthermore, to me, the title should highlight the differences found in the two areas. This is to me the main ''selling point" of the article. He points out that the focus should be on the differences- see title suggestion
For all this, I'll recommend the editor to accept the manuscript, although I think it should be reviewed by the authors, specially the discussion section.

Thank you for the very good points that are highlighted, which improve the understanding of the manuscript. Therefore, following this strategies, comments and suggestions, besides those from the 2 other reviewers and the editor notes, we made significant changes in the structure and the main messages of the manuscript, starting from the aims through the methods, results and discussion sections.

"reviewComments"

This study estimated trypanosome prevalence and genetic diversity from two trypanosomiasis foci in Chad. There were no clear aims of the study outlined in the Introduction, other than to '*investigate the genetic diversity and interrelationship of trypanosomes present in these areas*' and no motivation as to why this would be important to do. As written, it therefore comes across as a bit of a 'fishing expedition' – while in some circumstances this may be acceptable, there is attempt in the manuscript to make comparisons between groups of individuals and sites based on the results but with no clear study design, power analysis or statistical tests to do this. While I acknowledge that potentially valuable data has been collected and should be published, I would recommend either a much clearer focus and justified approach, with detailed Methods, or a shorter paper/ note to detail the trypanosomes detected if the study is not powered to make comparisons. As I would recommend a major revision, I have not included here any minor comments on the manuscript. As summary of the major points is provided below:

- In the Introduction, a discrete set of aims should be provided that then relate to each section of the Methods.

We thank the reviewer for the remark. We provide the aims in the Introduction and objectives (line 123 to 133). Sections of Methods are now shifted in shorter forms from the supporting information S1, S2 and S3 Text to the main manuscript body.

- In the Results, human and cattle trypanosome prevalence data are presented without estimates of uncertainty, comparisons are made between a number of factors, but no details of the power analysis and study design with which to make these comparisons, or the statistical tests used, are provided in the Methods.

We incorporated the power analysis that we applied to get the sample size in both foci for humans and cattle using the open-source Epidemiologic statistics for public health software Version 3.01; knowing the human and cattle populations in these areas, as stated in the Manuscript (line 163-171 for the humans and line 178 to line182 for the cattle).
The study design is more detailed in the supporting information S2 Text from line 5 to line 18, and line 34 to 37. However, some parts of it are given in the manuscript (line 156 - 162 (for the humans) and line 173 to 178 (for the cattle)).
The statistical analysis applied in this study is provided in line 257 - 267. Estimates of uncertainty are also inserted in all trypanosome's frequencies.

- I noticed there are some p-values in the results, but it is not clear where these come from.

We thank the reviewer for pointing this out. Now it is clarified, including the description of the statistical analysis used as stated above.

- There is also insufficient reasoning as to why the detailed questionnaire data were collected, for either the cattle or the human population, or how study villages were selected. More detail needs to go into the main section of the Methods with respect to study design and power analysis -motivated by a clear set of aims – and then the statistics used to analyse the data.

Again, we thank the reviewer for suggesting that. As stated above, parts of the supporting information S1, S2, and S3 Text are now shifted in shorter forms into the main Method section. The villages were randomly selected (line 156 and line 174), as well as the breeders (line 175). However, random selection was biased (line 176) as the animals were partly chosen by the herdsmen themselves, presenting some animals apparently sick. This is mainly observed in the nomadic settlements. In the sedentary group, the herdsmen did not possess many heads; thus, a chosen herdsman would provide all of its animals unless they were absent during the blood collection.
Households were drawn, as indicated in line 158. As for the questionnaire for humans, the indices such as age, gender, health status, and previous and/or current infections including HAT, were addressed. This gave information on which group, there will be most trypanosomes evidence (line 309, line 590, and line 595). For the animals as well, age, gender, community structure (i.e., nomadic, sedentary), and breeding system etc. were addressed (line 183).

- In the Discussion, the authors state that HAT cases have been decreasing in Mandoul, but there was a resurgence of 23 new cases in Maro. From this the authors state that a more comprehensive picture of the situation is needed. This information is not provided in the Introduction, which may help with motivation,

The remark is considered (Introduction line 106 and line 116).

- But again, this still isn't clear justification for the necessity of the study given at the moment it neither fully focusses on genetic diversity, nor focusses on comparisons between the two foci.

We thank the reviewer for this suggestion. As included in the revised manuscript, the study aims are to investigate the circulating trypanosomes, including the occurrence of potentially zoonotic species in humans and livestock, and in their biological tsetse vector in two active HAT foci, Mandoul and Maro (Introduction line 123 - 125). This involves molecular identifications which will exhibit the genetic diversity of the species identified in the two areas. As the situation in the two foci is different (tsetse control existing in Mandoul and not started in Maro, or Maro is an open area bordering the CAR and Mandoul, an isolated area for tsetse habitat), we expected to observe differences in terms of trypanosomes diversity and tsetse population presence. And this has emerged from the data (we caught more tsetse in Maro while in Mandoul they are rare). As a particular note, there is a presence of pathogenic species in humans and cattle in Maro and their absence in the analysed samples of Mandoul. In this area, *T. theileri* is largely identified in cattle. Presence of atypical trypanosomes was observed in humans.

As recommended, significant changes have been made in all sections (aims, methods, results and discussions).

Click here to access/download
Revised Article with Changes Highlighted
Manuscript_Revised-Tracked.docx
