# Supplementary material for: Diversity of trypanosomes in humans and cattle in the HAT foci Mandoul and Maro, Southern Chad—A matter of concern for zoonotic potential?
Source: PLoS Negl Trop Dis. 2021 Jun 9;15(6):e0009323. doi: 10.1371/journal.pntd.0009323 (PMC8224965; doi:10.1371/journal.pntd.0009323)
Supplement: Attachment — Submitted filename: reviewComments.docx [file pntd.0009323.s015.docx]

This study estimated trypanosome prevalence and genetic diversity from two trypanosomiasis foci in Chad. There were no clear aims of the study outlined in the Introduction, other than to ‘*investigate the genetic diversity and interrelationship of trypanosomes present in these areas*’ and no motivation as to why this would be important to do. As written, it therefore comes across as a bit of a ‘fishing expedition’ – while in some circumstances this may be acceptable, there is attempt in the manuscript to make comparisons between groups of individuals and sites based on the results but with no clear study design, power analysis or statistical tests to do this. While I acknowledge that potentially valuable data has been collected and should be published, I would recommend either a much clearer focus and justified approach, with detailed Methods, or a shorter paper/ note to detail the trypanosomes detected if the study is not powered to make comparisons. As I would recommend a major revision, I have not included here any minor comments on the manuscript. As summary of the major points is provided below:

- In the Introduction, a discrete set of aims should be provided that then relate to each section of the Methods.
- In the Results, human and cattle trypanosome prevalence data are presented without estimates of uncertainty, comparisons are made between a number of factors, but no details of the power analysis and study design with which to make these comparisons, or the statistical tests used, are provided in the Methods.
- I noticed there are some p-values in the results, but it is not clear where these come from.
- There is also insufficient reasoning as to why the detailed questionnaire data were collected, for either the cattle or the human population, or how study villages were selected. More detail needs to go into the main section of the Methods with respect to study design and power analysis – motivated by a clear set of aims – and then the statistics used to analyse the data.
- In the Discussion, the authors state that HAT cases have been decreasing in Mandoul, but there was a resurgence of 23 new cases in Maro. From this the authors state that a more comprehensive picture of the situation is needed. This information is not provided in the Introduction, which may help with motivation, but again, this still isn’t clear justification for the necessity of the study given at the moment it neither fully focusses on genetic diversity, nor focusses on comparisons between the two foci.
