# Supplementary material for: Diversity of trypanosomes in humans and cattle in the HAT foci Mandoul and Maro, Southern Chad—A matter of concern for zoonotic potential?
Source: PLoS Negl Trop Dis. 2021 Jun 9;15(6):e0009323. doi: 10.1371/journal.pntd.0009323 (PMC8224965; doi:10.1371/journal.pntd.0009323)
Supplement: Attachment — Submitted filename: Response to reviewers.docx [file pntd.0009323.s016.docx]

**Responses to reviewers’ comments on "Unexpected Trypanosoma species in humans, tsetse, and cattle, identified in Southern Chad: Mandoul and Maro sleeping sickness foci" (manuscript ID PNTD-D-20-01868)**

The manuscript title is changed to: “Diversity of trypanosomes in humans and cattle in the HAT foci Mandoul and Maro, Southern Chad - A matter of concern for zoonotic potential?”

Please find below the detailed answers to the comments and questions of the reviewers. This also includes the 5 particular notes highlighted by the editor, and in the last pages, the answers to “reviewComments” received as a separate file.

First of all, we thank all the reviewers for their positive remarks, their agreement on the importance of the study, and their detailed review of the manuscript, including suggestions, comments, and questions.

**Methods**

**Reviewer #1:**

- A clear set of aims that link to each section of the Methods is not provided

We included more information concerning the study aims that link to the methods of this study. This study was undertaken to investigate the circulating trypanosomes, including the occurrence of potentially zoonotic species in humans and livestock, and in their biological tsetse vector in two active HAT foci, Mandoul and Maro. See Introduction from line 123 to line 133.

The detailed protocols in supporting material S1, S2 and S3 Texts are now shifted in shorter forms in the revised manuscript (Method sections from line 134).

- There is no clear study design or description of statistical analysis used to inform the results.

We provided additional information for the study design. See line 156 - 162 (for the humans) and line 173 - 178 (for cattle). More details are provided in S2 Text from line 5 to line 18, and line 34 to 37.

We want to thank the reviewer for the remark to include a description of statistical analysis used to inform the results. The statistical analysis applied is provided in line 257 to 267.

- No power analysis provided

Specification of the power analysis is included in the manuscript as recommended by the reviewer. See line 163 to 171 for the humans and line 178 to line182 for the cattle. We based on this power calculation, with an accepted margin of error of 5% and 95% confidence interval, to target the sample size per HAT focus (Mandoul and Maro) and per host (human and cattle). Unfortunately, in Mandoul only 78 cattle have been collected, by far less than the expected sample size due to an anthrax outbreak during a scheduled survey, as stated in the result part (line 315-316).

**Reviewer #2:**

- The methods need to include details on the sample frame for both human and animal, random sample method is stated but lacks details on either the number of samples that the survey set out to collect (cattle or human) or clear details on how the individuals were included for the sample at each field site.

We thank the reviewer for these suggestions. We included the information on the method of a random selection of households/herds for participation. See line 156 to 162 (for the humans) and line 173 to 178 (for cattle). More details are provided in see S2 Text from line 5 to line 18, and line 34 to 37.

The power calculation for the necessary sample size for the human survey is described from line 163 to 171, and for the cattle survey is from line 178 to line182. We faced an issue during the survey to collect cattle samples in Mandoul, because of an anthrax outbreak (stated in line 315 - 316). Therefore, we couldn’t reach the target sample size, as only a few samples (78) have been collected. However, the sample size was reached in Maro for both humans and cattle.

- In lines 432-435 of the discussion states that the owners presenting cattle for sampling [which is not random sampling. Or are you suggesting that herders could have separated healthy animals from the herd to only take unwell individuals to the sample site?

We thank the reviewer for the valid discussion point. Deviations of random sampling for cattle are now stated in the method (line 175 to 178) and then discussed in line 564 to 567. We further want to highlight that there are two aspects along that line for the animal selections. First, we aimed for a random study where we were able to select the herdsmen by following this strategy. However, when selecting the cattle, especially in the nomadic settlements, the selection has been influenced by the shepherds that select the animal based on their own interest (taking perhaps more sick than healthy animals). For the sedentary group, shepherds did not have many animals (4 heads approximately). Therefore, a selected herdsman will provide all its animals unless some are absent during the survey. As such, the random selection of the animals was biased. Nevertheless, the statistical analysis applied in the study was to compare variables in this cohort without making an extrapolation for the entire area.

- The statistical evaluation that is referred to in the results section has not been identified.

A description of statistical analysis applied to inform the results is provided in line 257 to 267.

- Results section refers to a questionnaire (line 284), which is absent from the methods.

The description of the questionnaire is now shifted from the S2 Text to the main manuscript (line 183 to 187). Responses to the questions are provided in excel file “Raw data_Cattle”.

- Cattle age forms part of the data analysis and discussion, how was the age of each animal established?

We thank the reviewer for the question. As stated in S2 Text (line 44), the age was given by the herdsmen and to some extent confirmed by the veterinarian present during the fieldwork. They are grouped in three categories young (<2.5 years), mature (between 2.5 and 5 years), and elders (above 5 years). Thus, the relative age given by the herdsmen will be within the age set subsequently.

**Reviewer #3:**

- Sample size and selection
  I tend to think that the methodology applied was appropriate for the study, although probably the objectives of the study should be stated more clearly. Particularly, I appreciate the sequencing, missing in other similar articles. On the other hand, sample sizes seem a bit low. Although clusters of villages, semi-nomadic, nomadic and military camps are mentioned, I’m not clear how participants (households and cattle) were selected/recruited: was there any randomisation that may allow to assume that the samples were representative? How comparable are the samples and the populations? Was there any sensitization campaign?

We thank the reviewer for the comments and questions.

- Sample size; selection; How comparable are the samples and the populations?

We based on the sample size proportion calculated using the open-source Epidemiologic statistics for public health software Version 3.01, knowing the human and cattle populations in these areas, as stated in the Manuscript (Line 163-171 for the humans and line 178 - 182 for the cattle). Sample sizes per focus (Mandoul and Maro) and per host (human and cattle) were obtained using this software with an accepted margin of error of 5% and 95% confidence interval. However, in Mandoul, the number of collected cattle samples was by far less than the expected sample size due to an anthrax outbreak, as stated in the result section (line 315-316).

We provided information on human and cattle selection. Line 156 - 162 (for the humans) and line 173 - 178 (for cattle). More details are provided in S2 Text (line 5 - 18, and line 34 – 37). For human surveys, households were drawn by chance. However, members of each selected household were automatically included, unless for individuals who did not consent to participate or children under 5 years old. The villages were selected randomly at each site, except the military camp that was at the request of its inhabitants.

For the cattle, the herdsmen (representing their herd) were selected randomly. However, the cattle were chosen partly by the herdsmen themselves for the nomadic settlements (precision added, line 176). For the sedentary group, breeders did not have many animals (approximately 4 heads or so). Therefore, a selected breeder will provide all its animals unless some of them were absent during the survey.

The comparability of the sample sizes and populations is crucial to our study. The human survey reached the required sample sizes (line 271) as calculated in advance (line 170), as well as the cattle in the Maro focus (Line 314 as calculated in line 181). However, for cattle from the Mandoul focus, the size is small (Line 314 as calculated in line 181).

- I tend to think that the methodology applied was appropriate for the study, although probably the objectives of the study should be stated more clearly.

We agree with the reviewer, and we have stated the objectives more concisely in the Introduction (line 123 to line 133).

- Was there any sensitization campaign?

Yes, the sensitizations have been done prior to the surveys where the team visited the locations and made contact with the local communities. In these visits, the objectives of the study were explained. This was added in S2 Text Line 7. Furthermore, we repeated the meetings during the recruitment day, a day prior to the blood collections.

- Ethical considerations
  How were cases managed (i.e. human or cattle +ve samples?  For example, were they and PNLTHA notified?

This work was supported in the beginning by both PNLTHA and IRED, as mentioned in the Acknowledgments section. The personnel of these institutions are well known in these foci and are working in close contact with the human and animal health services, as well as the local communities. Therefore, suspected human cases were automatically taken care ofby the local health services which follow the protocol of the diagnosis and treatment depending on the phases of the disease if the cases are confirmed.

- Were +ve cattle treated, not able to recover…

For the sick cattle, especially those suspected for AAT, a veterinarian who was supporting the team and the local veterinarian technician manage to treat the animals. Also, this depended on the protocol of treatment depending on the health status and symptoms of the animal, recommended by this animal health personnel.

All these parallel supports were discussed with the authorities in charge of Human and animal health sector, including PNLTHA, the Regional delegations as well as the local Human and animal health services, and the administrative authorities.

- Study areas:
  It would be relevant to describe the main differences between the Mandoul and Maro foci/habitats. For example:
  - Mandoul: Tsetse are restricted to the swaps formed in the southern limit of the Mandoul river. The tsetse distribution is limited in the South by the springs. As the river flows north, the swamp deteriorates into a marsh habitat, unsuitable for tsetse. Therefore, the population is isolated. Vector control operations with annual deployment of Tiny Targets started in 2014.
  - Maro: In the southmost sections of Chari/Sido rivers, where rivers mark the border with CAR. Tsetse habitat configured by is the thin riverine vegetation along the banks of the rivers. Vector control operations started in the Chadian part in 2018, with annual deployments of Tiny Targets. No similar operations have been implemented across the border.

We thank the reviewer for the suggestion to include this important information. It is included (Line 114 -117 and line 142 - 152) and more details are in S1 Text.

- Trypanosomes in tsetse:
  Any reason not to analyse salivary glands for mature infections of *T. brucei*?

It was one of our interests. Unfortunately, most of the tsetse flies trapped were found dead due to the high temperature and the low relative humidity during the surveys in these regions (Line 464). Therefore, we faced a technical issue for dissecting the salivary glands and screen for mature infection of *T. brucei*.

**Results**

**Reviewer #1:**

- Due to the absence of a discrete set of aims and the absence of details of study design (e.g. justification for selection of villages/ individuals), power analysis, or statistical analysis the results do not match the analysis plan. There are no estimates of uncertainty provided for trypanosome prevalence.

Overall, the study aimed to look for the circulating trypanosomes in the three host from the two HAT foci and their genetic diversity. Since the selection of cattle especially was biased, a stating prevalence cannot be adapted for the study to extrapolate for that of the whole population. However, it might be possible for the human section. Therefore, the term “prevalence” was not used but instead “frequency” from our point of view is suitable. Estimates of uncertainty (using the Clopper-Pearson binomial test with the lower and upper limits of the 95% confidence interval) was now added to all frequencies of trypanosomes. Power and statistical analysis are now included.

**Reviewer #2:**

- Due to the two study areas being detailed I suggest the author presents each set of findings first as overall findings (2 areas combined) then Maro and Mandoul individually. Thereby standardising the order. To highlight the issue for a reader unfamiliar with the study; between line 174 and 177 it was not until I had already been confused by the reported percentages 0.5 and 2.7 (which I thought should have been 0.28 and 1.53) that it becomes clear the results are only Maro and not the whole study.

We want to thank the reviewer for the suggestion. It is more clarified now (e.g., line 283).

- Attention needs paying to typographical errors, to give some examples: Line 258 'at the tree time points' and line 341 where 'of' is missing after 13%.

Thank you to remained us for typographical errors. We corrected several spelling errors starting from the abstract through the conclusion.

- Further, throughout the results section the decimal places used in numbers is inconsistent.

The suggestion is taken into account. We thank the reviewer for this precision.

- There are sections in the results that belong in the discussion. examples; line 262-263 and 276-278.

We moved them.

- Figure 1, the colour used for tsetse trap location is very hard to identify against the green base map, also the text in the image seems to be quite blurred.

We thank the reviewer for this remark. We improved all figures.

- Table 1 should be in the results section and referred to in the discussion.

We moved it and added a paragraph for its description (Line 510 to 523).

**Reviewer #3:** Human cases

- Two human cases of *T. b. gambiense* in Maro seems to suggest a relatively high prevalence.  Do the authors consider that this may be a good representation of the prevalence in the foci? Or, may this relate to the low sample size and/or bias in the selection of participants? Or, ‘oversensitivity’ of the technique?

The households were randomly selected, and the sample size sufficient to represent the population in the areas. We used the term frequency for the whole study just for the consistency of the manuscript, since the cattle selection was biased. However, we could extrapolate that the *Trypanosoma* prevalence in the entire population of Maro is high. This could also be influenced by the techniques used since nested PCR can amplify traces of DNA (down to 10 pg).

- I assume the main point of this finding is proving the presence of *T. b. gambiense*?

While *T.b.gambiense* is the main health concern, we also want to raise awareness of the potentially zoonotic species.

- Do the forms include a question about previous travels?

We thank the reviewer for the interesting discussion point. Yes, and also other information such as previous HAT infection and current health status or age and gender (see comment in the discussion part line 590 and line 595).

- Tsetse:
  Species not mentioned.

We agree that this might be useful information, but as tsetse were not the focus of the manuscript, we found this too much to be described. Nevertheless, it is now incorporated (Line 202 - 203 in the method section and Line 462 - 463 in the result part).

- Figures
  Figures are small and low definition.  They are difficult to read.

They are now adjusted for better visibility.

**Conclusion**

**Reviewer #1:**

We want to inform you that the discussion section is revised as it was recommended, and major modifications have been incorporated.

- It is not made clear why this study helps to better understand the situation in Chad. Without detailed methods and robust comparison between the two foci in the results, no conclusions can be drawn with respect to the differences between Mandoul and Maro.

We want to investigate the diversity of trypanosomes and see if Maro have the same pattern of the species diversity since the tsetse control has not been primed at the time of the survey like it was for Mandoul focus. In conclusion, as the aim is stated and methods described, the results showed a difference between the two areas in terms of species diversity and also in terms of tsetse fly presence. This could be due to the vector control initiated in the Mandoul which decreases the tsetse population (as only a single fly was caught see line 518) and thus the pathogenic trypanosomes (line 522, Table). It could also be due to the geographic location of the Maro bordering the Central African country, involving transhumance activities.

**Reviewer #2:**

We want to inform you that the discussion section is revised as it was recommended, and major modifications have been incorporated.

- line 679-680: Tiny targets are only used as tsetse control not elimination, also note tinny is a spelling error.

Thank you for pointing this out, it is corrected.

- Line 684-686: Maro is now receiving parasitological evaluation and regular control of tsetse (as you state in line 638).

The following note was deleted as the discussion is recast. Nevertheless, the message appears in line 717.

- The close of the conclusion is stretching. The assertion that to achieve the HAT elimination goal atypical trypanosomes need considering is at odds with the earlier statement in lines 399 - 403 where you highlight that the occurrence of non-HAT species of trypanosome detected in a human is likely the detection of an unviable infection.

The conclusion is now recast, see Conclusion line 767. The discussion of atypical HAT versus detection artefacts is crucial. Of course, we do not know if the trypanosomes (including atypical species) observed in humans using sensitive molecular techniques are transient or established infections. But the presence of much diversity in human blood samples is worrying, especially in the case of the individual from the Mandoul focus with an unknown *Trypanosoma sp*. However, since this man previously had a HAT-gambiense infection and was cured of it but has still sequels of the disease, and showed evidence of an unknown trypanosome, this situation needs to be closely monitored. It must not be neglected, as it might be an opportunistic infection. Note: we asked those in charge of supervising the HAT-disease especially the personnel of Bodo’s hospital (local health service personnel who are working in close contact with the National Program, PNLTHA), they confirmed that he is HAT-negative for all the last screening campaigns.

**Reviewer #3:**

We want to inform you that the discussion section is revised as it was recommended, and major modification have been incorporated.

- 385-388 “To evaluate risk assessment of HAT, regular screening campaigns of humans for *T. brucei gambiense* using microscopy, Loop-mediated isothermal amplification (LAMP) and RDT-kit have been undertaken by the Ministry of public health and its partners within the historical HAT-foci in Southern Chad”. At the moment, case definition in Chad for HAT is based on CATTs test at certain titer dilution (I think 1/16). Other direct (e.g. LAMB, mAECT) or indirect (e.g. trypanolisis) tests are not as common as in other countries.

The description is modified see 583 to 587. We thank the reviewer for the suggestion.

- 397-399 “It is the first study conducted in two Chadian HAT foci using a molecular identification tools, i.e. ITS1 amplification supported by gGAPDH analyses and sequencing to screen at the same time the tsetse fly vector, as well as human and cattle as definitive mammalian hosts”. I know there are differences, but please see https://www.parasite-journal.org/articles/parasite/full_html/2020/01/parasite200101/parasite200101.html

Thank you for bringing the study to our attention which we included in the discussion section (line 589). There are still differences in the approach of the two studies as well as the target hosts. Nevertheless, it gave some other values concerning the presence of *T. b. gambiense* in the foci.

- “Diversity and distribution of trypanosomes in the area”:
  431-432 “The evidence of high trypanosome frequency in cattle (see S5 Table for details) vigorously supports our observation of high frequency in tsetse flies”  In my view, the data shared by the authors show a clear distinction between the findings between Mandoul and Maro.  Thus:
  Mandoul:
  - Hard to find flies (because probably there are not many)
  - T. theileri is predominant in cattle (transmitted mostly by tabanids), with some T. vivax (which can be transmitted by other biting insects)
  - Neither T. congoloense nor T. brucei found (transmitted by tsetse)
  - Do the above imply that the incidence of tsetse-transmitted tryps might be approaching zero?

From this investigation, we can assume that in the Mandoul area, Tiny Target's implementation reduced the tsetse population and thus trypanosomes which this vector can transmit. This has also been stated in an already published paper (Line 146 and 714, Mahamat et al., 2017). Possibly tsetse-transmitting tryps might be approaching zero. However, more data and coverage from other sites in the focus than those visited (by including tsetse, humans, and reservoirs) could give more light to this assumption.

- Maro:
  - Tsetse were collected in relatively good numbers
  - T. congolense predominant
  - More variability in Tryps spp
  - Do the above suggest that in Maro tsetse are vectoring human and cattle Tryps?

We draw the conclusion that yes indeed this is suggested, see Discussion line 690, 717 and 724.

- Could the vectors explain the differences in the seasonality for each Tryp spp?

Not for all trypanosome species, but certainly for *T. congolense* and *T. brucei* as it is already known that in the dry season when the tsetse populations are reduced, their incidence in the herds is also reduced. Nevertheless, the frequency of *T. vivax* could be high in the dry season as other biting insects could also transmit it (Line 750 - 756). Therefore, this could also be the case for the other trypanosome species that other vectors can transmit.

- The authors mention in the discussion that cattle participating in the study might have been biased, as perhaps owners presented sick animals hopping for a treatment.  This might explain a relatively high infection rate in cattle. Was there any bias in the selection of human participants? (see above)

See line 156 to 162 (for more details see S2 Text line 14-18: “In order to proceed with the selection, households were numbered and a total of 6 to 16 households (see the paragraph below) in a selected village were drawn for participation. A chosen household included all its members automatically”.

The number of the households per village depends on the number of members of selected households. *i.e.* if one household has for instance 16 members, the chance to include many following households in the same village is reduced, since we targeted to include maximum of 70 participants per village.

- 440-442 “The relevant result emerging from the data is the high frequency (Table 1) and diversity (see S5 and S7 Tables for details) of trypanosomes in cattle in both foci”. As mentioned above, I don’t see it that way.  I think the most relevant result is precisely the differences between both sites.

Thank you for dragging our attention on that aspect. For this reason and those mentioned above, we reconstructed the discussion, which now includes sections that follow the suggestions, *e.g.,* line 568 and 573.

- 442-444 “Another finding and not the least was that, in the Mandoul focus *T. theileri* was abundant, whereas only very few *T. vivax* and *T. simiae* were found and there was no evidence for *T. congolense*, neither in cattle nor in humans”.  This seems a repetition of the results without the discussion.  How do the author explain this result?  See above

This is taken into account in the new discussion, and the tendency of trypanosomes presence or absence in a focus is explained (line 722-736).

- “Diversity of known trypanosomes”
  446-450 “The *T. b. gambiense* DNA that was detected in one child and one old man in the Maro focus, confirmed the presence of the parasite in the area…”.  The authors state that the two cases proved the presence of *T. b. gambiense* in Maro. Instead of “Interestingly, none of the samples collected in Mandoul was *T. b. gambiense* positive, and this is in agreement with the reduction of its incidence reported by Mallaye” the author should be consistent with the way reporting for Maro, i.e. confirmed presence vs. non-confirmed presence.  Is this “in agreement with the reduction in the incidence”?  Probably yes, but this is not proof of it.  With this small sample size, rather than using the data to infer an epidemiological situation, I think it would be more honest to use the results in terms of proven or non-proven presence of the parasite.

Thank you for the suggestion which leads us to modify the structure of the discussion (line 587 - 594).

- 456-459 “Interestingly, *T. vivax* clustered in two clades, with both present in samples collected from the Maro focus. One of these clades groups with the East African *T. vivax* (EA) with a strong homology and high similarity. This clade EA was described for the first time in Tanzania [49], then in Nigeria [28] and in Cameroon [31] in 2019, and now in Chad in this study”. If the clade has been identified in Nigeria and Cameroon, why is it so surprising finding it in Chad?

In the literature, it was identified as the East African clade, and there is evidence of its presence in West and Central Africa reported recently. Therefore, we wanted to point out that this is a clade not restricted only to Eastern Africa, and its identification could be due to the sequencing data that are now more involved (Line 643).

- Any speculation about how the dispersion and diversity of *T. vivax* across Africa?

*T. vivax* is widely spread, including tsetse-free areas as well as many animal species such as camel, cattle, etc. are infected. The available sequences in the database and previous reports pointed out so far from our knowledge, the two clades (EA and AA) with a mention of large distribution of the A/A clade as mentioned in line 647. So, thorough sequencing of samples from different locations and origin in Africa might lead to identifying other clades (subspecies) as already reported in the literature (Adam et al., 2010).

- 476-477 “*T. theileri* was not observed in the tsetse samples which confirms its transmission independently from tsetse.” The sentence if quite vague.  We know that *T. theileri* is mostly transmitted by tabanids. In my opinion, the main point to be made about *T. theileri* in Maro and Mandoul is that the predominant Tryps spp in Mandoul is not transmitted by tsetse (because tsetse are close to annihilation); on the other hand, Tryps spp in Maro are more diverse.

Thank you for suggesting this. It is taken into account (line 579).

- 487-489 “The identification of *T. godfreyi* and *T. simiae* in different tsetse fly tissues and cattle, common across the African continent [56], [57] suggests that the vector would have fed on wild animals such as warthogs”. Why??  The population of domestic pigs are far larger than that of warthogs.  Presumably, T. simiae are a problem for pigs.

This part was found less necessary and was deleted from the revised discussion.

- 513-514 “The more the animals get old, the more their susceptibility to pathogenic species is high, as age-related resistance to trypanosomes is recognised” As explained by Vale, Torr and others, the body mass of the host is directly correlated to the attraction of tsetse; thus, old (and heavy) cattle attract more tsetse than calves.

Thank you for the suggestion. See line 410.

- Relevance for the area(s)
  624-629: If by the “elimination campaign” the authors refer to the use of SIT, as far as I know, this has not started yet.  No release of sterile males was done during the time reported in this manuscript.  The elimination project (using SIT) was attached to an existing project aiming to eliminate HAT transmission (not necessarily tsetse).  And yes, that is being done using Tiny Targets, although PATTEC has nothing to do with it.

This is essential information. The “elimination campaign” term is changed with tsetse control when it addresses the issue of tsetse.

- 638-639: There isn’t any tsetse elimination campaign in Maro.  There is a tsetse control operation, aiming (in addition to case detection and control) to eliminate the transmission of HAT.  It started in 2018.  Note than the river is the natural border between Chad and CAR (sometimes the river is just 5-10 m wide, some time a few hundreds) and all the efforts were done on the Chadian side, only.

The correction has been made concerning the tsetse control, e.g. line 717.

- Conclusions:
  677 It might be worth mentioning somewhere that the current WHO goal is the “elimination of HAT transmission” by 2030.

The correction has been made see, line 768-769.

- 679 As above, the aim of the Tiny Targets is not the elimination of tsetse.  In Mandoul, and Mandoul only, there is a new project to implement SIT, but (if I’m not wrong) the release of sterile males has not started yet.

The correction has been made see above.

**Editorial and Data Presentation Modifications?**

**Reviewer #1:**

- (No Response)

**Reviewer #2:**

- Attention needs paying as references are out of order, eg: line 629-630 references Mahamat et al [23] but in the references on line 777 [23] is Targeting Tsetse on LSTM's website.

Thank you for the kind remark. We check all the references, including the stated ones. With the restructuration of the discussion, some of the references are deleted.

**Reviewer #3:**

- 68-69 “Despite the WHO goal to eliminate it by 2020, HAT is still a public health problem, because 70 million people in 36 sub-Saharan African countries are at risk of infection”.  Actually, the 2020 goals established by WHO were globally achieved by 2018; that was according to their own definition of “public health problem” (i.e., <1/10,000 cases, >90% of endemic foci; and <2,000 cases worldwide). The current goal is the elimination of transmission.

Description modified see line 73. Thank you

- 87-89 “The main insect vector in Africa are flies of the genus Glossina (Glossinidae: Diptera). However, the parasites can also be transmitted mechanically by other biting flies such as tabanids and Stomoxys”.  Although, this is true for some Trypanosoma spp (e.g. *evansi*, *theileri*, *vivax*…), I find the statement a bit too general.

We prefer to leave it like this from our point of view as it gave a global idea to the reader (line 92). However, we add a sentence concerning *T. theileri.*

**Summary and General Comments**

- Use this section to provide overall comments, discuss strengths/weaknesses of the study, novelty, significance, general execution and scholarship. You may also include additional comments for the author, including concerns about dual publication, research ethics, or publication ethics. If requesting major revision, please articulate the new experiments that are needed.

**Reviewer #1:**

- Please see attached document.

The comments into document are all taken in consideration in this letter (see in the last sections below).

**Reviewer #2:**

- This represents interesting and insightful work, which I want to see published.
  There are aspects of the paper that need refining which will greatly benefit the reader.
  Details of some methods that have been used are absent from the appropriate section.
  Results section will benefit from being revised.
  The assertion that atypical trypanosomes could threaten HAT elimination should be accompanied by a reminder of your sentiment in lines 399-403 thereby ensuring the reader takes away an unbiased conclusion.

We thank the reviewer for the positive remarks. The suggestions were considered.

- From my knowledge the fieldwork was undertaken for this project predates work on eradicating tsetse in Mandoul, there was ongoing tsetse control by the consortium of PNLTHA, FIND, IRD and LSTM as part of the drive against gHAT. As such reference to eradication in Mandoul is misleading and should be reworded as ongoing tsetse control. The article repeatedly refers to the FIND, IRD, LSTM operation as tsetse elimination, which is inaccurate, the project is objective to control tsetse to thereby reduce the risk of infection in gHAT foci and not to eliminate tsetse, this should be addressed throughout.

We thank the reviewer for remaining us the important terms. “tsetse elimination” term is replaced in the revised version by “tsetse control”.

- Abstract refers to trypanosomosis in humans, this should be trypanosomiasis for disease in humans.

Corrected was done, see Abstract line 19. Thank you

**Reviewer #3:**

- The authors present in the manuscript a comprehensive study of the two main HAT foci in Chad, including parasitology, epidemiology, entomology, population genetics…  The amplitude of the study has advantages and disadvantages.  On the one hand, it is very informative (the authors report the Tryps spp circulating in both foci), but on the other hand, it lacks an obvious ‘selling point’ that may appeal potential readers.  After reading the manuscript with interest, in my opinion, the data contains some implications that are not properly highlighted and discussed, and other perhaps less important with too much detail.  The weakest part is probably the discussion, when the authors have the chance to convince readers why their work is relevant.  It is indeed useful to know the Tryps spp circulating in humans, cattle and tsetse vectors, but in practical terms, some spp are more important than others.
  For example, the authors dedicate extensive paragraphs to discuss things like ‘atypical HAT’, or Tryps spp than hardly cause any symptoms in livestock; spp of less medical or veterinary importance can be mentioned briefly, and expand on, for example, T. b. gambiense, T. b. brucei, T. congolense, T. vivax and perhaps, T. simiae.  Conversely, what it is probably the most interesting points of the research are not discussed in the length they deserved.
  My first advise for the authors would be to recognise the weakest points in the data, and highlight the strongest ones.  For example, the sample size seems low, and probably the recruitment of cattle and people were not randomised.  That means, the %s reported may not represent the whole population (not valid for epidemiological extrapolations).  That is not a big problem, as they can still report relative frequency of the different parasites (in flies, cattle and human), and for those less common, presence confirmed vs. presence non-confirmed.

As a reader, I would like to see the differences between both foci: what those differences are and why.  To show this, the reader needs to understand from the beginning what makes Mandoul and Maro so different:
- As a tsetse habitat, Mandoul represents an ecological island: it is isolated, and the risk of reinvasion is very low.
- Before 2014, when the only approach to control HAT relied solely on case detection and treatment, Mandoul was by far the most active HAT focus in Chad (some numbers would be good).  The addition of vector control reduced dramatically the number of cases to a handful.
- As the number of cases in Mandoul declined, PNLTHA increased the efforts to identify new cases in areas that up to that moment were considered of less importance (including Maro).  I’m quite convinced that the increase in the number of cases in Maro was the result of the additional efforts (at least in part).
- The tsetse habitat in Maro, on the other hand, is open for reinvasions.  The deployment of targets started in 2018 and probably did not have any impact in this study.  In the long term and due to the differences, we don’t expect to have the same results as in Maro.
With this background, the data suggest that the presence of Tryps in Mandoul can be explained without tsetse, but the opposite is true in Maro, whereas in Maro tsetse-transmitted Tryps were found in larger proportions, and the spp variability was much greater.  The difficulties to collect tsetse in Mandoul seems to support this idea.  Tsetse-transmited Tryps are of medical or veterinary importance (e.g. T. b. gambiense, T. congolense…), so that looks like a good thing.  This could lead into a subsection in the discussion: practical implications; or what we need to do in Maro, so it can look more like Mandoul.
This contrasts with the recently published paper by Vourchakbe et al
<https://www.parasite-journal.org/articles/parasite/full_html/2020/01/parasite200101/parasite200101.html>
Vourchakbe and colleagues reported a higher proportion of T. b. g. in Mandoul, compared to that of Maro (Tryps found in goats, sheep, dogs and pigs, and they did not sequence the amplicons).
Probably there isn’t enough data to support big statements about potential trypanotolerant cattle breeds.  We also need to consider other variables associated with breeds: for example, Arab zebu might look like trypanosensitive according to infection rates, but as the breed of nomadic heads, they are also exposed to different risks.  Cattle are specially exposed when they cross rivers, and nomadic cattle does that more often than stable cattle.  Another factor to consider (commented briefly by the authors) is the potential correlation between seasonality of certain Tryps spp and their vectors (e.g. tsetse, horseflies…).  And, talking about vectors, the authors should mention the name of the tsetse, at least once: Glossina fuscipes fuscipes.
If the authors agree with me, they should probably indicate more clearly in the introduction the objectives of the study (e.g. to compare presence/absence of different Tryps spp -- in cattle, people and flies -- in Mandoul and Maro, or something along those lines).  Then, the methods will fit with the objectives easily.
In agreement with that, I would also recommend a change in the title.  First, I’m not sure why the current title is “Unexpected Trypanosoma species…”.  I think the species found fit reasonably well with what it was expected.  Furthermore, to me, the title should highlight the differences found in the two areas.  This is to me the main ‘’selling point” of the article. He points out that the focus should be on the differences- see title suggestion
For all this, I’ll recommend the editor to accept the manuscript, although I think it should be reviewed by the authors, specially the discussion section.

Thank you for the very good points that are highlighted, which improve the understanding of the manuscript. Therefore, following this strategies, comments and suggestions, besides those from the 2 other reviewers and the editor notes, we made significant changes in the structure and the main messages of the manuscript, starting from the aims through the methods, results and discussion sections.

**“reviewComments”**

This study estimated trypanosome prevalence and genetic diversity from two trypanosomiasis foci in Chad. There were no clear aims of the study outlined in the Introduction, other than to ‘*investigate the genetic diversity and interrelationship of trypanosomes present in these areas*’ and no motivation as to why this would be important to do. As written, it therefore comes across as a bit of a ‘fishing expedition’ – while in some circumstances this may be acceptable, there is attempt in the manuscript to make comparisons between groups of individuals and sites based on the results but with no clear study design, power analysis or statistical tests to do this. While I acknowledge that potentially valuable data has been collected and should be published, I would recommend either a much clearer focus and justified approach, with detailed Methods, or a shorter paper/ note to detail the trypanosomes detected if the study is not powered to make comparisons. As I would recommend a major revision, I have not included here any minor comments on the manuscript. As summary of the major points is provided below:

- In the Introduction, a discrete set of aims should be provided that then relate to each section of the Methods.

We thank the reviewer for the remark. We provide the aims in the Introduction and objectives (line 123 to 133). Sections of Methods are now shifted in shorter forms from the supporting information S1, S2 and S3 Text to the main manuscript body.

- In the Results, human and cattle trypanosome prevalence data are presented without estimates of uncertainty, comparisons are made between a number of factors, but no details of the power analysis and study design with which to make these comparisons, or the statistical tests used, are provided in the Methods.

We incorporated the power analysis that we applied to get the sample size in both foci for humans and cattle using the open-source Epidemiologic statistics for public health software Version 3.01; knowing the human and cattle populations in these areas, as stated in the Manuscript (line 163-171 for the humans and line 178 to line182 for the cattle).

The study design is more detailed in the supporting information S2 Text from line 5 to line 18, and line 34 to 37. However, some parts of it are given in the manuscript (line 156 - 162 (for the humans) and line 173 to 178 (for the cattle)).

The statistical analysis applied in this study is provided in line 257 - 267. Estimates of uncertainty are also inserted in all trypanosome’s frequencies.

- I noticed there are some p-values in the results, but it is not clear where these come from.

We thank the reviewer for pointing this out. Now it is clarified, including the description of the statistical analysis used as stated above.

- There is also insufficient reasoning as to why the detailed questionnaire data were collected, for either the cattle or the human population, or how study villages were selected. More detail needs to go into the main section of the Methods with respect to study design and power analysis -motivated by a clear set of aims – and then the statistics used to analyse the data.

Again, we thank the reviewer for suggesting that. As stated above, parts of the supporting information S1, S2, and S3 Text are now shifted in shorter forms into the main Method section. The villages were randomly selected (line 156 and line 174), as well as the breeders (line 175). However, random selection was biased (line 176) as the animals were partly chosen by the herdsmen themselves, presenting some animals apparently sick. This is mainly observed in the nomadic settlements. In the sedentary group, the herdsmen did not possess many heads; thus, a chosen herdsman would provide all of its animals unless they were absent during the blood collection.

Households were drawn, as indicated in line 158. As for the questionnaire for humans, the indices such as age, gender, health status, and previous and/or current infections including HAT, were addressed. This gave information on which group, there will be most trypanosomes evidence (line 309, line 590, and line 595). For the animals as well, age, gender, community structure (i.e., nomadic, sedentary), and breeding system etc. were addressed (line 183).

- In the Discussion, the authors state that HAT cases have been decreasing in Mandoul, but there was a resurgence of 23 new cases in Maro. From this the authors state that a more comprehensive picture of the situation is needed. This information is not provided in the Introduction, which may help with motivation,

The remark is considered (Introduction line 106 and line 116).

- But again, this still isn’t clear justification for the necessity of the study given at the moment it neither fully focusses on genetic diversity, nor focusses on comparisons between the two foci.

We thank the reviewer for this suggestion. As included in the revised manuscript, the study aims are to investigate the circulating trypanosomes, including the occurrence of potentially zoonotic species in humans and livestock, and in their biological tsetse vector in two active HAT foci, Mandoul and Maro (Introduction line 123 - 125). This involves molecular identifications which will exhibit the genetic diversity of the species identified in the two areas. As the situation in the two foci is different (tsetse control existing in Mandoul and not started in Maro, or Maro is an open area bordering the CAR and Mandoul, an isolated area for tsetse habitat), we expected to observe differences in terms of trypanosomes diversity and tsetse population presence. And this has emerged from the data (we caught more tsetse in Maro while in Mandoul they are rare). As a particular note, there is a presence of pathogenic species in humans and cattle in Maro and their absence in the analysed samples of Mandoul. In this area, T. theileri is largely identified in cattle. Presence of atypical trypanosomes was observed in humans.

As recommended, significant changes have been made in all sections (aims, methods, results and discussions).
