# Supplementary material for: Diversity of trypanosomes in humans and cattle in the HAT foci Mandoul and Maro, Southern Chad—A matter of concern for zoonotic potential?
Source: PLoS Negl Trop Dis. 2021 Jun 9;15(6):e0009323. doi: 10.1371/journal.pntd.0009323 (PMC8224965; doi:10.1371/journal.pntd.0009323)
Supplement: Attachment — Submitted filename: Response to Reviewers_190321.docx [file pntd.0009323.s018.docx]

**Responses to reviewers’ comments on " Diversity of trypanosomes in humans and cattle in the HAT foci Mandoul and Maro, Southern Chad - A matter of concern for zoonotic potential? " (manuscript ID PNTD-D-20-01868R1)**

Please find below the detailed answers to the comments and questions of the reviewers. All the particular notes highlighted in the pdf file are included in the manuscript.

First of all, we thank all the reviewers for their positive remarks, their agreement on the importance of the study, and their detailed review of the manuscript, including suggestions, comments, and questions.

<b>**Methods**</b></br></br>

- Reviewer #2: Methods are clear and well written.
  Study design is well set out and clear, with clarification on the overall population in the study area
  Ethical regulations are well set out as are the steps surrounding the participant's consent.
- There are a few editorial issues that should be crosschecked and cleared up - line 220 to 245 appear to have a different spacing in the PDF.

We thank the reviewer for this remark. It is corrected (line 255 to 292).

- Some of the terminology could be improved upon to be clearer I have included examples in the PDF.
  PCV should be detailed in the relevant 'blood collection' section of the methods.

All the remarks in the pdf file are taken into consideration. We thank the reviewer for this. The PCV detail has been introduced at the corresponding section. See line 222.

Reviewer #3: Comments and suggestions from the first review addressed adequately
--------------------

<b>**Results**</b></br></br>

- Reviewer #2: Analysis is described in methods with clearly presented results.
  There are some matters that I have tried to highlight as best I could in the PDF attached.

The remarks in the pdf file are taken into account. Thank you.

- The inclusion of the military camp was requested and should be reported in the findings but, as it was not part of the study design these samples should be separated from the study population in the results and analysis.

Thank you for the suggestion. We separated the military camp result from the study population. Therefore, the prevalence has slightly changed (see line 338 to 341 and table 2 line 540).

- Reviewer #3: Comments and suggestions from the first review addressed adequately
  --------------------

<b>**Conclusions**</b></br></br>

- Reviewer #2: Conclusions are well thought out and supported. with presentation of the public health importance and highlighting specific findings of this study that do merit follow up investigation - both in human and animal health.
- I am cautious about the presentation of the detection of *T. congolense* DNA in a human sample. The DNA presence is likely to be due to an unsuccessful infection that the individual has cleared. To be classified as an atypical-human trypanosome parasitological confirmation is required. There is a very strong case for follow up on the individual, but until that is done the issue is a peculiarity and not 'alarming' as described in line 564.

We thank the reviewer for this point of view. We removed the term “alarming” and gave some suggestions in order to confirm the presence by direct microscopy as described in line 592 and line 663.

- The treatment of animals with trypanocides is an interesting point that might add clarity to the situation with AAT. If the questionnaire (described in the methods) did capture any data on trypanocide usage it would be a strong addition, even 'unstructured interview' evidence of treatment or not that may have been given by cattle owners would be an interesting addition.

Yes, during our surveys, there is a part of the questionnaire that addressed the issue of drugs (often used) and treatment of the animals (by the owner or the veterinarian/technician). The trypanocides are among the most used drugs as also observed on the open weekly marked visited during our stay in the area. The drugs are quite often administered by the herdsmen. We did not include it because of the manuscript length. This is the only reason. However, we included it now (line 670-671) as you suggested it. Thank you for this.

- I would really like to know if the 2 people that were detected by PCR as having evidence of gHAT have been followed up - or are planned to be followed up, and the conclusions would be a nice place to have that.

Thank you for this remark. We did not meet the gHAT positive participants after have given the results and let them in contact with the health service which work in close contact with the PNLTHA because of ethical reason. However, we did ask the health center’s responsible with whom these two participants were in touch, and he confirmed to us that further screenings for gHAT have been made. They are confirmed positive and treated from the parasite. Unfortunately, we did not ask for the stage of the disease and we did not have a form for that.

- Reviewer #3: Comments and suggestions from the first review addressed adequately.
  -------------------

<b>**Editorial and Data Presentation Modifications?</**b></br><br/>
Use  this section for editorial suggestions as well as relatively minor modifications of existing data that would enhance clarity. If the only modifications needed are minor and/or editorial, you may wish to recommend “Minor Revision” or “Accept”.

- Reviewer #2: very minor revisions to the body of text
- Reviewer #3: Some teeny-weeny comments:
- 103-104. “However, AAT has remained a major obstacle to its development, which employs more than 40% of the population”.  I don´t understand the sentence: does AAT employ 40% of the people in Chad?

We meant livestock sector which employ 40% of the population in Chad. It is now corrected (see line 106).

- 104-105.  “Chad also faces the public health problem HAT” I understand the sentence, although it sounds a bit odd.
- 118 “Animals may harbour the human pathogenic species, serving as a reservoir”.  Although there are some studies suggesting a significant role of animal reservoirs in the transmission of g-HAT, I don’t think this is 100% conclusive, e.g. see Rock et al 2017
- 615-616. “Sequence analysis of T. theileri gGAPDH sequences revealed several sub-groups…”  Remove first “Sequence”

Thank you for these remarks. It has been deleted (line 714).

- 688-689 “...however, their implication on the transmission of the parasites in the area was not studied yet”.  Consider using the perfect tense (i.e. ‘has not been studied’), instead of the simple past (‘was’)

Thank you for this correction. We changed it (line 801).

--------------------
<b>**Summary and General Comments**</b></br></br>
Use  this section to provide overall comments, discuss strengths/weaknesses of the study, novelty, significance, general execution and scholarship. You may also include additional comments for the author, including concerns about dual publication, research ethics, or publication ethics. If requesting major revision, please articulate the new experiments that are needed.

- Reviewer #2: This is a very good presentation of your work, which will benefit from some very minor adjustments as detailed.

Thank you for the remarks.

- Reviewer #3: I am glad to see how the authors have addressed and incorporate most of the suggestions from the first review.  In my eyes, the current version of the manuscript makes an interesting reading, discussing between the findings in Maro and those in Mandoul (vis-à-vis the differences in the habitat, tsetse control operations, etc.).  This offers an informative and stimulating article for people interested in trypanosomiasis control.  Despite being relatively close, Maro and Mandoul illustrate two very different situations of HAT and AAT transmission; this is properly discussed in the ms.

Thank you for the positive point of view.

--------------------
